# Relation between Liver Transaminases and Dyslipidaemia among 2-10 y.o. Northern Mexican Children

**DOI:** 10.1371/journal.pone.0155994

**Published:** 2016-05-20

**Authors:** Maria del Mar Bibiloni, Rogelio Salas, Georgina M. Nuñez, Jesús Z. Villarreal, Antoni Sureda, Josep A. Tur

**Affiliations:** 1 Research Group on Community Nutrition and Oxidative Stress, University of Balearic Islands, E-07122, Palma de Mallorca, Spain; 2 CIBEROBN (Physiopathology of Obesity and Nutrition), Instituto de Salud Carlos III, E-28029, Madrid, Spain; 3 Faculty of Public Health Nutrition, Autonomous University of Nuevo León, 64460, Monterrey, Mexico; 4 Department of Health of the State of Nuevo León, 64000, Monterrey, Mexico; University of Milan, ITALY

## Abstract

**Background and Aims:**

The increase in overweight and obese children may be linked to increased rates of liver damage and dyslipidaemia. This study aimed to explore the associations of liver biomarkers with overweight/obesity and dyslipidaemia in Mexican children.

**Methods:**

The study was a population-based cross-sectional nutritional survey carried out in the State of Nuevo León, Mexico. The study included a 414 subjects aged between 2 and 10 years old (47.8% girls) who took part in the State Survey of Nutrition and Health–Nuevo León 2011/2012. Associations between alanine aminotransferase (ALT) and aspartate aminotransferase (AST), ALT/AST ratio, and major components of serum lipid profile were assessed.

**Results:**

Children with high ALT (defined as ≥P_75_) showed higher prevalence of dyslipidaemia than their counterparts, with high prevalence of high TChol (*P* = 0.053), non-HDL-chol, TG, and low HDL-chol. Children with an AST/ALT ≥T3 ratio were 0.43-times (95% CI: 0.25–0.74) and 0.27-times (95% CI: 0.17–0.44) low likely to be overweight/obese and to have dyslipidaemia than those with an AST/ALT <T1 ratio, respectively. The risk of high TChol, LDL-chol, non-HDL-chol and TG, and low HDL-chol levels also decreased in AST/ALT ratio groups.

**Conclusions:**

Our results pose the need for further investigation on whether AST/ALT may be useful as screening test in the assessment of children with cardiometabolic risk.

## Introduction

The liver is the major detoxification organ. The major functions of the liver are protein synthesis and bilirubin metabolism associated with bile production, carbohydrates and fat metabolism [1]. The aetiologies of abnormal liver function are variable: hepatitis caused by viral infection followed by non-viral infection; autoimmune, metabolic, toxic and anatomic liver diseases; and non-alcoholic fatty liver disease (NAFLDs) can be taken into consideration [1]. In a recent systematic review, the mean prevalence of NAFLD, a liver dysfunction associated with obesity and/or metabolic syndrome (MetS), in children and adolescents aged between 1 and 19 years from general population studies was 7.6% (95% CI: 5.5–10.3%) and 34.2% (95% CI: 27.8–41.2%) in studies based on child obesity clinics [2].

Clinically, the most common tests and specific indicators of hepatocellular necrosis to evaluate the degree of liver injury or liver disease are alanine aminotransferase (ALT) and aspartate aminotransferase (AST) [1,3]. When liver dysfunction occurs, the most common laboratory finding is an increase of ALT and AST. ALT is a more specific marker for evaluating liver damage than AST [1]. The AST/ALT ratio represents the time course and aggressiveness for disease that could be predicted from the relatively short half-life of AST (18 h) compared to ALT (36 h) [4]. Gamma glutamyl transpeptidase (γGT), which exists on the cell membrane of the liver (hepatocyte and bile duct cell) but also in a variety of organ tissues (kidney, pancreas, spleen, heart, brain, etc.) is also occasionally measured. However, although it has a high sensitivity, its specificity is low for hepatobiliary diseases [1].

Differences in studied ages, the cut-off values for high ALT levels and prevalence of overweight and/or obesity hampered direct comparison across studies. Previously, Purcell et al. [5] reported elevated ALT (defined as >40 U/L) in 6.6% of 8–19 year-old children and adolescents in Central Mexico (2004–2006). Larrieta-Carrasco *et al*. [6] also reported high ALT (defined as >40 U/L) in 7.9% of children aged between 6 and 12 years old in Mexico City (2008–2009). In 2012, the prevalence of obesity in 5–11 year old Mexican children was 14.6% [7] and the increased occurrence of obesity in children may be linked to high rates of liver damage and dyslipidaemia. Therefore, this study aimed to explore the associations of liver biomarkers with overweight/obesity and dyslipidaemia in Mexican children.

## Methods

### Study design

The study was a population-based cross-sectional nutritional survey carried out in the State of Nuevo León, Mexico (2011–2012).

### Study population, recruitment and approval

This study was part of the State Survey of Nutrition and Health–Nuevo León 2011/2012 (EESN-NL 2011/2012). The EESN-NL 2011/2012 was designed by the National Institute of Statistics and Geography (INEGI) to obtain information about the health and nutritional status of the population living in the State of Nuevo León (S1 Tables). The state was divided into four regions: northern, central, southern and the metropolitan area. Neighbourhood blocks were randomly selected and all subjects in all households were invited to take part in the survey (n = 1,180 per region; total = 4,720 households invited). A target of 1,059 households per region was assessed (89.7% participation) using the household as the sampling unit and an average of 2 interviews per household. The sample size was considered to be large enough to detect risk factors at regional level that had, at least, a prevalence of 8%, with a relative error calculation of 15% and a non-response rate of 40%. This sample size also calculated a prevalence of 9.0% in individuals aged between 0 and 9 years old, 10.0% in those aged from 10 to 19 years old, 5.0% in 20 to 59 year-olds, and 17% of the over 60 year old population. Information was obtained from 4,236 households and 7,290 individuals (1,372 0–9 year-olds; 1,319 10–19 year-olds; 3,125 20–59 year-olds; 1,474 ≥60 year-olds). One participant in the same age group living in the same household was selected. There were no socioeconomic differences between the people who participated in the survey and those who declined. Participation was similar in all age groups.

### Sample selection

A sample size of 640 households randomly selected from all assessed households was considered sufficient to detect risk factors with 95% confidence and a precision rate of 10%, and 466 children aged between 2 and 10 years were invited to participate. The analysis was limited to children who provided a fasting blood test with no missing anthropometric, serum lipid or liver transaminases data (*n* = 414, 47.8% girls, 88.8% participation).

### Ethics

The study was conducted according to the guidelines laid down in the Declaration of Helsinki. All procedures were approved by the Scientific Technical Committee and the Ethics Committee of the Public Health Nutrition Faculty at the Autonomous University of Nuevo León before the study began. Informed written consent was obtained from the next of kin, carers, or guardians on behalf of the children involved in the study.

### Anthropometric measurements

Height was determined to the nearest millimetre using a mobile stadiometer (SECA 213, Birmingham, United Kingdom), with the subject’s head in the Frankfurt plane. Body weight was determined to the nearest 100 g using a digital scale (Seca 813, Hamburg, Germany). Height and weight measures were used to calculate body mass index (BMI, kg/m^2^).

### Nutritional status definition

Normal weight, overweight and obesity were determined based on gender- and age-specific BMI cut-offs developed and proposed for international comparisons by Cole *et al*. [8,9], and also recommended by the International Obesity Task Force (IOTF).

### Biochemical measurements

Venous blood samples were obtained from the antecubital vein in suitable vacutainers after 12 hour overnight fasting conditions. Blood samples were centrifuged at 900 g at 4°C for 10 minutes. Serum ALT, AST, γGT, glycaemia, total cholesterol (TChol), high-density lipoprotein cholesterol (HDL-chol) and triglycerides (TG) were determined by enzymatic methods according to manufacturer’s recommendation (Roche Diagnostics, Mexico D.F.) using the Cobas 6000 analyser series (F. Hoffmann-La Roche Ltd, Basel, Switzerland) by the Metropolitan Hospital “Dr. Bernardo Sepúlveda” laboratory’s of the Ministry of Health of Nuevo León, Mexico [10]. Non-HDL-chol was calculated as TChol minus HDL-chol. Remnant-like particle cholesterol (RLP-chol) was calculated as TChol minus HDL-chol and LDL-chol.

### Liver enzymes definition

Data on liver enzymes were described through the distribution of ALT and AST/ALT ratio values into percentiles according to gender and age group (Table 1). ALT was modelled as a categorical variable, comparing children below and above the 75^th^ percentile (P_75_) for ALT. AST/ALT ratio was also modelled as a categorical variable, and children were grouped into tertiles according to their AST/ALT ratio.

**Table 1:** Alanine aminotransferase (ALT) and aspartate aminotransferase to alanine aminotransferase ratio (AST/ALT) centile values by sex and age in Northern Mexican children aged 2–10 years.

	*n*	Mean	SD	P_5_	P_10_	P_25_	P_33.3_	P_50_	P_66.7_	P_75_	P_90_	P_95_
Males												
ALT (IU/L)												
All	216	10.2	10.8	2.0	3.0	5.0	6.0	8.0	11.0	12.0	17.0	23.2
2–4 years-old	48	8.6	4.8	2.0	3.0	6.0	7.0	8.0	10.0	11.0	14.0	17.8
5–7 years-old	63	9.4	7.9	2.0	3.0	4.0	5.3	7.0	10.0	11.0	17.0	23.8
8–10 years-old	105	11.5	13.8	2.0	3.0	5.0	6.0	9.0	12.0	13.5	18.4	26.1
AST/ALT												
All	216	2.45	1.92	0.72	0.95	1.34	1.50	2.00	2.43	2.74	4.43	6.00
2–4 years-old	48	2.84	2.24	0.87	1.27	1.67	1.80	2.20	2.57	2.94	5.70	7.14
5–7 years-old	63	2.75	2.17	0.84	1.06	1.47	1.86	2.29	2.50	3.00	4.80	6.93
8–10 years-old	105	2.09	1.53	0.70	0.80	1.14	1.32	1.58	2.18	2.67	4.10	5.00
Females												
ALT (IU/L)												
All	198	12.1	15.5	2.0	3.0	6.0	7.0	10.0	13.0	14.0	19.0	25.0
2–4 years-old	46	11.3	8.1	2.0	2.7	5.8	7.7	10.0	13.3	15.0	20.9	25.7
5–7 years-old	76	14.2	23.5	2.0	3.7	6.0	8.7	11.0	13.0	14.0	21.0	36.2
8–10 years-old	76	10.4	5.9	3.0	4.0	6.0	6.0	10.0	12.3	14.0	19.0	22.3
AST/ALT												
All	198	2.08	1.52	0.68	0.87	1.17	1.29	1.64	2.14	2.50	4.21	5.00
2–4 years-old	46	2.44	2.11	0.59	0.82	1.14	1.30	1.67	2.50	2.68	5.00	7.83
5–7 years-old	76	2.13	1.48	0.68	0.91	1.24	1.38	1.67	2.09	2.49	4.33	5.15
8–10 years-old	76	1.81	1.02	0.68	0.87	1.13	1.21	1.41	2.00	2.32	3.38	3.91

### Adverse fasting glucose, serum lipid levels and dyslipidaemia definitions

Impaired fasting glucose was defined as fasting glucose ≥100 mg/dL. According to the 2011 Expert Panel on Integrated Guidelines for Cardiovascular Health and Risk Reduction in Children and Adolescents [11], serum lipid levels (mg/dL) were categorized into two subgroups (acceptable + borderline-high/low; high/low) as follows: TChol: acceptable + borderline-high <200, high ≥200; LDL-chol: acceptable + borderline-high <130, high ≥130; non-HDL-chol: acceptable + borderline-high <145, high ≥145; HDL-chol: acceptable + borderline-low ≥40, low <40; TG: acceptable + borderline-high <100, and high ≥100 in ≤9 year-old children, and acceptable + borderline-high <130, and high ≥130 in 10 year-old children; and RLP-chol (mg/dL): acceptable <P_75_ (<24.8 in boys, <27.0 in girls), and high ≥P_75_ (≥24.8 in boys, ≥27.0 in girls) [12].

Dyslipidaemia was defined as the presence of one or more of the following conditions: TChol ≥200 mg/dL, LDL-chol ≥130 mg/dL, non-HDL-chol ≥145 mg/dL, HDL-chol <40 mg/dL, and TG ≥100 mg/dL in ≤9 year-old children and TG ≥130 mg/dL in 10 year-old children [10]. Dyslipidaemia values in children according to the European Atherosclerosis Society Consensus Panel [13] were also checked.

### Statistical analysis

Analyses were performed with the SPSS statistical software package version 21.0 (SPSS Inc., Chicago, IL, USA). The level of significance for acceptance was *P* <0.05. Significant differences in prevalence were calculated by means of Kendall’s tau or χ^2^ test. The Kolmogorov-Smirnov test was used to assess if data had a normal distribution. Because most anthropometric and biochemical characteristics were not normally distributed, the median and interquartile range were given. Differences between group means were tested by Mann-Whitney *U* test and Kruskal-Wallis test. Multivariate analyses (multiple logistic regressions considering the effect of each explanatory variable adjusted for gender and age, and additionally adjusted for BMI) were used to assess the association between ALT levels (≥P_75_ *vs*. <P_75_ (reference)) and AST/ALT ratio categories (tertile 1 (T1, reference) *vs*. T2 *vs*. T3) (independent variables), and dyslipidaemia components (dependent variables).

## Results

The characteristics of the study population by ALT elevation (defined as ≥P_75_) are showed in Table 2. Children with elevated ALT had higher TG, RLP-chol, AST, and γGT levels, and low HDL-chol levels and AST/ALT ratio compared to children with ALT <P_75_. Children with high ALT showed higher prevalence of dyslipidaemia than their counterparts. The prevalence of high TChol (*P* = 0.053), non-HDL-chol, TG, and low HDL-chol were high among children with high ALT. Overall, 8 children had a LDL-chol ≥160 mg/dL and 5 of them were in the ALT ≥P_75_ group (*p*<0.038); moreover, 2 children had a LDL-chol ≥190 mg/dL (*p* = 0.472). No association between ALT levels and overweight/obesity condition and IFG were found.

**Table 2:** Characteristics of children with ALT above or below the P_75_^1^^,^^2^.

Variables	*n*	ALT < P_75_ (*n* = 301)	ALT ≥ P_75_ (*n* = 113)	*P*
Age (years)†	414	7.0 (5.0–9.0)	7.0 (5.0–9.0)	0.877
Gender				
Males‡	216	158 (73.1)	58 (26.9)	0.912
Females‡	198	143 (72.2)	55 (27.8)	
Weight (kg)†	414	23.8 (18.2–30.2)	22.9 (17.8–32.1)	0.955
Height (cm)†	414	121.5 (108.8–134.3)	120.0 (17.8–32.1)	0.595
BMI (kg/m^2^)†	414	16.2 (14.7–17.7)	15.7 (14.4–19.0)	0.878
gender- and age- BMI <25‡	321	238 (74.1)	83 (25.9)	0.240
gender- and age- BMI ≥25‡	93	63 (67.7)	30 (32.3)	
IFG (mg/dL)†	414	85.0 (79.0–90.0)	84.0 (75.0–89.5)	0.178
<100‡	389	287 (73.8)	102 (26.2)	0.096
≥ 100‡	25	14 (56.0)	11 (44.0)	
TChol (mg/dL)†	414	164.0 (146.0–183.0)	170.0 (141.0–194.5)	0.256
< 200‡	348	260 (74.7)	88 (25.3)	0.053
≥ 200‡	66	41 (62.1)	25 (37.9)	
LDL-chol (mg/dL)†	414	95.0 (79.0–112.0)	98.0 (78.0–120.0)	0.327
< 130‡	366	272 (74.3)	94 (25.7)	0.067
≥ 130‡	48	29 (60.4)	19 (39.6)	
Non-HDL-chol (mg/dL)†	414	114.0 (96.0–136.5)	120.0 (100.0–149.0)	0.060
< 145‡	322	242 (75.2)	80 (24.8)	0.049
≥ 145‡	92	59 (64.1)	33 (35.9)	
HDL-chol (mg/dL)†	414	47.0 (40.5–55.0)	41.0 (36.0–51.5)	0.002
> 40‡	305	234 (76.7)	71 (23.3)	0.004
≤ 40‡	109	67 (61.5)	42 (38.5)	
TG (mg/dL)†*	414	82.0 (66.0–109.0)	108.0 (77.0–151.5)	<0.001
< 100/130‡	266	213 (80.1)	53 (19.9)	<0.001
≥ 100/130‡	148	88 (59.5)	60 (40.5)	
RLP-chol (mg/dL)†§	414	17.0 (13.0–23.0)	22.0 (15.0–31.5)	<0.001
< 24.8/27.0‡	309	239 (77.3)	70 (22.7)	0.001
≥ 24.8/27.0‡	105	62 (59.0)	43 (41.0)	
No. dyslipidaemia components	414			
< 1‡	194	157 (80.9)	37 (19.1)	<0.001
≥ 1‡	220	144 (65.5)	76 (34.5)	
ALT (IU/L)†	414	7.0 (4.0–10.0)	17.0 (14.0–21.0)	<0.001
AST (IU/L)†	414	14.0 (11.0–18.0)	21.0 (16.0–26.5)	<0.001
AST/ALT†	414	2.20 (1.50–3.00)	1.14 (0.88–1.43)	<0.001
γGT (IU/L)†	414	10.0 (8.0–12.0)	12.0 (9.0–18.0)	<0.001

Abbreviations: ALT, alanine aminotransferase; BMI, body mass index; IFG. Impaired fasting glucose; TChol, total cholesterol; LDL-chol, low-density lipoprotein cholesterol; HDL-chol, high-density lipoprotein cholesterol; non-HDL-chol, non-high-density lipoprotein cholesterol; TG, tryglicerides; RLP-chol, remnant-like particle cholesterol; AST, aspartate aminotransferase; γGT, gamma glutamyl transferase.

^1^ Values are †median (interquartile range) and ‡*n* (%).

^2^ Significant differences between ALT groups by †Mann-Whitney *U* test and by the ‡Kendall’s tau or χ^2^ test.

* TG levels were categorised as <100 or ≥100 in children aged ≤9 years, and as <130 or ≥130 in children aged 10 years.

§ High RLP-chol levels were defined as ≥P_75_ for boys (24.8 mg/dL) and girls (27.0 mg/dL) separately.

**Fig 1:**
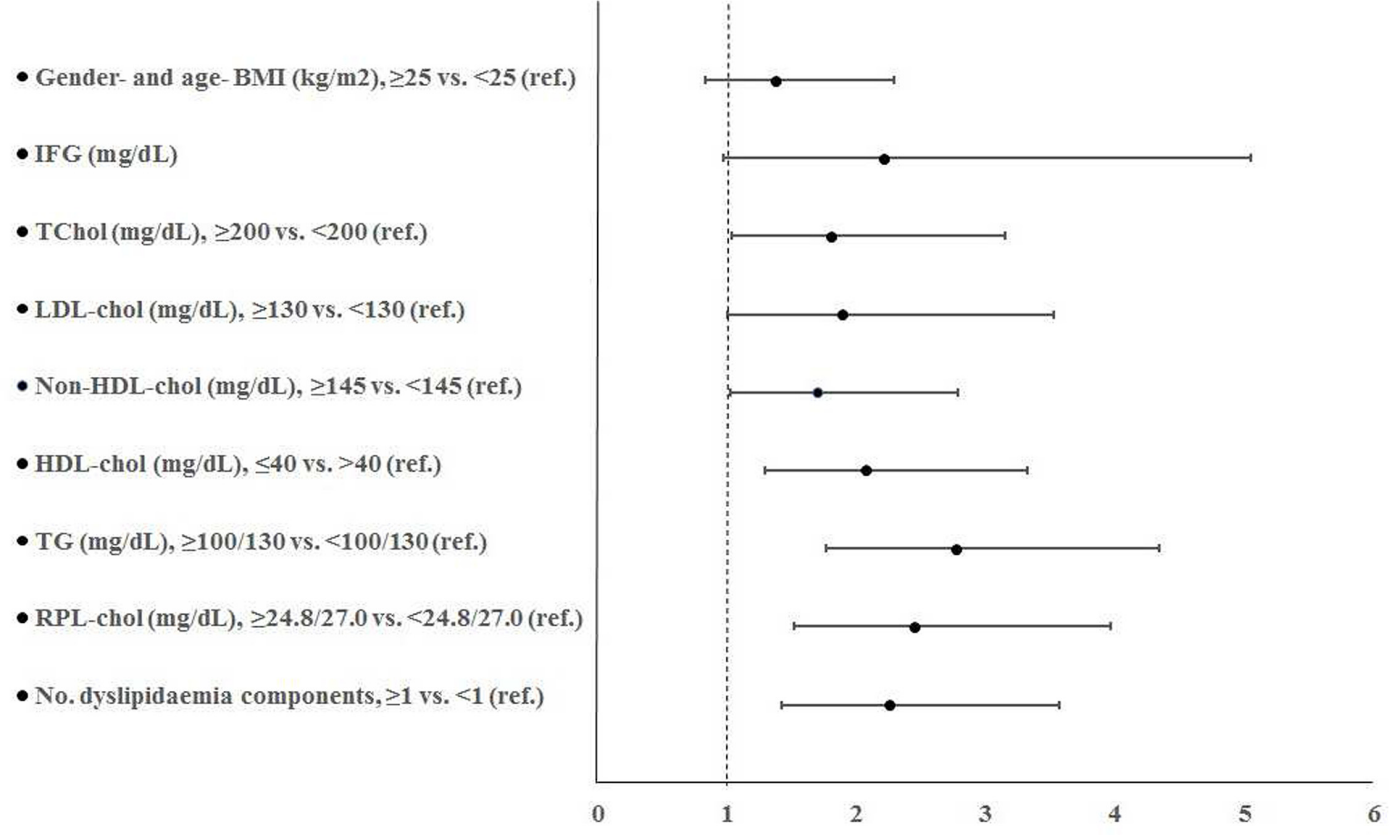
Association between BMI, serum lipid levels and dyslipidaemia and the probability of ALT above the P_75_. Logistic regression analysis (odds ratio, 95% confidence interval) considering the effect of one explanatory variable after adjustment for childrens’ gender and age (continuous variable). Abbreviations: BMI, body mass index; IFG, impaired fasting glucose; TChol, total cholesterol; LDL-chol, low-density lipoprotein cholesterol; HDL-chol, high-density lipoprotein cholesterol; non-HDL-chol, non-high-density lipoprotein cholesterol; TG, tryglicerides; RLP-chol, remnant-like particle cholesterol. †TG levels were categorised as <100 (normal) or ≥100 in children aged ≤9 years, and as <130 or ≥130 in children aged 10 years. ‡High RLP-chol levels were defined as ≥P_75_ for boys (24.8 mg/dL) and girls (27.0 mg/dL) separately.

While no association between ALT levels and overweight/obesity condition was found, logistic regression analyses showed a strong association between dyslipidaemia and ALT equal or above the P_75_ (OR: 2.26, 95% CI: 1.43–3.57; *P*<0.001) (Fig 1). High TChol, LDL-chol, non-HDL-chol, RLP-chol and TG, and low HDL-chol levels increased up to OR = 1.81-, 1.89-, 1.70-, 2.45-, 2.77-, and 2.08-fold, respectively, in all subjects after adjusting for gender and age. In multivariate logistic regression analyses, after additionally adjusting for BMI, these associations remained significant (except for LDL-chol and non-HDL-chol) (data not shown).

Overweight/obese children had a higher prevalence of AST/ALT <T1 ratio compared with their normal weight counterparts (52.7% and 32.1%, respectively) (Table 3). Differences in TChol, non-HDL-chol, HDL-chol, TG, RLP-chol, ALT, AST and γGT across AST/ALT ratio categories were found. The prevalence of high TChol, LDL-chol, non-HDL-chol, RLP-chol and TG, and low HDL-chol were statistically significantly different across AST/ALT ratio groups. While half of dyslipidaemic children (48.6%) were classified into the AST/ALT <T1 ratio group, half of non-dyslipidaemic children (46.9%) were classified into the AST/ALT ≥T3 ratio group.

**Table 3:** Characteristics of study population by different levels of AST/ALT^1^^,^^2^.

		AST/ALT ratio			
	*n*	T1 (*n* = 152)	T2 (*n* = 108)	T3 (*n* = 154)	*P*
Age (years)†	414	7.0 (5.0–9.0)	6.5 (4.0–9.0)	6.0 (5.0–9.0)	0.222
Gender					
Boys	216	87 (40.3)	54 (25.0)	75 (34.7)	0.285
Girls	198	65 (32.8)	54 (27.3)	79 (39.9)	
Weight (kg)†	414	24.4 (18.5–34.5)	23.0 (18.3–29.6)	23.0 (17.3–29.9)	0.056
Height (cm)†	414	123.8 (110.0–136.0)	121.5 (105.0–133.0)	120.0 (108.9–133.5)	0.302
BMI (kg/m^2^)†	414	16.1 (14.7–19.9)	16.0 (14.8–17.6)	15.9 (14.2–17.1)	0.096
gender- and age- BMI <25‡	321	103 (32.1)	90 (28.0)	128 (39.9)	0.002
gender- and age- BMI ≥25‡	93	49 (52.7)	18 (19.4)	26 (28.0)	
IFG (mg/dL)†	414	85.0 (80.0–91.0)	82.0 (73.0–87.8)	85.5 (79.0–90.0)	0.004
<100‡	389	139 (35.7)	103 (26.5)	147 (37.8)	0.157
≥ 100‡	25	13 (52.0)	5 (20.0)	7 (28.0)	
TChol (mg/dL)†	414	171.0 (147.0–197.0)	162.0 (141.3–180.8)	161.0 (146.0–179.3)	0.019
< 200‡	348	118 (33.9)	92 (26.4)	138 (39.7)	0.004
≥ 200‡	66	34 (51.5)	16 (24.2)	16 (24.2)	
LDL-chol (mg/dL)†	414	99.5 (78.0–121.0)	94.0 (78.3–106.8)	94.0 (79.0–111.0)	0.129
< 130‡	366	123 (33.6)	98 (26.8)	145 (39.6)	<0.001
≥ 130‡	48	29 (60.4)	10 (20.8)	9 (18.8)	
Non-HDL-chol (mg/dL)†	414	121.5 (103.0–152.0)	111.0 (94.0–132.8)	113.5 (93.8–133.0)	0.001
< 145‡	322	101 (31.4)	89 (27.6)	132 (41.0)	<0.001
≥ 145‡	92	51 (55.4)	19 (20.7)	22 (23.9)	
HDL-chol (mg/dL)†	414	42.0 (37.0–51.0)	48.0 (42.0–56.0)	47.0 (41.0–55.0)	<0.001
> 40‡	305	96 (31.5)	86 (28.2)	123 (40.3)	0.001
≤ 40‡	109	56 (51.4)	22 (20.2)	31 (28.4)	
TG (mg/dL)†*	414	108.0 (77.0–153.0)	79.5 (64.0–113.8)	77.5 (64.8–97.3)	<0.001
< 100/130‡	266	73 (27.4)	71 (26.7)	122 (45.9)	<0.001
≥ 100/130‡	148	79 (53.4)	37 (25.0)	32 (21.6)	
RLP-chol (mg/dL)†§	414	23.0 (16.0–34.5)	16.0 (12.0–23.8)	16.0 (13.0–20.0)	<0.001
< 24.8/27.0‡	309	87 (28.2)	89 (28.8)	133 (43.0)	<0.001
≥ 24.8/27.0‡	105	65 (61.9)	19 (18.1)	21 (20.0)	
No. dyslipidaemia components	414				
< 1‡	194	45 (23.2)	58 (29.9)	91 (46.9)	<0.001
≥ 1‡	220	107 (48.6)	50 (22.7)	63 (28.6)	
ALT (U/L)†	414	14.0 (10.0–18.0)	10.0 (7.0–12.0)	4.0 (3.0–7.0)	<0.001
AST (U/L)†	414	15.0 (11.0–19.0)	18.0 (14.0–21.0)	15.0 (12.0–19.0)	0.005
γGT (U/L)†	414	12.0 (9.0–18.0)	10.0 (8.0–12.0)	9.0 (8.0–12.0)	<0.001

Abbreviations: ALT, alanine aminotransferase; AST, aspartate aminotransferase; BMI, body mass index; IFG, impaired fasting glucose; TChol, total cholesterol; LDL-chol, low-density lipoprotein cholesterol; HDL-chol, high-density lipoprotein cholesterol; non-HDL-chol, non-high-density lipoprotein cholesterol; TG, tryglicerides; RLP-chol, remnant-like particle cholesterol; γGT, gamma glutamyl transferase.

^1^ Values are †median (interquartile range) and ‡*n* (%).

^2^ Significant differences between ALT groups by †Kruskal-Wallis test and by the ‡Kendall’s tau or χ^2^ test.

* TG levels were categorised as <100 or ≥100 in children aged ≤9 years, and as <130 or ≥130 in children aged 10 years.

§ High RLP-chol levels were defined as ≥P_75_ for boys (24.8 mg/dL) and girls (27.0 mg/dL) separately.

Compared with subjects with an AST/ALT <T1 ratio, subjects with an AST/ALT ≥T3 ratio were significantly less likely to be overweight/obese (OR: 0.43, 95% CI: 0.25–0.74) (Table 4). Logistic regression analyses also showed a strong association between dyslipidaemia and AST/ALT ratio. Subjects with an AST/ALT ≥T3 ratio were 0.27-times (95% CI: 0.17–0.44) less likely to have dyslipidaemia than those with an AST/ALT <T1 ratio. The risk of high TChol, LDL-chol, non-HDL-chol and TG, and low HDL-chol levels decreased across AST/ALT ratio groups after adjusting for gender and age. High TChol, LDL-chol, non-HDL-chol and TG, and low HDL-chol levels decreased up to 0.40-, 0.25-, 0.33-, 0.23-, and 0.41-fold, respectively, in all subjects with an AST/ALT ≥T3 ratio, compared with those with an AST/ALT <T1 ratio. In multivariate logistic regression analyses, after additionally adjusting for BMI, these associations remained significant. Contrarily, no statistical significant association between AST/ALT ratio categories and IFG was obtained.

**Table 4:** Association between BMI, serum lipid levels and dyslipidaemia and the probability of different levels of AST/ALT ratio^1^^,^^2^^,^^3^.

	AST/ALT ratio			
	T1 (*n* = 152)	T2 (*n* = 108)	T3 (*n* = 154)	*P*
Gender- and age- BMI (kg/m^2^), ≥25 *vs*. <25 (ref.)				
^1^Gender- and age-adjusted	1.00 (ref.)	0.44 (0.24–0.81)**	0.43 (0.25–0.74)**	0.003
^2^Additionally adjusted	―	―	―	―
IFG (mg/dL), ≥100 *vs*. <100 (ref.)				
^1^Gender- and age-adjusted	1.00 (ref.)	0.53 (0.18–1.55)^ns^	0.51 (0.20–1.33)^ns^	0.294
^2^Additionally adjusted	1.00 (ref.)	0.61 (0.21–1.83)^ns^	0.59 (0.22–1.57)^ns^	0.494
TChol (mg/dL), ≥200 *vs*. <200 (ref.)				
^1^Gender- and age-adjusted	1.00 (ref.)	0.61 (0.31–1.17)^ns^	0.40 (0.21–0.76)**	0.017
^2^Additionally adjusted	1.00 (ref.)	0.60 (0.31–1.17)^ns^	0.39 (0.20–0.75)**	0.017
LDL-chol (mg/dL), ≥130 *vs*. <130 (ref.)				
^1^Gender- and age-adjusted	1.00 (ref.)	0.41 (0.19–0.88)*	0.25 (0.11–0.55)**	0.001
^2^Additionally adjusted	1.00 (ref.)	0.42 (0.19–0.91)*	0.26 (0.12–0.57)**	0.002
Non-HDL-chol (mg/dL), ≥145 *vs*. <145 (ref.)				
^1^Gender- and age-adjusted	1.00 (ref.)	0.43 (0.23–0.78)**	0.33 (0.19–0.58)***	<0.001
^2^Additionally adjusted	1.00 (ref.)	0.46 (0.25–0.85)*	0.36 (0.20–0.63)***	0.001
HDL-chol (mg/dL), ≤40 *vs*. >40 (ref.)				
^1^Gender- and age-adjusted	1.00 (ref.)	0.42 (0.23–0.74)**	0.41 (0.24–0.68)**	0.001
^2^Additionally adjusted	1.00 (ref.)	0.48 (0.26–0.86)*	0.47 (0.27–0.80)**	0.007
TG (mg/dL), ≥100/130 *vs*. <100/130 (ref.)^3^				
^1^Gender- and age-adjusted	1.00 (ref.)	0.46 (0.28–0.78)**	0.23 (0.14–0.38)***	<0.001
^2^Additionally adjusted	1.00 (ref.)	0.51 (0.30–0.87)*	0.25 (0.15–0.42)***	<0.001
RPL-chol (mg/dL), ≥24.8/27.0 *vs*. <24.8/27.0 (ref.)^4^				
^1^Gender- and age-adjusted	1.00 (ref.)	0.29 (0.16–0.54)***	0.21 (0.12–0.37)***	<0.001
^2^Additionally adjusted	1.00 (ref.)	0.33 (0.18–0.60)***	0.23 (0.13–0.41)***	<0.001
No. dyslipidaemia components, ≥1 *vs*. <1 (ref.)				
^1^Gender- and age-adjusted	1.00 (ref.)	0.35 (0.21–0.59)***	0.27 (0.17–0.44)***	<0.001
^2^Additionally adjusted	1.00 (ref.)	0.37 (0.22–0.63)***	0.29 (0.18–0.48)***	<0.001

Abbreviations: OR, odds ratio; CI, confidence interval; BMI, body mass index; IFG, impaired fasting glucose; TChol, total cholesterol; LDL-chol, low-density lipoprotein cholesterol; HDL-chol, high-density lipoprotein cholesterol; non-HDL-chol, non-high-density lipoprotein cholesterol; TG, tryglicerides; RLP-chol, remnant-like particle cholesterol.

^1,2^ Logistic regression analysis considering the effect of one explanatory variable after adjustment for childrens’ ^1^gender and age (continuous variable), and ^2^BMI (continuous variable).

^3^ TG levels were categorised as <100 or ≥100 in children aged ≤9 years, and as <130 or ≥130 in children aged 10 years.

^4^ High RLP-chol levels were defined as ≥P_75_ for boys (24.8 mg/dL) and girls (27.0 mg/dL) separately.

* *P*<0.05

** *P*<0.01

*** *P*<0.001

ns: not significant.

## Discussion

The most interesting findings of this study are that children with an AST/ALT ≥T3 ratio were less times likely to be overweight/obese and to have dyslipidaemia than those with an AST/ALT <T1 ratio, as well as the risk of high TChol, LDL-chol, non-HDL-chol and TG, and low HDL-chol levels decreased across AST/ALT ratio groups. Children with high ALT also showed higher prevalence of dyslipidaemia than their counterparts.

### Liver enzymes and body weight

In the present study, no association between ALT levels with BMI and overweight/obesity condition was found. Contrarily, previous child and adolescent studies showed elevated serum ALT levels were associated with higher BMI [14,15], fat mass index [15] and WC [14–16], and higher prevalence of central obesity [5,14], body fat proportion [5], and overweight and/or obesity [5,6,14,17] compared to subjects with serum ALT levels within the healthy range. Therefore, most cases of elevated ALT can be attributed to being overweight and obese [4].

An AST/ALT <1 ratio is characteristic of acute viral hepatitis (e.g. hepatitis A and E), and also chronic viral hepatitis (e.g. hepatitis B and C) in spite of the fact that this particularly occurs when progression to fibrosis and cirrhosis is present [4]. In fact, hepatitis A is the most prevalent viral hepatitis infection detected in Mexican children [18]. However, in accordance with our results, AST/ALT ratio has been inversely associated with BMI, fat mass index, and WC in adolescents from the HELENA study. AST/ALT was also lower in overweight and obese adolescents than in their leaner peers [15].

### Liver enzymes and dyslipidaemia

In accordance with previous paediatric studies, elevated serum ALT levels were associated with high TG levels [14,16], high LDL-chol levels [14], high non-HDL-chol [19], and low HDL-chol levels [14]. High serum ALT levels were also associated with MetS [5,14,17]. In this study, dyslipidaemia was 2.24-fold higher (95% CI: 1.40–3.56) in children with an ALT equal or above the P_75_ compared to those with an ALT under the P_75_, and 0.29-fold lower in children with an AST/ALT ≥T3 ratio compared to those with an AST/ALT <1 ratio, after adjusting for age, gender and BMI. In the HELENA study, higher ALT and γGT levels and lower AST/ALT were also significantly associated with higher values of cardiometabolic risks, however, with significant differences by gender in the association of ALT and AST/ALT with blood pressure, insulin resistance and blood lipids. Nevertheless, receiver operating characteristic (ROC) analysis showed a significant discriminating accuracy of AST/ALT in identifying a low or high cardiometabolic risk in both males and females, supporting the idea that the AST/ALT may be especially useful as a screening test in the clinical evaluation of adolescents with high cardiovascular risk factors [15].

In accordance with our results, the ATTICA study (a health and nutrition survey that is being carried out in the Greek province of Attica) revealed that a 1 unit increase in the AST/ALT ratio was associated with a 0.37-times (95% CI: 0.16–0.84) lower likelihood of having MetS in males (18–87 years old) and with a 0.48-times (95%CI: 0.28–0.86) lower likelihood in females (18–89 years old) [20]. Moreover, only in subjects with moderate to low adherence to the Mediterranean diet, an increase in the AST/ALT ratio was associated with a lower likelihood of having MetS; however, the AST/ALT ratio was not associated with the presence of MetS in those with greater adherence to the Mediterranean diet [20]. Although it is known that a high AST/ALT ratio may be indicative of severe liver damage from alcohol, it is not the case in our study as the ratio of aminotransferases is not combined with elevated liver enzymes [20]. The proportion of carbohydrate in energy intake, but not fat, has also been positively correlated with abnormal aminotransferase activity in adults [21,22].

Finally, in familial combined hyperlipidaemia subjects, HOMA-index has been strongly associated with liver steatosis [23]. In the present study, no association between ALT levels and AST/ALT ratio with IFG were found, however, Fracanzani *et al*. [24] previously reported a significant association between diabetes with severe liver disease in adult patients with NAFLD with elevated ALT, but also HOMA-IR in patients with normal ALT, in spite that Manco et al. [25] stated that prevalence of MetS is similar between patients with normal and abnormal ALT values, and that normal ALT values cannot exclude evidence of liver steatosis.

## Strengths and Limitations

This study has several strengths. Firstly, it is often difficult to obtain a fasting lipid profile in large population surveys [26,27] and even more so in children. Secondly, the large, diverse and complex evidence base that addresses cardiovascular disease risk beginning in childhood, and the absence of decades-long event-driven clinical trials, requires consideration of substantial and consistent evidence from observational studies, developing a chain of evidence [28].

This study also has several limitations. Firstly, definitive diagnosis requires a liver biopsy, but this is not feasible in large-scale epidemiological studies and clinical fields. Moreover, we were not able to exclude that liver enzyme concentrations could have been affected by viral hepatitis or drug-induced liver injury. Secondly, apoB and apoA-1 levels were not measured. However, most, but not all, studies point out that measurement of apoB and apoA-1 for universal screening provides no additional advantages over measuring non-HDL-chol, LDL-chol and HDL-chol [29]. Thirdly, although Metabolic Syndrome is frequently characterized by high levels of liver enzymes, we couldn’t evaluate the presence of Metabolic Syndrome in our children population. Moreover, no information about the family history of NAFLD and Familial hypercholesterolaemia (FH) were assessed. Childhood is the optimal period for discrimination between FH and non-FH using LDL-chol screening. In accordance, 2 children had a LDL-chol ≥190 mg/dL, however, no information about family history of premature coronary heart disease and cholesterol levels of both parents were assessed to make the phenotypic diagnosis [13]. Fourthly, the guidelines do not account for variations in lipids due to race, gender, puberty status [30] and geographic location [11], all of which are known to have a clinically relevant impact on lipoprotein values. Fifthly, the cross-sectional design of the survey did not allow us to evaluate the causal relations between ALT and AST/ALT levels and dyslipidaemia. A longitudinal study on the paediatric population is needed to clarify this issue.

## Conclusions

Higher ALT and lower AST/ALT are associated with higher high TChol, LDL-chol, non-HDL-chol and TG levels and dyslipidaemia, and lower HDL-chol levels; whereas children with higher AST/ALT were more likely to present lower high TChol, LDL-chol, non-HDL-chol and TG levels and dyslipidaemia, and lower low HDL-chol compared with those with lower AST/ALT. Our results pose the need for further investigation on whether AST/ALT may be useful as a screening test in the assessment of children with cardiometabolic risk.

## Supporting Information

S1 Tables State Survey of Nutrition and Health–Nuevo León 2011/2012 (EESN-NL 2011/2012). (PDF)
