# Supplementary material for: Relation between Liver Transaminases and Dyslipidaemia among 2-10 y.o. Northern Mexican Children
Source: PLoS One. 2016 May 20;11(5):e0155994. doi: 10.1371/journal.pone.0155994 (PMC4874541; doi:10.1371/journal.pone.0155994)
Supplement: S1 Tables — (PDF) [file pone.0155994.s001.pdf]

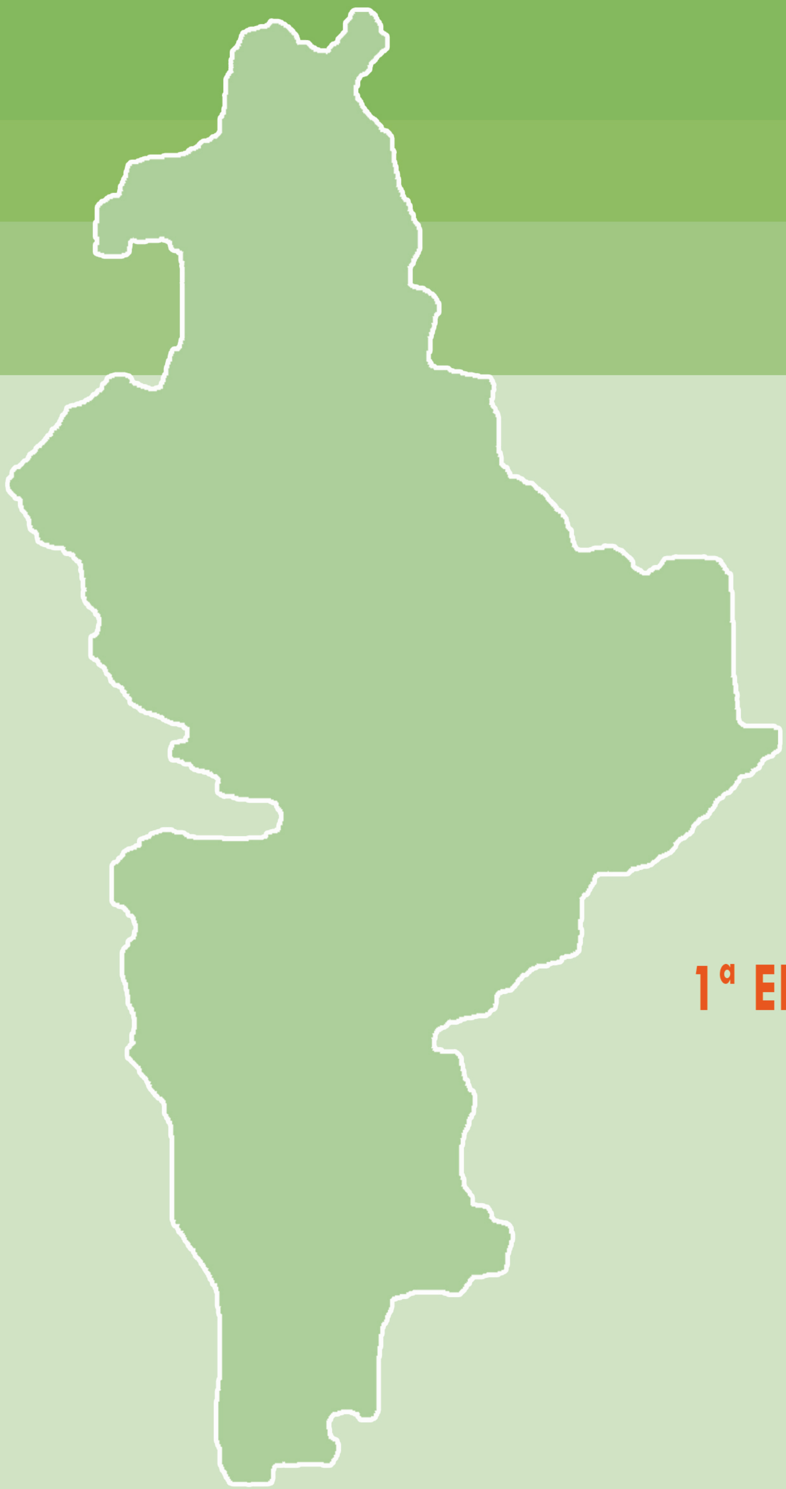

RESULTADOS POR REGIÓN
1ª ENCUESTA ESTATAL DE SALUD Y NUTRICIÓN-NUEVO LEÓN
2011 / 2012

Nuevo León Unido
Gobierno para Todos
Secretaría de SALUD NL
Secretaría de EDUCACIÓN NL
DIF NUEVO LEÓN
UANL UNIVERSIDAD AUTÓNOMA DE NUEVO LEÓN
FaSPyN U A N L
cáritas de monterrey
BANCO DE ALIMENTOS CARITAS DE MONTERREY

Gobierno del Estado de Nuevo León

C. Lic. Rodrigo Medina de la Cruz
Gobernador Constitucional del Estado de Nuevo León

Secretaría de Salud de Nuevo León

Dr. Jesús Zacarías Villarreal Pérez
Secretario

Dr. Francisco González Alanís
Subsecretario de Prevención y Control de Enfermedades

Sistema para el Desarrollo Integral de la Familia del Estado de Nuevo León

Lic. Gretta Salinas de Medina
Presidenta del Patronato

CP. José Ramón Carrales Batres
Director General

Lic. Ana Laura Martínez Rodríguez
Directora de Asistencia Social

Universidad Autónoma de Nuevo León

Dr. Jesús Áncer Rodríguez
Rector

MSP. Hilda Irene Novelo Huerta
Directora de la Facultad de Salud Pública y Nutrición

Dr. Santos Guzmán López
Director de la Facultad de Medicina y del Hospital Universitario "Dr. José Eleuterio González"

Cáritas de Monterrey, ABP

Ing. Marcelo Canales Clariond
Presidente del Patronato

Dr. Oscar Cuauhtémoc Reyes Martínez
Director General

Instituto Nacional de Estadística y Geografía

Lic. Alejandra Cervantes Martínez
Coordinadora Estatal de Nuevo León

Coordinación editorial:

Dr. en C. Rogelio Salas García
MSP. Lourdes Huerta Pérez
Dr. Jesús Zacarías Villarreal Pérez
MSP. Yolanda Elva de la Garza Casas

Edición:

Dr. en C. Rogelio Salas García
MSP. Lourdes Huerta Pérez
Dr. en C. Erik Ramírez López
Dr. en CS. Esteban Gilberto Ramos Peña
MSP. Blanca Cecilia Castillo Treviño
LN. Norma Leticia Botello Rodríguez
LN. María Luisa Saldaña Chávez
Dr. Fernando Javier Lavalle González
PhD. Elizabeth Solís Pérez
MSP. Yolanda Elva de la Garza Casas
MCSP. Shunashii Rogelly Silva Hurtado
Ing. Laura Otero Bautista

Coordinación de diseño e imagen:

Prof. Bruno Martín Jacobo Montemayor

Corrector de estilo:

Mtro. Humberto Salazar Herrera

ENCUESTA ESTATAL DE SALUD Y NUTRICIÓN-NUEVO LEÓN 2011/2012 (EESN-NL 2011/2012)
Primera edición, noviembre de 2013.

D.R. 2012 Secretaría de Salud del Estado de Nuevo León.
Impreso y hecho en México.

Comité Técnico Operativo de la Encuesta Estatal de Salud y Nutrición – Nuevo León 2011/2012

Secretaría de Salud de Nuevo León

Dr. Jesús Zacarías Villarreal Pérez
Secretario

Dr. Francisco González Alanís
Subsecretario de Prevención y Control de Enfermedades

MSP. Lourdes Huerta Pérez
Directora de Promoción de la Salud

LN. María Luisa Saldaña Chávez
Coordinadora Estatal de Nutrición

MCSP. Shunashii Rogelly Silva Hurtado
Coordinadora de Programa Estratégico

Sistema para el Desarrollo Integral de la Familia del Estado de Nuevo León

LN. Norma Leticia Botello Rodríguez
Subdirectora de Investigación y Orientación Alimentaria

LN. Dulce Adriana Mota Macías
Jefa del Depto. de Orientación Alimentaria

Cáritas de Monterrey, ABP

MSP. Blanca Cecilia Castillo Treviño
Subdirectora de Servicios de Alimentación y Nutrición

Dra. Sanjuanita Torres García
Subdirectora de Servicios de Salud

MSP. Cecilia Briones Hernández
Jefa de Área de Nutrición Rural

Secretaría de Educación

PhD. Elizabeth Solís Pérez
Coordinadora General Estratégica del programa Salud para Aprender

Universidad Autónoma de Nuevo León
Facultad de Salud Pública y Nutrición

Dr. en CS. Esteban Gilberto Ramos Peña
Subdirector de Investigación, Innovación y Posgrado

Dr. en C. Rogelio Salas García
Responsable del Laboratorio de Nutrición Poblacional del Centro de Investigación en Nutrición y Salud Pública

Dr. en C. Erik Ramírez López
Responsable del Laboratorio de Composición Corporal del Centro de Investigación en Nutrición y Salud Pública

Facultad de Medicina y Hospital Universitario “Dr. José Eleuterio González”

Dr. Fernando Javier Lavalle González
Profesor y Jefe de la Clínica de Diabetes del Depto. de Endocrinología

Instituto Nacional de Estadística y Geografía

Lic. José Francisco Sánchez Gutiérrez
Jefe del Departamento de Desarrollo Estadístico

Autores por capítulo

Capítulo 1. Perfil Sociodemográfico

MSP. Lourdes Huerta Pérez
Dr. en C. Rogelio Salas García
LN. Miriam Elizabeth de la Rosa Gil

Capítulo 2. Salud

Dr. en C. Rogelio Salas García
MSP. Lourdes Huerta Pérez
Dr. en CS. Esteban Gilberto Ramos Peña
MSP. Blanca Castillo Quezada
MSP. Cecilia Briones Hernández
MCSP. Shunashii Rogelly Silva Hurtado
Dr. Gerardo Garza Sepúlveda
MSP. María Teresa Ramos Cavazos
MSP. Leticia María Hernández Arizpe
MSP. Yolanda Elva de la Garza Casas
MC. María Adela Martínez Álvarez
MSP. Ana Alicia Alvídrez Morales
Dra. en SP. Ana María Salinas Martínez
LN. Norma Leticia Botello Rodríguez
LN. María Luisa Saldaña Chávez
MSP. Ana María Villarreal Herrera
Lic. Ernesto Moreno Sifuentes

Capítulo 3. Estado Nutricio

Dr. en C. Erik Ramírez López
MSP. Blanca Castillo Quezada
MSP. Cecilia Briones Hernández
Dra. en Med. Georgina Mayela Núñez Rocha
ENC. María del Carmen Mata Obregón
LN. Norma Leticia Botello Rodríguez
LN. Dulce Adriana Mota Macías
PhD. Elizabeth Solís Pérez
MSP. Hilda Irene Novelo Huerta

Capítulo 4. Alimentación

Dr. en CS. Esteban Gilberto Ramos Peña
Dr. en C. Rogelio Salas García
MCSP. Shunashii Rogelly Silva Hurtado
MSP. Magdalena Soledad Chavero Torres, NC.

Capítulo 5. Servicios de Salud

LN. Norma Leticia Botello Rodríguez
Dr. en C. Pedro César Cantú Martínez
LN. Dulce Adriana Mota Macías
MSP. Adriana Zambrano Moreno

Equipo Auxiliar

L.N. Adriana Ahidé García Esquivel [1]

MSP. Adriana Zambrano Moreno, NC.. [2]

ENC. Alma Angélica Trujillo Rodríguez [1]

MSP. Alpha Berenice Medellín Guerrero [2]

Est. Arely Sarahy Gutiérrez Lara [2]

MSP. David Moreno García [2]

QBP. Edgar Iván Galindo Galindo [1]

Dr. en C. Eduardo Campos Góngora [2]

ME. Guillermina Juárez Villalobos [2]

LN. José Daniel Hidrogo Salinas [2]

LN. Karen Guadalupe Contreras Ovando [2]

LN. Laura Melissa Garza Sáenz [1,2]

MC. Lidia Elena Molina Rountree [2]

MSP. Liliana Zandra Tijerina González [2]

MSP. Luz Natalia Berrún Castañón, NC. [2]

MSP. Magdalena Soledad Chavero Torres, NC. [2]

1 Secretaría de Salud
2 Facultad de Salud Pública y Nutrición, UANL
3 Cáritas de Monterrey, ABP

LN. Marco Iván Cantú Juárez [2]

LN. María Alejandra Villarreal Pérez [2]

Dra. en C. María de Jesús Ibarra Salas [2]

MSP. María de los Ángeles García Garza [2]

ENC. María del Carmen Mata Obregón [2]

LN. María Elena Trujillo Manzanares [2]

MSP. Nohemí Liliana Negrete López [2]

MTICE. Norma Angélica Pérez Ángeles [2]

Dr. Óscar Cuauhtémoc Reyes Martínez [3]

MC. Rosalía Reyes Sánchez [2]

Dra. Verónica López Guevara [2]

Est. Verónica Valles Ballesteros [2]

LN. Victoria Estefanía Peña Villarreal [2]

LN. Yenisei Ramírez Toscano [2]

Dr. en C. Zacarías Jiménez Salas [2]

Captura de la información

Centro de Evaluaciones de la UANL

Laboratorio de Nutrición Poblacional, FaSPyN, UANL.

Procesamiento de la base de datos

Ing. Nahúm Espinoza Moreno

MEI. Enrique Martínez Alvarado

MEI. Oscar de la Garza Garza

MTICE. Norma Angélica Pérez Ángeles

Auditoría de la base de datos

MEI. Enrique Martínez Alvarado

MEI. Oscar de la Garza Garza

Equipo de Profesionales de la Salud (Encuestadores)

Ver anexo 1

Equipo de validación de la base de datos [2]

Ver anexo 2

Citación sugerida:

Secretaría de Salud de Nuevo León / Universidad Autónoma de Nuevo León / Facultad de Salud Pública y Nutrición / Facultad de Medicina / Sistema para el Desarrollo Integral de la Familia del Estado de Nuevo León / Cáritas de Monterrey, ABP / Secretaría de Educación de Nuevo León. Encuesta Estatal de Salud y Nutrición – Nuevo León 2011/2012. Monterrey, N. L. México, 2012.

Índice

**Mensaje**	11
**Presentación**	13
**Metodología**	15
Diseño de la muestra	17
Muestreo	19
Tamaño de la muestra	22
Selección de la muestra	24
Obtención de la información	26
Procesamiento de muestras de sangre	30
Captura de la información	31
Auditoría de la base de datos	31
Procesamiento de la base de datos	32
Consideraciones éticas	32
**Perfil Sociodemográfico**	33
Características de la población	36
Características del hogar y la vivienda	45
Bienes o activos del hogar	49
Resultados relevantes	52
**Salud**	53
Enfermedad diarreica aguda	55
Infecciones respiratorias	57
Lactancia materna	58
Vacunación	59
Actividad física	61
Diabetes	65
Hipertensión	67
Depresión	71
Evaluación cognitiva y física	72
Cáncer	74
Salud reproductiva	75
Daños a la salud por accidentes	83
Factores de riesgo (tabaquismo y alcohol)	85
Resultados relevantes	87

**Estado Nutricio**	**91**
Metodología	**95**
Menores de 5 años	**99**
Niños de 5 a 11 años	**101**
Adolescentes	**104**
Adultos	**105**
Adultos mayores	**109**
Resultados relevantes	**111**
**Alimentación**	**115**
Alimentación en los diferentes grupos de edad	**117**
Consumo de alimentos en Nuevo León	**118**
Consumo de alimentos en los diferentes grupos de edad en Nuevo León	**119**
Resultados relevantes	**126**
**Servicios de Salud**	**129**
Patrones de utilización de servicios de salud	**132**
Gasto de utilización de servicios de salud	**140**
Programas de ayuda alimentaria	**143**
Resultados relevantes	**144**
**Conclusiones**	**145**
**Bibliografía**	**151**
**Anexos**	**153**

Mensaje del C. Gobernador del Estado de Nuevo León

Desde el inicio de mi gobierno hemos planteado como objetivo mejorar la calidad de vida de los nuevoleoneses, en especial a través de la salud; para lograrlo se hace indispensable trabajar en un marco de servicios de calidad y transparencia, a través de la implementación de políticas públicas que propicien la equidad social en las áreas de marginación urbana y rural.

Estamos fortaleciendo la salud como un potencial de los nuevoleoneses, uno de los retos es abatir el rezago; los servicios de salud tienen que estar al alcance de todos para lograr calidad de vida.
Se trabaja de manera anticipatoria para que la población siga sana, formamos una nueva cultura de la salud, estamos combatiendo los principales padecimientos que afectan la salud de los nuevoleoneses.

El impacto contundente de un servicio de calidad debe partir de un conocimiento sólido, real y científico sobre la situación de salud de nuestra población. En cumplimiento a ello es que en noviembre de 2010 apoyamos la iniciativa para que en Nuevo León se aplicara la Encuesta Estatal de Salud y Nutrición (EESN-NL 2011/2012), la primera de esta índole en una entidad federativa del país, la cual permite tener acceso a información oportuna, real, amplia sobre una diversidad de temas prioritarios para nuestro Estado, como son los perfiles de riesgos, los determinantes que condicionan los procesos de salud y enfermedad; esta información genera diagnósticos por región. Adicional a esto la Encuesta se convierte en un instrumento para medir el impacto de los programas de salud y el conocimiento de cómo vamos avanzando.

El día de hoy, con la edición del presente informe, se cumple con la segunda etapa de difusión de los principales resultados por región, los cuales han de fundamentar la rendición de cuentas y la toma de decisiones en materia de salud pública, para ser más efectivos y lograr mejores indicadores de salud, traduciéndose en una población más sana y con mayor bienestar.

Lic. Rodrigo Medina de la Cruz
Gobernador Constitucional de Nuevo León
Monterrey, Nuevo León, diciembre de 2012

Presentación

La primera Encuesta Estatal de Salud y Nutrición de Nuevo León constituye un esfuerzo sin precedente en materia de salud pública, no sólo porque que se enmarca en el contexto de la medición y evaluación del problema de la obesidad y sus enfermedades concomitantes, sino porque ha permitido contar con un diagnóstico actualizado de las condiciones de salud y nutrición de la población nuevoleonesa. Lo anterior, sin duda, es un insumo necesario para la planeación estratégica, la identificación de factores críticos de éxito en la política pública en salud y nutrición y por consecuencia para la toma de decisiones basada en evidencia científica.

El proyecto de la encuesta tuvo su origen en diciembre de 2010, en el marco del Programa de Alimentación Saludable y Actividad Física para la Prevención de la Obesidad y el Sobrepeso. Por indicaciones del Lic. Rodrigo Medina de la Cruz, Gobernador Constitucional del Estado de Nuevo León, la Secretaría de Salud convocó a un grupo de expertos de la Universidad Autónoma de Nuevo León (Facultad de Medicina y Facultad de Salud Pública y Nutrición) Cáritas de Monterrey ABP, al Sistema para el Desarrollo Integral de la Familia del Estado de Nuevo León, a la Secretaría de Educación y al Instituto Nacional de Estadística y Geografía-Delegación Nuevo León, integrándose un grupo de trabajo técnico que operó y concluyó el proyecto. Es así como, conjuntando voluntades y capacidades institucionales se llevó a cabo la "1ª Encuesta Estatal de Salud y Nutrición - Nuevo León 2011/2012" (EESN-NL 2011/2012).

En la planeación y diseño metodológico de esta investigación se contó con la asesoría y colaboración del Instituto Nacional de Salud Pública y del Instituto Nacional de Estadística y Geografía; en la fase de recolección de la información se involucró también a un equipo interdisciplinario de salud conformado por nutriólogos, enfermeros, químicos y promotores de salud. Para asegurar la confiabilidad y la representatividad de la información, los datos fueron auditados por especialistas.

El documento en extenso que aquí se presenta contiene información muy valiosa sobre los principales problemas de salud y nutrición, los factores de riesgo ambientales, socioeconómicos, culturales, los estilos de vida asociados con la salud, los programas asistenciales y la cobertura en salud en el estado de Nuevo León. Dichos datos servirán de base para medir el impacto de las acciones de promoción y atención a la salud implementadas por el Gobierno de Nuevo León.

Los resultados se describen a nivel estatal y para las cuatro regiones en las que se dividió el estado (Metropolitana, Centro, Sur y Norte) para focalizar los problemas; a su vez, la información es desplegada en cinco capítulos. El primer capítulo refiere el perfil sociodemográfico de la población, las características de los hogares, las viviendas y los individuos que en ellas habitan, los servicios básicos, la escolaridad, la actividad productiva, los bienes y activos que posee la población. El segundo capítulo abarca las condiciones de salud, la presencia de enfermedades y aspectos como

vacunación, lactancia, enfermedad diarreica, enfermedades respiratorias, actividad física, diabetes, hipertensión arterial, cáncer, depresión, salud reproductiva, accidentes, consumo de alcohol y tabaquismo en la población, considerando cuatro grupos de edad. La descripción del estado nutricio de la población se presenta en el capítulo tres, caracterizando los problemas de desnutrición, sobrepeso y obesidad en preescolares, escolares, adolescentes, adultos y adultos mayores. El tema de la alimentación se describe en el capítulo cuatro, mostrando evidencia de los principales alimentos que se consumen, la dieta y la conformación de la canasta básica de alimentos de las familias de Nuevo León. Finalmente, en el capítulo cinco se presentan datos sobre la utilización de los servicios de salud, entre estos el Seguro Popular y los programas de ayuda alimentaria.

Lo aquí expuesto es el resultado del esfuerzo de más de 200 profesionales de la salud pública que ponen de manifiesto el carácter colaborativo y la sinergia interinstitucional en el ejercicio del cumplimiento de su misión, con la convicción de que la salud es tarea de todos. A su vez, esta investigación refrenda el compromiso de generar y difundir información objetiva y confiable para la implementación de políticas públicas, basadas en evidencia científica, como lo es la Estrategia Estatal para la Prevención y el Control de la Obesidad y la Diabetes, por citar un ejemplo de su utilidad.

Es importante señalar también, que la EESN-NL 2011/2012 ha permitido consolidar una base de datos muy completa y que se pone a disposición del público. Su utilización permitirá a los profesionales e investigadores interesados, de las diferentes instituciones y organizaciones públicas y privadas, generar estudios científicos y/o diseñar programas, acciones y estrategias de atención focalizados, desde sus respectivos ámbitos de actuación.

El conocimiento de nuestra realidad en salud, ratificará la dirección estratégica y puntual de los esfuerzos públicos y privados, contribuyendo a abatir los principales problemas de salud que aquejan a la población nuevoleonesa, y a disminuir los factores de riesgo presentes en nuestra sociedad.

Unidos por la salud, se logrará más en calidad de vida para todos los nuevoleoneses.

Dr. Jesús Zacarías Villarreal Pérez
Secretario de Salud del Estado de Nuevo León

Metodología

EESN – NL 2011/2012

Metodología

La Síntesis Ejecutiva de la 1ª. Encuesta Estatal de Salud y Nutrición – Nuevo León 2011/2012 cita en el capítulo de metodología que Nuevo León es un estado federativo que se encuentra en el noreste de la Encuesta México, tiene una extensión territorial de 64,220 km^2, y colinda con los estados de Tamaulipas, Coahuila, San Luis Potosí y Zacatecas, y con el estado de Texas de los Estados Unidos de América.

Políticamente está constituido por 51 municipios. Tiene una población de 4, 653, 458 habitantes, de los cuales el 84.5% se encuentra en el área metropolitana, la cual está constituida por nueve municipios: Apodaca, García, Gral. Escobedo, Guadalupe, Juárez, Monterrey, San Nicolás de los Garza, San Pedro Garza García y Santa Catarina [INEGI, 2010]. Sus principales actividades son los servicios, el comercio y la fabricación (metal, maquinaria y equipo). El clima es caluroso y seco, con lluvias predominantemente en los meses de mayo y septiembre. El PIB asciende a 615,998 millones de pesos y el PIB *per cápita* a 154,024 pesos. Las exportaciones equivalen a 14,511 millones de dólares [Gobierno de Nuevo León, 2011].

La Encuesta Estatal de Salud y Nutrición – Nuevo León 2011/2012 (EESN-NL 2011/2012) fue diseñada para obtener información que permitiera identificar los principales indicadores de salud y nutrición de los habitantes de Nuevo León, considerando los diversos grupos de edad; para su diseño se realizó la revisión y análisis del contenido de la Encuesta Nacional de Salud y Nutrición 2006 (ENSANUT-2006).

Diseño de la muestra

El diseño muestral para la EESN-NL 2011/2012 fue desarrollado por el Instituto Nacional de Estadística y Geografía (INEGI), estableciendo cuatro regiones para la aplicación de la encuesta. Las regiones fueron denominadas Metropolitana, Centro, Sur y Norte (Figura A).

Figura A. Nuevo León de acuerdo a las regiones establecidas para la aplicación de la EESN-NL 2011/2012.

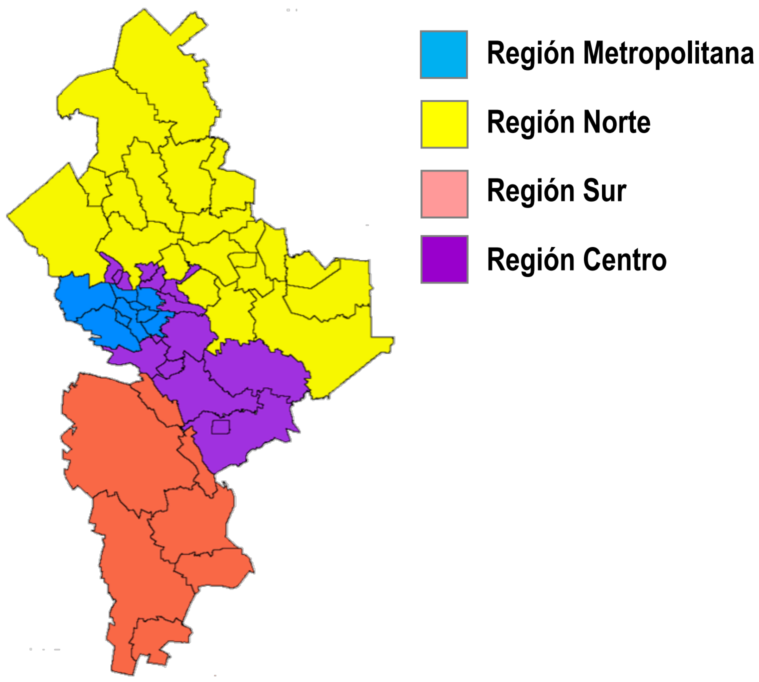

Regiones geográficas

Metropolitana	Norte	Sur	Centro
Monterrey	Mina	Rayones	El Carmen
Escobedo	Salinas Victoria	Aramberri	Ciénega de Flores
San Nicolás de los Garza	Anáhuac	Dr. Arroyo	Gral. Zuazua
Guadalupe	Bustamante	Galeana	Hidalgo
Apodaca	Lampazos de Naranjo	Gral. Zaragoza	Cadereyta Jiménez
Juárez	Sabinas Hidalgo	Mier y Noriega	Marín
Santa Catarina	Vallecillo	Iturbide	Pesquería
San Pedro Garza García	Cerralvo		Allende
García	China		Hualahuises
	Dr. Coss		Linares
	Dr. González		Montemorelos
	Gral. Bravo		Gral. Terán
	Los Ramones		Abasolo
	Los Aldama		Santiago
	Agualeguas		
	Gral. Treviño		
	Higueras		
	Los Herreras		
	Melchor Ocampo		
	Parás		
	Villaldama		

- **Población objetivo**

La encuesta estuvo dirigida a la población residente en viviendas particulares habitadas, dentro del estado de Nuevo León, diferenciada en los grupos de 0 a 9 años, 10 a 19, 20 a 59 y 60 años y más.

- **Cobertura geográfica**

La encuesta fue diseñada para dar resultados a nivel estatal y regional.

- **Marco de la encuesta**

El marco de muestreo utilizado fueron las unidades primarias de muestreo (UPM) que se formaron con la información estadística y cartográfica del II Conteo de Población y Vivienda 2005, además de la cartografía en formato individual de las UPM.

Muestreo

- **Formación de las unidades primarias de muestreo (UPM)**

Las unidades primarias de muestreo estuvieron constituidas por agrupaciones de viviendas con características diferenciadas dependiendo del ámbito al que pertenecen, como se especifica a continuación:

  - *En urbano alto*.- El tamaño mínimo de una UPM es de 80 viviendas habitadas y el máximo es de 160, pueden estar formadas por:
    - Una manzana.
    - La unión de dos o más manzanas contiguas del mismo AGEB.[1]
    - La unión de dos o más manzanas contiguas de diferentes AGEB de la misma localidad.
    - La unión de dos o más manzanas contiguas de diferentes localidades pero del mismo tamaño de localidad.

  - *En complemento urbano*.- El tamaño mínimo de una UPM es de 160 viviendas habitadas y el máximo es de 300, pueden estar formadas por:

[1] Área Geoestadística Básica.

- Una manzana
- La unión de dos o más manzanas contiguas del mismo AGEB.
- La unión de dos o más manzanas contiguas de diferentes AGEB y de la misma localidad.
- La unión de dos o más manzanas contiguas de diferentes AGEB de diferentes localidades del mismo municipio.

o *En rural*.- El tamaño mínimo de una UPM es de 160 viviendas habitadas y el máximo es de 300. Pueden estar formadas por*:*

- Un AGEB.
- Parte de un AGEB.
- La unión de dos o más AGEB colindantes del mismo municipio.
- La unión de una AGEB con parte de otra AGEB colindante del mismo municipio.

- **Estratificación**

La conformación de localidades diferenciadas por su tamaño, formaron de manera natural una primera estratificación geográfica. En la entidad, se distinguieron los tres ámbitos antes referidos, divididos a su vez en siete zonas, como se indica en la Tabla A:

Tabla A. Ámbito urbano y rural por zona y tamaño de la localidad.

Ámbito	Zona	Tamaño de localidad
Urbano	01	Ciudades autorrepresentadas con 100,000 o más habitantes
	02	Resto de las ciudades con 100,000 o más habitantes
	25	De 50,000 a 99,999 habitantes
	35	De 15,000 a 49,999 habitantes
	45	De 5,000 a 14,999 habitantes
	55	De 2,500 a 4,999 habitantes
Rural	60	Localidades con menos de 2,500 habitantes

De manera paralela, se utilizaron los cuatro estratos en los que se agruparon todas las UPM de Nuevo León, esta estratificación considera las características sociodemográficas de los habitantes de las viviendas, así como las características físicas y el equipamiento de las mismas, expresadas por medio de 24 indicadores (Tabla B) construidos con información del XII Censo General de Población y Vivienda 2000, para lo cual se emplearon métodos estadísticos

multivariados. Posteriormente, cada UPM clasificada con su estrato sociodemográfico fue asignada a su estrato geográfico.

Tabla B. Indicadores empleados en la estratificación de la muestra por ámbito de estudio.

Descripción del indicador	Ámbito de estudio			
	Nacional	Urbano Alto	Complemento Urbano	Rural
**Porcentaje de vivienda:**				
Que disponen de agua entubada dentro de la vivienda		X		
Con drenaje	X	X		X
Con electricidad				X
Que disponen de agua, luz y drenaje	X	X	X	X
Con piso diferente de tierra	X			X
Con paredes de material sólido				X
Con cocina exclusiva	X	X	X	X
Sin hacinamiento	X	X	X	X
Con servicio sanitario exclusivo con conexión de agua		X		
Con servicio sanitario exclusivo con admisión de agua	X		X	
Que utilizan gas para cocinar	X			X
Con radio o radiograbadora	X			X
Con televisión	X			
Con refrigerador	X	X	X	
Con licuadora	X			X
Con automóvil o camioneta propios	X	X	X	
Con videocasetera			X	
Con lavadora	X		X	
Con teléfono		X	X	
Con calentador de agua		X	X	
Con cuatro bienes (teléfono, refrigerador, lavadora y boiler)		X		
Con cuatro bienes (radio, televisión, licuadora y refrigerador)	X		X	
Con el mínimo equipamiento (radio o televisión y licuadora)				X
**Porcentaje de la población:**				
Derechohabiente a servicio de salud		X	X	
De 6 a 17 años que asiste a la escuela	X	X	X	
De 6 a 14 años que asiste a la escuela				X
De 15 años y más alfabeta	X			X
De 15 años y más con posprimaria	X	X	X	X
Grado promedio de escolaridad	X	X	X	X
Ocupada que gana más de 2.5 salarios mínimos	X	X	X	X
Ocupada que gana más de 5 salarios mínimos	X	X	X	
Femenina de 12 años y más económicamente activa	X	X	X	
Económicamente activa de 20 a 49 años	X	X	X	
**Otros:**				
Porcentaje de hogares en el decil nacional 8, 9 y 10	X	X	X	
Relación de dependencia económica	X	X	X	
Total de indicadores	24	21	21	16

- **Esquema de muestreo**

El esquema de muestreo, se caracterizó por ser probabilístico, estratificado, polietápico, y por conglomerados.

- *Probabilístico*

  Las unidades de selección tuvieron una probabilidad conocida y distinta de cero de ser seleccionadas.

- *Estratificado*

  Las unidades primarias de muestreo con características similares se agruparon para formar estratos.

- *Polietápico*

  Las unidades últimas de muestreo (personas) fueron seleccionadas en cuatro etapas.

- *Por conglomerados*

  Las unidades de muestreo que se seleccionaron en cada etapa fueron conjunto de manzanas, viviendas o personas, que al final permitieron seleccionar al entrevistado.

Tamaño de la muestra

Para obtener el tamaño de muestra se empleó la siguiente ecuación:

$$n = \frac{z^2 \, q \, DEFF}{r^2 p \left(1 - tnr\right) PHV}$$

Donde:

n	=	tamaño de la muestra en viviendas
p	=	proporción de interés a estimar
q	=	1-p
DEFF	=	efecto de diseño definido como el cociente de la varianza en la estimación del diseño utilizado, entre la varianza obtenida considerando un muestreo aleatorio simple para un mismo tamaño de muestra
z	=	es el valor asentado en tablas estadísticas de la distribución normal, para una confianza prefijada
tnr	=	tasa de no respuesta máxima esperada
r	=	error relativo máximo aceptable
PHV	=	promedio de entrevistas a realizar por vivienda

Con una confianza del 90%, un efecto de diseño de 1.5, una proporción del 8%, un error relativo esperado del 15%, una tasa de no respuesta máxima esperada del 40%, un promedio de 2.09 entrevistas por vivienda, se obtuvo un tamaño de muestra de 1,654.6 por región. Aplicando a las 4 regiones de Nuevo León, se obtuvo un tamaño de muestra de 6,618.4 viviendas el cual fue ajustado a 6,642 viviendas para la entidad. Dicho tamaño alcanza para estimar proporciones mínimas del 9.0% de 0 a 9 años, 10.0% de 10 a 19 años, 5.0% de 20 a 59 años y 17.0% de población de 60 años y más, a nivel entidad.

- **Distribución de la muestra**

  La muestra se distribuyó de manera proporcional al total poblacional en el ámbito urbano y rural, así como en los 4 estratos sociodemográficos correspondientes a Nuevo León, para cada región.

$$n_h = \frac{N_h}{N} n$$

Donde:

n_h = número de viviendas en muestra en el h-ésimo estrato

n = número total de viviendas en muestra

N_h = población total, en el h-ésimo estrato

N = población total

En la Tabla C se presenta la distribución de la muestra en viviendas por región y estrato, en la Tabla D se presenta la distribución de la muestra de UPM por región y estrato.

Tabla C. Distribución de la muestra de viviendas.

Muestra viviendas	Estrato	Urbano				Rural				Total
	Dominio	1	2	3	4	1	2	3	4	
Región	1. Metropolitana	40	500	320	200	40	80	0	0	1 180
	2. Central	0	420	340	100	120	200	0	0	1 180
	3. Norte	0	460	320	0	80	320	0	0	1 180
	4. Sur	0	100	80	0	900	100	0	0	1 180
Total										4 720

Tabla D. Distribución de la muestra de unidades primarias de muestreo (UPM).

Muestra UPM	Estrato	Urbano				Rural				Total
	Dominio	1	2	3	4	1	2	3	4	
Región	1. Metropolitana	2	25	16	10	2	4	0	0	59
	2. Central	0	21	17	5	6	10	0	0	59
	3. Norte	0	23	16	0	4	16	0	0	59
	4. Sur	0	5	4	0	45	5	0	0	59
Total										236

Selección de la muestra

La selección de la muestra se realizó en forma independiente en cada ámbito urbano y rural, y al interior de estos en los estratos estatales. El procedimiento fue el siguiente:

- *En el ámbito urbano*

  a) En la primera etapa se seleccionaron n_h UPM con probabilidad proporcional al número de viviendas, en el h-ésimo estrato
  b) En cada UPM, se seleccionaron dos manzanas con igual probabilidad
  c) En cada manzana, se seleccionaron 10 viviendas con igual probabilidad
  d) En cada vivienda se seleccionó una persona con igual probabilidad por grupo de edad

Por lo tanto la probabilidad de seleccionar una persona en la l-ésima vivienda, en la j-ésima manzana, en la i-ésima UPM, en el h-ésimo estrato es:

$$P\left(X'_{hij\ell}\right) = \frac{n_h V_{hi}}{V_h} \cdot \frac{2}{m_{hi}} \cdot \frac{10}{V_{hij}} \cdot \frac{1}{G_{hij\ell}}$$

Su factor de expansión[2] está dado por:

$$F_{hij\ell} = \frac{V_h m_{hi} V_{hij} G_{hij\ell}}{20 n_h V_{hi}}$$

[2] El factor de expansión se define como el inverso de la probabilidad de selección.

Donde:

n_h = número de UPM a seleccionar en el h-ésimo estrato

V_h = número de viviendas en el h-ésimo estrato, según II Conteo de Población y Vivienda 2005

V_{hi} = número de viviendas en la i-ésima UPM, en el h-ésimo estrato, según II Conteo de Población y Vivienda 2005

V_{hij} = número de viviendas en la j-ésima manzana, en la i-ésima UPM, en el h-ésimo estrato, según II Conteo de Población y Vivienda 2005

m_{hi} = número de manzanas en la i-ésima UPM, en el h-ésimo estrato

$G_{hij\ell}$ = total de personas de un grupo de edad, en la l-ésima vivienda, en la j-ésima manzana, en la i-ésima UPM, en el h-ésimo estrato

- *En el ámbito rural*

a) En la primera etapa se seleccionaron n_h UPM con probabilidad proporcional al número de viviendas, en el h-ésimo estrato

b) En cada UPM se seleccionaron 2 manzanas o localidades con igual probabilidad

c) En cada manzana o localidad, se seleccionaron 10 viviendas con igual probabilidad

d) En cada vivienda, se seleccionó una persona con igual probabilidad por grupo de edad

Por lo tanto la probabilidad de seleccionar una persona en la l-ésima vivienda, en la j-ésima localidad, en la i-ésima UPM, en el h-ésimo estrato es:

$$P\left(X'_{hij\ell}\right) = \frac{n_h V_{hi}}{V_h} \cdot \frac{2}{m_{hi}} \cdot \frac{10}{V_{hij}} \cdot \frac{1}{G_{hij\ell}}$$

Su factor de expansión está dado por:

$$F_{hij\ell} = \frac{V_h m_{hi} V_{hij} G_{hij\ell}}{20 n_h V_{hi}}$$

Donde:

n_h = número de UPM a seleccionar en el h-ésimo estrato

V_h = número de viviendas en el h-ésimo estrato, según II Conteo de Población y Vivienda 2005

V_{hi} = número de viviendas en la i-ésima UPM, en el h-ésimo estrato, según II Conteo de Población y Vivienda 2005

V_{hij} = número de viviendas en la j-ésima manzana, en la i-ésima UPM, en el h-ésimo estrato, según II Conteo de Población y Vivienda 2005

m_{hi} = número de manzanas en la i-ésima UPM, en el h-ésimo estrato

$G_{hij\ell}$ = total de personas de un grupo de edad, en la l-ésima vivienda, en la j-ésima manzana, en la i-ésima UPM, en el h-ésimo estrato.

Obtención de la información

- **Instrumentos de recolección**

La EESN-NL 2011/2012 está constituida por cinco cuestionarios que contienen 35 secciones y 969 ítems con los cuales se obtuvo información de los principales grupos poblacionales (niños, adolescentes, adultos y adultos mayores) considerando aspectos de salud y nutrición, además se contempló la información sobre hogares, utilización de servicios de salud y programas de ayuda alimentaria.

- *Cuestionario HOGAR*[3,4]

Abordó elementos para su identificación, características sociodemográficas, características de la vivienda, activos del hogar, situación de salud y servicios de salud, gastos en el hogar, difusión, aceptación y utilización del Seguro Popular, utilización de servicios de salud. Además, incluyó información relativa a los programas de ayuda alimentaria, así como medidas antropométricas para la determinación del estado de nutrición por grupos de edad y presión arterial.

---

[3] Hogar es el conjunto de personas, relacionadas o no por algún grado de parentesco, que habitualmente duermen en una misma vivienda o bajo el mismo techo, beneficiándose de un ingreso común, aportado por uno o más de los miembros del hogar.
[4] Por razones operativas no se incluyeron dentro de la encuesta las viviendas colectivas como instalaciones militares, cárceles, conventos, hoteles, asilos y similares.

El número final de hogares en la encuesta fue de 4,236 hogares con un total de 13,409 individuos, de los cuales se extrajo la muestra para cada grupo de edad, independientemente del número de individuos en el hogar, sólo se eligió uno por grupo de edad para los cuestionarios individuales.

- *Cuestionarios individuales*.- A continuación se describen los elementos considerados en cada uno de los cuestionarios.

-Niños de 0 a 9 años

Indicadores positivos y factores de riesgo, vacunación, enfermedades diarreicas, infecciones respiratorias agudas, actividad física, accidentes, prácticas de lactancia, alimentación complementaria, frecuencia de consumo de alimentos y consumo de alimentos fuera de casa.[5] La muestra final para este grupo de edad fue de 1,372 niños.

- *Adolescentes de 10 a 19 años*

Factores de riesgo, infecciones de trasmisión sexual y salud reproductiva, actividad física, accidentes, agresión y violencia, frecuencia de consumo de alimentos y consumo de alimentos fuera de casa. La muestra final para este grupo de edad fue de 1,319 adolescentes.

-Adultos de 20 a 59 años

Actividad física, depresión, enfermedades crónico-degenerativas, salud reproductiva, accidentes, factores de riesgo, frecuencia de consumo de alimentos y consumo de alimentos fuera de casa. La muestra final para este grupo de edad fue de 3,125 adultos.

- *Adultos mayores de 60 años y más*

Además de lo referido en los cuestionarios de adultos, este cuestionario contiene la evaluación mínima mental, dependencia física, riesgo de nutrición, frecuencia alimentaria, apetito, síndrome de fragilidad, riesgo de depresión y antropometría. La muestra final fue de 1,474 adultos de 60 años y más.

[5] Formato utilizado para niños de 6 a 15 años, en este caso aplica para niños de 6 a 9 años

- **Capacitación del equipo de campo**

El grupo de encuestadores fue conformado por profesionales de la salud, principalmente nutriólogos, los cuales fueron capacitados y estandarizados en cuanto a la toma de las medidas antropométricas, aplicación de la sección de frecuencia alimentaria y toma de presión arterial.

La información en cada hogar se recabó en dos días: en el primero se verificó la composición del hogar de acuerdo con la información proporcionada por los cartógrafos; se solicitó la firma de la carta de consentimiento informado; se obtuvieron las mediciones antropométricas; la información referente a la frecuencia del consumo de alimentos en el hogar y fuera de él; y aquella sobre los programas de ayuda alimentaria.

Los días hábiles para los equipos fueron de lunes a viernes; en algunos casos, se utilizaron los sábados para recuperar la información no obtenida durante la semana.

- **Levantamiento**

El levantamiento de la información de la EESN-NL 2011/2012 se llevó a cabo en las cuatro regiones de Nuevo León, realizándose en primera instancia en el área metropolitana; posteriormente y de manera simultánea, en las tres regiones restantes. La aplicación de los instrumentos de recolección de información se realizó a través de entrevista directa.

Para la Región Metropolitana el personal de campo estuvo integrado por 50 profesionales de salud, entre ellos 18 nutriólogos, agrupados en 10 equipos de trabajo. Para las regiones restantes se contó con la participación de 76 profesionales de la salud, entre estos 23 nutriólogos, distribuidos en equipos conformados de acuerdo a la complejidad y accesibilidad de las localidades (Anexo 1).

- **Etapa primaria**
    - *Ubicación de la vivienda*: Seleccionada la vivienda en el muestreo, se realizó la ubicación física de la misma a fin de verificar que ésta se encontrara habitada; si la vivienda no se encontraba habitada o la familia no aceptaba participar, ésta era descartada.

- *Selección del hogar*: En los casos en que existía más de un hogar en una vivienda, se realizó una selección aleatoria a fin de determinar cuál de ellos sería objeto de participación, procediéndose a dar lectura y firmar la carta de consentimiento informado; posteriormente se aplicó el cuestionario HOGAR. Si la vivienda había sido seleccionada para la toma de muestra de sangre, se programó una nueva cita.

- *Selección de los individuos por grupo de edad*: Después de la aplicación del cuestionario HOGAR, si existía más de un miembro de familia por grupo de edad, se seleccionó a uno de ellos, que fue quien presentó la fecha de cumpleaños más cercana a la fecha de la aplicación de los cuestionarios, de manera que, estuvieron enterados a quienes se les aplicaría la batería de cuestionarios, a fin de asegurar la presencia de los miembros seleccionados del hogar en cuestión. En los casos en que alguno de ellos no estuvo presente, se estableció una nueva cita para la aplicación del cuestionario.

- **Etapa secundaria**

Para la toma de muestras sanguíneas, se eligió una submuestra de 640 viviendas por región calculada con el 95.0% de confianza y una precisión del 10.0%; el muestreo se realizó de manera aleatoria simple sin reposición. Para los exámenes de genética los hogares fueron seleccionados de manera aleatoria simple y sin reposición de las 640 viviendas a los sujetos de 1000 viviendas seleccionadas aleatoriamente de la muestra calculada para la EESN-NL 2011/2012.

Para las determinaciones bioquímicas y de genética se utilizaron muestras de sangre venosa que fueron extraídas de los sujetos encuestados en el hogar.

Partiendo de una muestra de sangre completa recolectada en un tubo lila con EDTA, se tomaron 200uL de la capa de leucocitos para extraer y purificar los ácidos nuclicos (ADN), para su posterior cuantificación y análisis.

Las muestras se procesaron en el equipo QIAcube de marca de Qiagen, haciendo uso del kit QIAamp® DNA Blood Mini, el equipo realizó la lisis de la muestra y la purificación del ADN mediante columnas de purificación.

Procesamiento de muestras de sangre

Las muestras fueron procesadas en el Laboratorio Clínico del Hospital Metropolitano de Monterrey "Dr. Bernardo Sepúlveda" de la Secretaría de Salud de Nuevo León, bajo las condiciones y criterios metodológicos establecidos utilizando el equipo COBAS 6000 de Roche. (Tabla E).

Tabla E. Procesamiento de muestras de laboratorio.

Prueba	Metodología	Muestras	Estabilidad de la muestra	
**Glucosa**	Enzimático de referencia hexoquinasa.	Suero, plasma con heparina de litio, plasma con fluoruro.	A temperatura ambiente sin centrifugar, la glucosa sérica disminuye 7% cada hora (glucólisis).	
			Suero sin hemólisis:	8 horas a 15-25ºC<br>72 horas a 2-8ºC
			Plasma con fluoruro:	24 horas a 15-25ºC
**Urea**	Enzimático de referencia ureasa y glutamato deshidrogenasa.	Suero, plasma con heparina de litio o EDTA.	Suero o plasma:	7 días a 15-25ºC<br>7 días a 2- 8ºC<br>1 año a -15 a -25ºC
**Creatinina**	Enzimático reacción de JAFFÉ.	Suero, plasma con heparina de litio.	Suero o plasma.	7 días a 15-25ºC<br>7días a 2-8ºC<br>3 meses a -15-25ºC
**Ac. Úrico**	Enzimático colorimétrico uricasa.	Suero, plasma con heparina de litio.	Suero o plasma.	5 días a 2 - 8ºC<br>6 meses a -15-25ºC
**AST**	Enzimático NADH-NAD	Suero, plasma con heparina de litio.	Suero o plasma:	24 hrs a -15 -25 ºC<br>7 días 2-8ºC
**ALT**	Enzimático NADH-NAD	Suero, plasma con heparina de litio.	Suero o plasma:	3 días a -15 -25ºC<br>7 días 2- 8 ºC<br>>7 días -70ºC

Tabla E. Continuación.

Prueba	Metodología	Muestras	Estabilidad de la muestra	
**GGT**	Enzimático colorimétrico	Suero, plasma con heparina de litio.	Suero o plasma:	7 días a 15 - 25ºC<br>7días 2 - 8ºC<br>1año a -15 - 25ºC
**Triglicéridos**	Enzimático colorimétrico	Suero, plasma con heparina de litio.	Suero o plasma:	5 a 7 días 2 - 8ºC<br>3 meses a-15-25ºC
**Colesterol**	Enzimático colorimétrico	Suero, plasma con heparina de litio.	Suero o plasma:	7 días 15 a 25ºC<br>7 días a 2 a 8 ºC<br>3 meses a-15-25ºC

Captura de la información

La lectura electrónica de los cuestionarios fue realizada por el Centro de Evaluaciones de la Universidad Autónoma de Nuevo León, generando las bases de datos correspondientes en formato Excel.

Auditoría de la base de datos

El proceso para la auditoría de la base de datos fue realizado en el Laboratorio de Computación de la Facultad de Salud Pública y Nutrición de la UANL, con la participación de 62 estudiantes de 9° y 10° semestre de la Licenciatura en Nutrición. La auditoría se realizó por tipo de cuestionario cotejando la información contenida en las bases de datos con la de los cuestionarios; se seleccionó una muestra representativa aleatoria.

Para calcular el tamaño de la muestra se utilizó la siguiente fórmula con un nivel de confianza del 95%, un error entre el 5 y 10% y la varianza de 0.25:

$$n = \frac{N}{1 + \frac{\varepsilon^2 (N-1)}{z^2 pq}}$$

Donde:

n=es el tamaño de la muestra que deseamos conocer
N=tamaño conocido de la población
ε=margen de error
pq=varianza de la población
z=valor de z correspondiente al nivel de confianza

Tabla F. Distribución de resultados de auditoría de la encuesta.

Resultado	Cuestionarios				
	0-9 años	10-19 años	20-59 años	60 y más años	Hogar
Total de encuestas	1,476	1,332	2,798	1,425	4,251
Encuestas revisadas	90	100	311	108	559
% Encuestas revisadas	6.1%	7.5%	11.1%	7.5%	13.2%
Confianza	95%	95%	95%	95%	95%
Campos analizados	37,350	34,440	70,286	38,772	68,198
Campos con error	73	39	421	79	440
% de error	0.2%	0.1%	0.6%	0.2%	0.7%

Procesamiento de la base de datos

Para la obtención de las diferentes estimaciones estadísticas (medias, proporciones o porcentajes) se utilizó el programa estadístico computacional SPSS versión 17, y en el caso de las estimaciones puntuales se aplicó el factor de expansión de hogar o individual según correspondiera al nivel de la variable de interés con los ponderadores o pesos. Para el cálculo de intervalos de confianza de las prevalencias se utilizó un método de cálculo asintótico conocido como "Score Method".

Consideraciones éticas

Los individuos seleccionados para la aplicación de los cuestionarios y obtención de muestra sanguínea firmaron un consentimiento informado; en el caso de los menores de edad fueron los padres o tutores quienes autorizaron formar parte de la encuesta estatal. El proyecto en su conjunto, así como los procesos y formatos de la encuesta, fueron aprobados por el Comité de Investigación de la Facultad de Salud Pública y Nutrición de la Universidad Autónoma de Nuevo León.

Capítulo 1

Perfil Sociodemográfico

1. Perfil Sociodemográfico

Tradicionalmente, los estudios sociodemográficos sobre la familia se han centrado en el análisis del hogar para abordar las formas en que los grupos domésticos se organizan para llevar a cabo la subsistencia cotidiana y para reproducirse a través del tiempo. El hogar es un grupo de individuos, emparentados o no, que comparten alimentos y gastos dentro de un mismo ámbito doméstico; representa una pequeña colectividad social en donde sus integrantes comparten una identidad común —sustentada generalmente en el parentesco— y cierto sentido de solidaridad derivado de una residencia y una economía común. Esto supone que los miembros de un hogar no sólo están unidos por lazos de sangre, adopción o alianza, sino que establecen relaciones interdependientes para satisfacer sus necesidades, asignar, cumplir deberes y responsabilidades en función de características demográficas y sociales individuales [López, 2001]. Para el Instituto Nacional de Estadística y Geografía (INEGI) los hogares están conformados por personas que pueden ser o no familiares, que comparten la misma vivienda y se sostienen de un gasto común. La Encuesta Nacional de Salud y Nutrición (ENSANUT 2006) estimó que existen 23, 759,124 hogares en todo el país, de los cuales 4.1% corresponden a Nuevo León. De acuerdo a los datos de INEGI para el 2010, en Nuevo León había 1, 191,114 hogares.

En los últimos sesenta años, la sociedad mexicana ha experimentado profundas transformaciones de carácter económico, político, social y cultural. El cambio demográfico y las transformaciones en los patrones de nupcialidad, las nuevas pautas reproductivas y la creciente presencia de las mujeres en ámbitos extra domésticos, particularmente en la escuela y el trabajo, son factores que han contribuido a conformar nuevos escenarios sociales. La familia no ha sido ajena a estos procesos de cambio. Se considera que todas estas transformaciones han influido en formas variadas y diversas sobre el tamaño, la estructura y la composición de la familia, las familias están cambiando con mayor rapidez que en el pasado [López, 2001]. Con el objetivo de establecer un perfil sociodemográfico de los habitantes del estado de Nuevo León en torno a las características del hogar, viviendas, población, escolaridad, condición laboral, participación económica y su ingreso se recabó información de 4,236 hogares que representan 751,823 hogares de Nuevo León, de los cuales el 24.3% corresponde a la Región Metropolitana, 24.6% a la Región Norte, 25.4% a la Región Sur y un 25.9% a la Región Centro.

Características de la población

La Figura 1.1 presenta la pirámide de población de la EESN-NL 2011/2012, de acuerdo a género y grupo de edad; la proporción es similar en la distribución por género, 50.4% corresponde a hombres y 49.6% mujeres, mientras que en la distribución por grupo de edad los de 0 a 15 años son los que concentran el mayor porcentaje, seguido por el grupo de 30 a 44 años. Se observa una similitud con la pirámide de población 2010 reportada por INEGI (Figura 1.2), así como la distribución regional donde solo la Región Sur muestra un mayor porcentaje de adolescentes y población que alcanza los 100 años de edad (Figura 1.3).

Figura 1.1 Pirámide de Población Nuevo León según la EESN-NL 2011/2012.

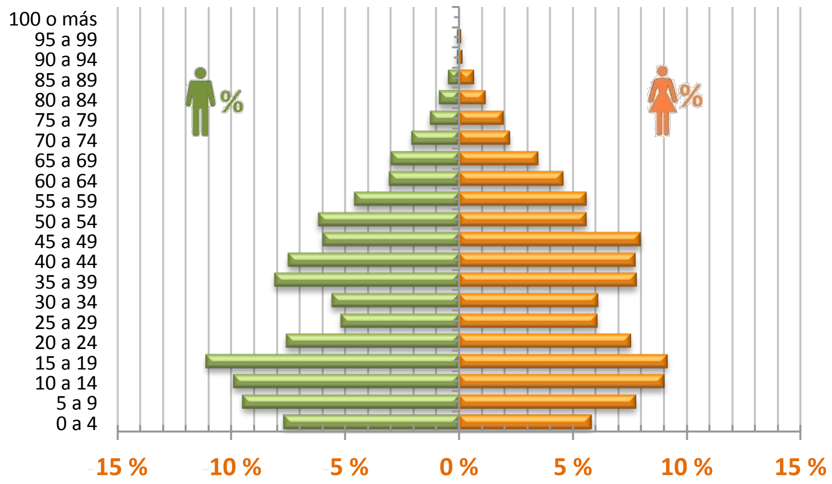

Figura 1.2 Pirámide de Población Nuevo León según INEGI 2010.

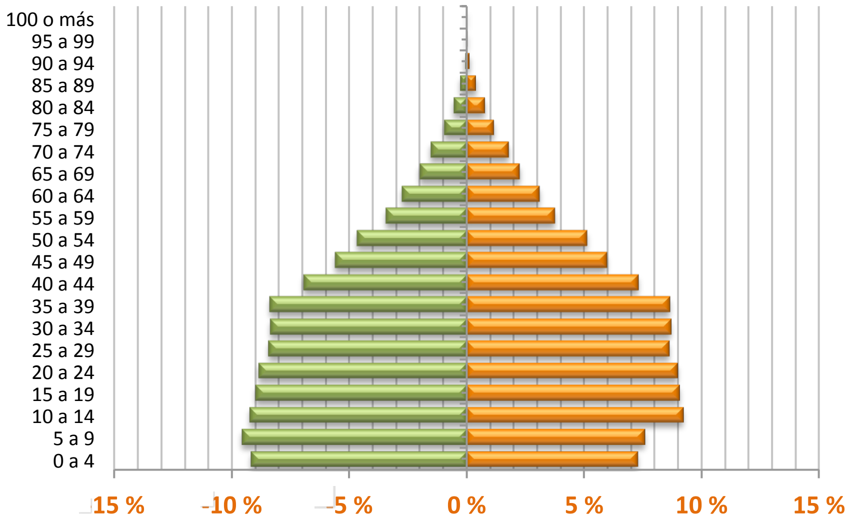

Figura 1.3 Pirámide poblacional por regiones.

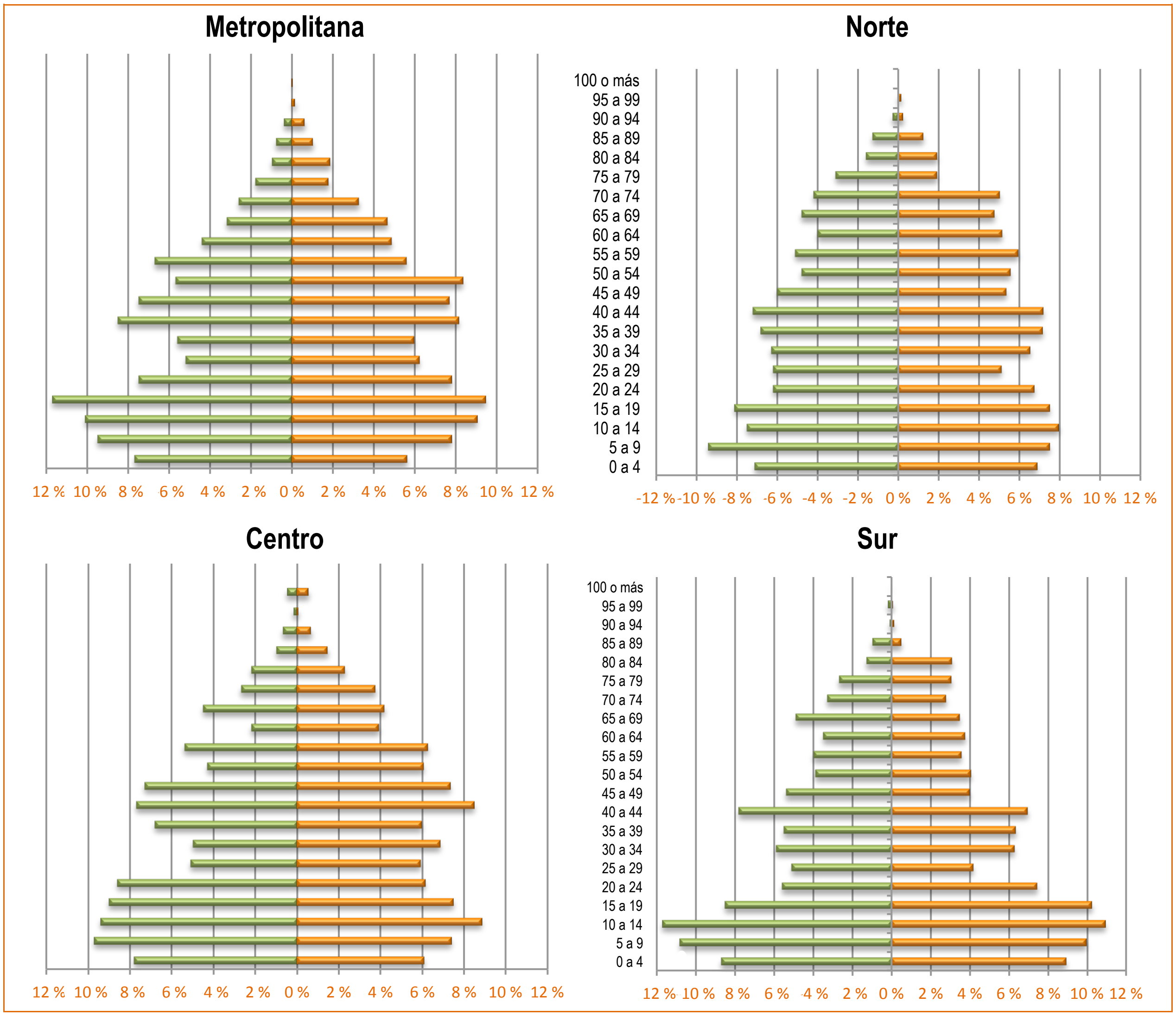

Fuente: EESN-NL (Cuestionarios de grupos de edad).

Con respecto al número de personas por vivienda, la Figura 1.4 muestra que el mayor porcentaje fue de 3 a 4 personas por vivienda en un 21.3% y 21.2% respectivamente, por lo que el promedio es de 3.7 individuos por vivienda, dato similar al reportado por la ENSANUT-2012 (3.9 individuos por hogar). En la Región Metropolitana se observa un promedio de 3 habitantes por vivienda, mientras que en la Región Norte, Sur y Centro el promedio es de dos habitantes por vivienda (Figura 1.5).

Figura 1.4 Número de habitantes por hogar.

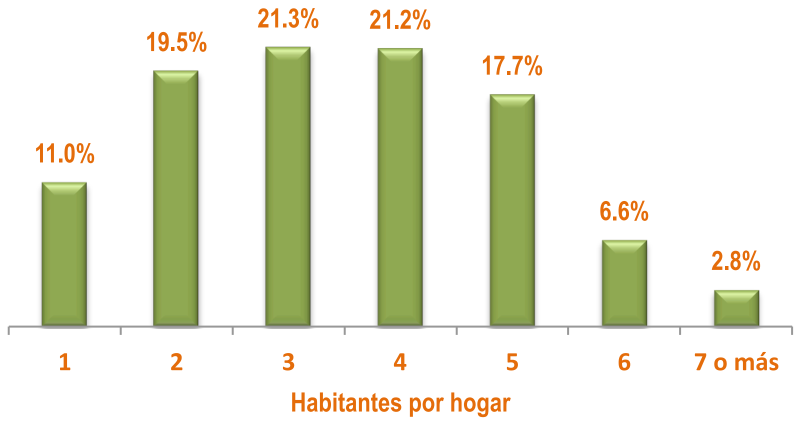

Fuente: EESN-NL 2011/2012 (Cuestionario Hogar).

Figura 1.5 Número de habitantes por hogar, dividido por región geográfica.

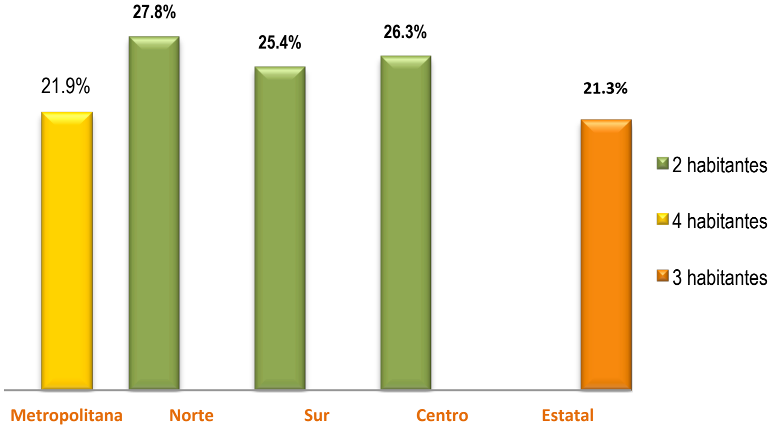

Fuente: EESN-NL 2011/2012 (Cuestionario Hogar).

La composición de las familias en Nuevo León presenta las siguientes características: el 27.5% son jefes de familia, 39.5% son hijos y 21.2% son esposas o compañeros. Los hogares de acuerdo al género del jefe de la familia se distribuyen de la siguiente forma (Figura 1.6)

Figura 1.6 Jefatura del hogar según género.

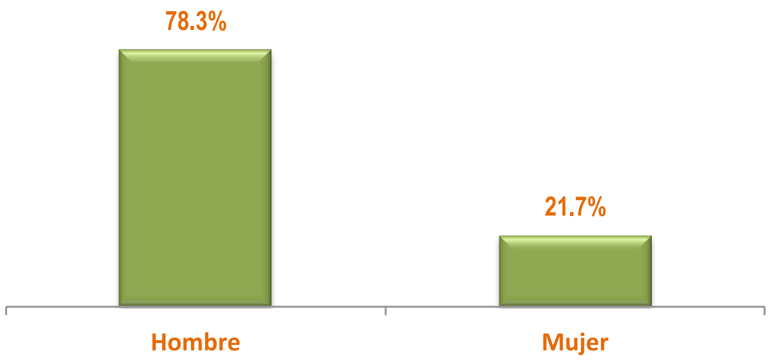

Fuente: EESN-NL 2011/2012 (Cuestionario Hogar).

Se observa que en Nuevo León el 21.7% de los hogares tienen como jefe de familia a mujeres, valor muy similar a los reportados en la Encuesta Nacional de Dinámica Demográfica 2009 (22.7%) y la ENSANUT 2012 (25.7%) [INEGI, 2009].

Figura 1.7 Jefatura del hogar según género, dividido por región geográfica.

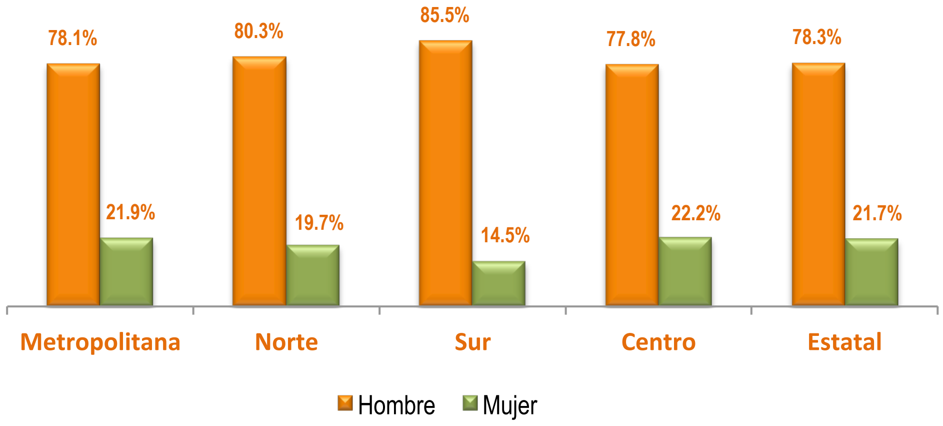

Fuente: EESN-NL 2011/2012 (Cuestionario Hogar).

De acuerdo a estado civil el 50.7% de la población están casados, 33.5% son solteros, 6.1% viven en unión libre y 9.7% están separados, divorciados o viudos (Figura 1.8); a nivel regional más del 50.0% de la población refiere estar casado (Tabla 1.1.).

Figura 1.8 Población según estado civil.

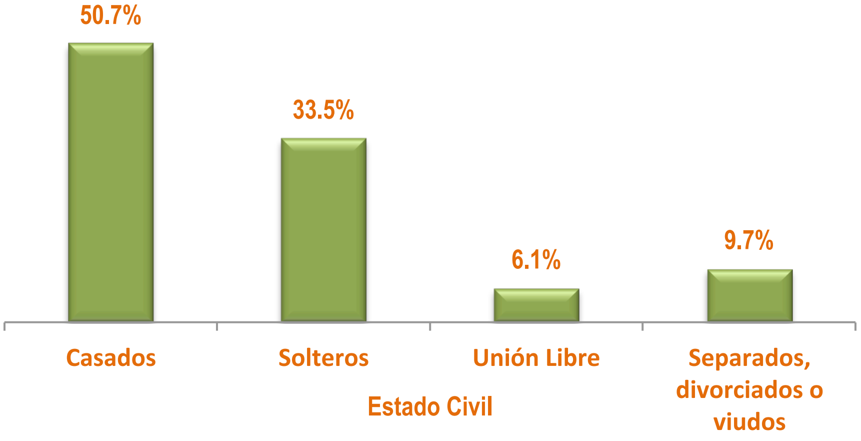

Fuente: Fuente: EESN-NL 2011/2012 (Cuestionario Hogar).

Tabla 1.1 Población según su estado civil, dividido por región geográfica.

Estado Civil	Región			
	Metropolitana %	Norte %	Sur %	Centro %
Unión libre	5.7	9.5	7.4	7.4
Casado(a)	49.9	52.1	56.6	54.3
Separado	2.8	2.2	1.6	2.2
Divorciado(a)	1.9	0.9	0.0	1.8
Viudo(a)	5.0	7.9	5.3	5.3
Soltero(a)	34.6	27.4	29.1	29.1
Total	100.0	100.0	100.0	100.0

Fuente: EESN-NL 2011/2012 (Cuestionario Hogar).

En relación a la presencia de mascotas, 52.9% de los hogares poseen al menos una mascota; la mascota más frecuente son los perros con un 41.9%, seguido de las aves 4.9%, gatos 3.3% y otros tipos de mascotas el 2.8% (Figura 1.9).

Figura 1.9 Presencia de mascotas en los hogares.

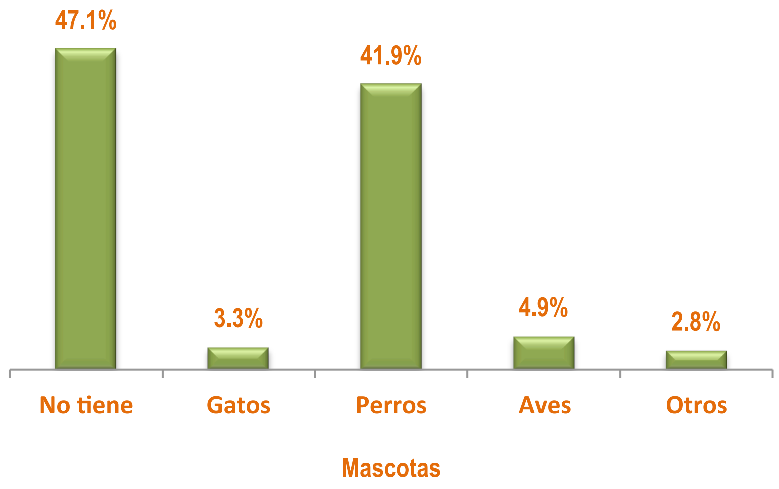

Fuente: EESN-NL 2011/2012 (Cuestionario Hogar).

A nivel regional, el mayor porcentaje de viviendas con presencia de mascotas es de 45.0% a 63.6%, específicamente de presencia de perros los porcentajes son del 45.0% a 63.6% principalmente en las regiones Norte, Sur y Centro, lo que supone una relación con la dispersión geográfica de las viviendas y el tipo de actividad realizada (Tabla 1.2).

Tabla 1.2 Población según mascotas presentes en la vivienda, dividida por región geográfica.

Mascotas	Región			
	Metropolitana %	Norte %	Sur %	Centro %
Aves	4.8	2.5	2.6	7.1
Perros	40.7	45.0	63.6	45.0
Gatos	2.8	7.5	2.6	5.2
Otros	2.9	4.5	1.3	1.7
No tiene	48.8	40.5	29.9	41.0
Total	100.0	100.0	100.0	100.0

Fuente: EESN-NL 2011/2012 (Cuestionario Hogar).

• Escolaridad

La población mayor de diez años que refirió saber leer y escribir fue el 97.0%, mientras que un 3.0% fueron identificados como analfabetas, los cuales representan a 96,865 personas, proporción ligeramente inferior al reportado por la ENSANUT-NL 2006 de 3.7% (Figura 1.10).

Figura 1.10 Población mayor de diez años según condición de alfabetismo.

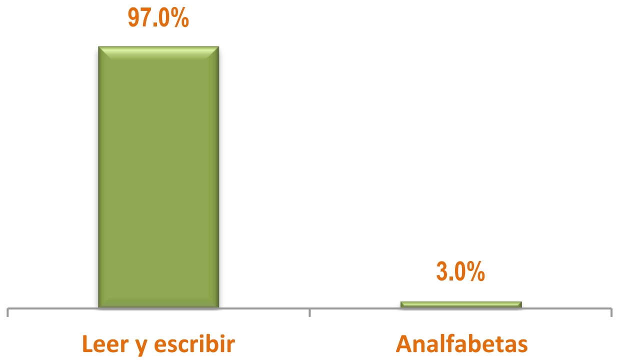

Fuente: EESN-NL 2011/2012 (Cuestionario Hogar).

El nivel de alfabetismo en Nuevo León, considerando todos los grupos de edad es de 94.2% mientras que 5.8% no saben leer y escribir; el mayor índice de analfabetismo se observa en las Regiones Sur y Centro con 10.2% y 7.1% respectivamente (Tabla 1.3).

Tabla 1.3 Nivel de alfabetismo de la población en general, dividido por región geográfica.

Pertenencia	Región			
	Metropolitana %	Norte %	Sur %	Centro %
Sí	94.7	93.1	89.8	92.9
No	5.3	6.9	10.2	7.1
Total	100.0	100.0	100.0	100.0

Fuente: EESN-NL 2011/2012 (Cuestionario Hogar).

La población en edad escolar según asistencia a la escuela se representa en la Figura 1.11 donde se observa que en el grupo de edad de 6 a 14 años el 98.5% sí asiste a la escuela, mientras que el 1.5% de la población que conforma este grupo no acude a la escuela; este último porcentaje

representa a 9,391 niños del grupo aquí citado. Mientras que para el grupo de 15 a 24 años el 55.5% sí acude y el 44.5% no acude. La ENSANUT 2012 al respecto reporta que el 95.0% del grupo de 6-14 años sí asiste y del grupo de 15-24 es 41.1%, cifras que están por debajo de lo encontrado en la EESN-NL 2011/2012.

Figura 1.11 Población de 6 a 24 años según asistencia a la escuela.

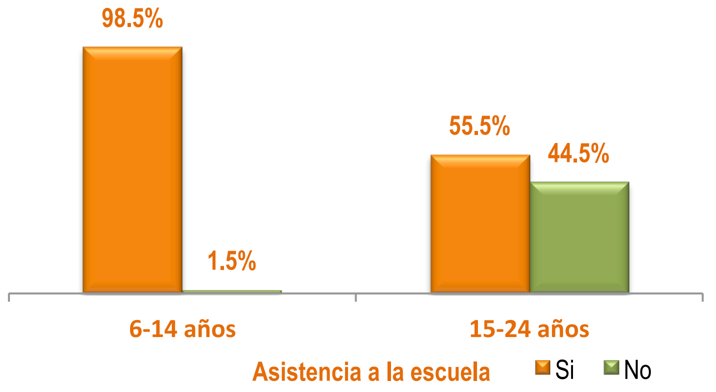

Fuente: EESN-NL 2011/2012 (Cuestionario Hogar).

Al preguntar a la población si alguna vez ha ido a la escuela, para todos los grupos de edad, 95.4% respondió que sí; el mayor porcentaje de asistencia se observa en los grupos de edad de 10 a 19 años y de 20 a 59 años, en un 99.2% y 97.2% respectivamente (Tabla 1.4).

Tabla 1.4 Población que alguna vez ha ido a la escuela por grupo de edad.

Pertenencia	Grupo de edad				Total
	0-9 años %	10-19 años %	20-59 años %	60 años y más %	%
Sí	86.9	99.2	97.2	89.7	95.4
No	13.1	0.8	2.8	10.3	4.6
Total	100.0	100.0	100.0	100.0	100.0

Fuente: EESN-NL 2011/2012 (Cuestionario Hogar).

Según el último grado escolar de la población, la distribución se concentra en los siguientes tres niveles de escolaridad: primaria 31.6%, secundaria 27.1% y licenciatura 11.7% (Tabla 1.5).

Tabla 1.5 Población según último grado de escolaridad aprobado por grupos de edad.

Grado de escolaridad	Grupo de edad 3 a 5 años %	6 a 12 años %	13 a 15 años %	16 a19 años %	20 a 24 años %	25 años y más %	Total %
Ninguno	22.5	1.1	0.2	0.3	0.4	2.8	2.5
Preescolar o kinder	77.5	17.6	0.6	2.5	0.0	0.6	4.9
Primaria	0.0	81.4	19.7	5.5	4.3	30.2	31.6
Secundaria	0.0	0.0	79.6	37.4	29.6	26.3	27.1
Carrera técnica o comercial (después de secundaria)	0.0	0.0	0.0	5.5	3.1	5.5	4.1
Preparatoria o vocacional	0.0	0.0	0.0	43.2	18.5	7.8	9.8
Bachillerato técnico	0.0	0.0	0.0	5.3	5.7	1.4	1.7
Carrera técnica o comercial (después de preparatoria)	0.0	0.0	0.0	0.0	6.7	5.9	4.3
Normal superior	0.0	0.0	0.0	0.0	0.6	0.8	0.5
Licenciatura	0.0	0.0	0.0	0.0	28.7	14.3	11.7
Maestría	0.0	0.0	0.0	0.0	0.0	2.3	1.4
Doctorado	0.0	0.0	0.0	0.0	0.0	0.1	0.1
No sabe	0.0	0.0	0.0	0.3	0.3	0.3	0.2
Total	100.0	100.0	100.0	100.0	100.0	100.0	100.0

Fuente: EESN-NL 2011/2012 (Cuestionario Hogar).

• Condición de la actividad productiva en la semana anterior a la encuesta

Según la actividad laboral la semana anterior a la encuesta, se observa que de la población mayor a 10 años el 41.3% se encontraba trabajando, 17.3% eran estudiantes, 1.8% buscaba trabajo, 4.9% eran jubilados y 19.5% no trabajaba (Tabla 1.6). Este mismo dato reportado en el Censo 2010 refiere que 45.6% trabajaba y 28.2% no trabajaba [INEGI, 2010].

Tabla 1.6 Población según su actividad laboral en la semana previa a la encuesta por grupo de edad.

Actividad laboral	10 a 19 años %	20 a 59 años %	60 años y más %	Total %
Trabajó	7.7	58.3	19.8	41.3
Tenía trabajo pero no trabajó	0.0	1.4	0.3	0.9
Buscó trabajo	0.3	2.6	0.6	1.8
Estudiante	66.0	4.4	0.7	17.3
Quehaceres del hogar	1.9	21.2	26.9	17.8
Es jubilado o pensionado	0.0	1.3	26.2	4.9
Incapacitado permanentemente para trabajar	0.0	0.1	0.0	0.1
No trabaja	28.4	14.1	28.7	19.5
Trabajó ayudando en el negocio, predio o rancho de la familia sin recibir pago	1.0	0.7	1.0	0.8

Fuente: EESN-NL 2011/2012 (Cuestionario Hogar).

Características del hogar y las viviendas

Considerando el número de cuartos por vivienda, se observó que el 7.7% cuentan con un solo cuarto adicional a la cocina, baño y pasillos; 25.5% tienen dos y 66.8% tres o más cuartos (Figura 1.12). Al analizar esta distribución para cada una de las regiones de Nuevo León, se pudo observar que la Región Sur muestra los mayores porcentajes de viviendas que cuentan con uno o dos cuartos, 25.2% y 40.3% respectivamente, porcentaje que supera el promedio estatal, mientras que las regiones Metropolitana, Norte y Centro mostraron el mayor porcentaje de viviendas con tres o más cuartos: 71.4%, 55.8% y 47.4% respectivamente (Figura 1.13).

Figura 1.12 Hogares según número de cuartos.

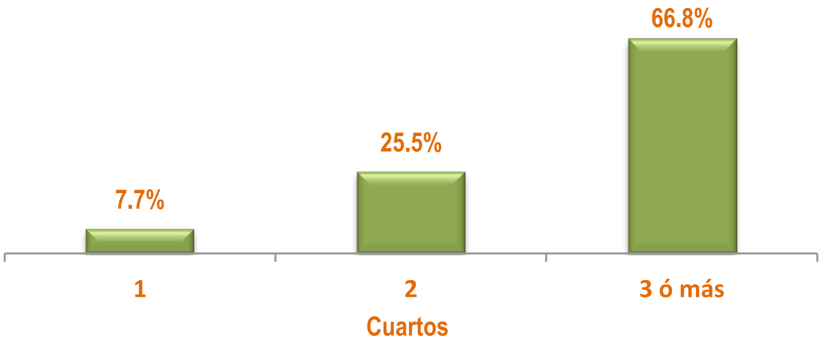

Fuente: EESN-NL 2011/2012 (Cuestionario Hogar).

Figura 1.13 Hogares según número de cuartos, divididos por región geográfica.

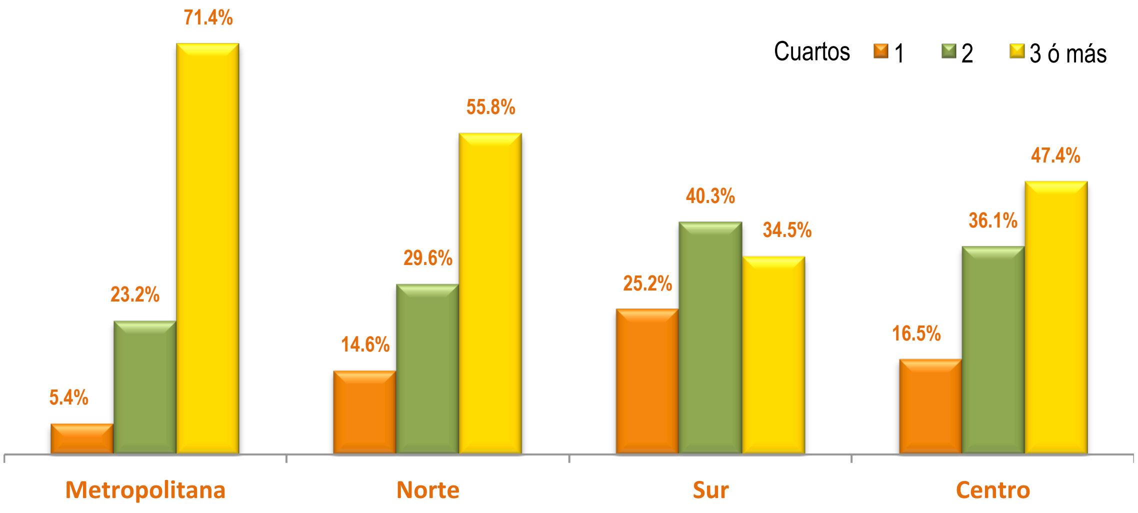

Fuente: EESN-NL 2011/2012 (Cuestionario Hogar).

En lo relativo a servicios básicos en la vivienda, el 99.8% de estas cuentan con energía eléctrica, el 96.0% tiene acceso a agua entubada (con toma domiciliaria), en el 1.9% su fuente principal es el pozo protegido o subterráneo, 2.2% concentran las que tienen como fuente el agua de lluvia almacenada en depósito o cisterna, la recogida directamente en una charca o arroyo y la abastecida por pipa. Según el tipo de drenaje de la vivienda, 89.9% refiere estar conectado a la calle, 6.0% están conectadas a una fosa séptica y 4.1% no tienen drenaje. Según el tipo de sanitarios de la vivienda 90.0% tienen desagüe al sistema de alcantarillado, 6.6% tienen desagüe a fosa séptica y 3.5% tienen algún tipo de letrina (Figura 1.14).

Figura 1.14 Distribución de viviendas según disposición de servicios.

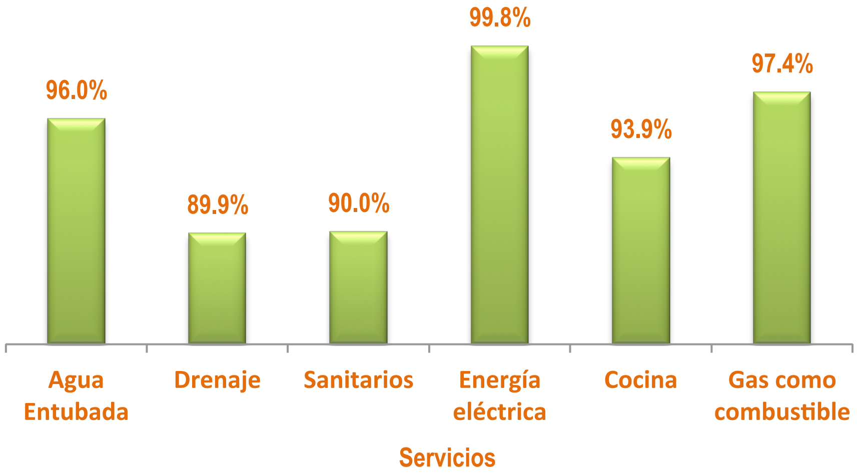

Fuente: EESN-NL 2011/2012 (Cuestionario Hogar).

En cuanto a la disposición de servicios básicos por regiones en las viviendas de Nuevo León, se observó que la Región Sur tiene el menor acceso a estos servicios, mostrando el porcentaje más bajo de disponibilidad de energía eléctrica 98.3%, sólo un 64.7% tiene acceso al agua entubada (con toma domiciliaria), 14.7% cuenta con drenaje conectado a la calle y 11.7% con sanitario conectado al sistema de desagüe de alcantarillado. Las regiones con mejor acceso a servicios básicos fueron Metropolitana, Norte y Centro del Estado (Tabla 1.7).

Tabla 1.7 Servicios Básicos en la vivienda, divididas por región geográfica.

Tipo de servicio	Región				Estatal
	Metropolitana %	Norte %	Sur %	Centro %	%
**Energía eléctrica**	99.9	99.6	98.3	100.0	99.8
**Agua**					
Entubada (c/toma domiciliaria)	98.7	89.0	64.7	87.0	96.0
Pozo protegido	0.2	6.8	8.6	12.8	2.2
Agua de lluvia (depósito o cisterna)	1.1	4.2	26.7	0.2	1.9
**Drenaje**					
Conectado a la calle	97.1	62.6	14.7	69.9	90.0
Fosa séptica, a un río, lago o barranca	1.2	33.6	25.9	22.1	6.0
No tiene drenaje	1.7	3.8	59.5	8.1	4.1
**Sanitario**					
Desagüe al sistema de alcantarillado	97.6	61.3	12.0	68.3	90.0
Desagüe a fosa séptica	1.4	34.0	30.8	24.5	6.6
Letrina	1.0	4.6	57.3	7.2	3.5

Fuente: EESN-NL 2011/2012 (Cuestionario Hogar).

Según el lugar donde cocinan, 93.9% de las familias mencionaron realizar esta actividad en una habitación o construcción independiente, 6.0% en la habitación donde duermen y 0.1% al aire libre.

La Región Centro mostró el menor porcentaje de población que cocina en habitación o construcción independiente: 88.2%, y por lo tanto el mayor porcentaje de población que cocina en la habitación donde se convive o se duerme: 11.8%. La Región Sur mostró el mayor porcentaje de población que cocina al aire libre: 1.7%.

El 97.4% utiliza el gas como combustible para cocinar y 2.6% electricidad, madera, carbón u otro (Tabla 1.8). En la Región Sur solo el 58.1% utiliza gas, mientras que 41.9% utiliza electricidad, carbón u otro tipo de combustible.

Tabla 1.8 Población según el lugar, tipo de combustible y tipo de estufa utilizados para cocinar, dividido por región geográfica.

Lugar	Región Metropolitana %	Norte %	Sur %	Centro %	Estatal %
**Lugar en donde cocina**					
Habitación independiente y construcción independiente	94.6	94.5	93.2	88.2	93.9
Habitación en la que se convive o se duerme y al aire libre	5.4	5.5	6.8	11.8	6.1
**Tipo de combustible utilizado para cocinar**					
Gas	98.3	97.8	58.1	97.9	97.4
Electricidad y otros*	1.7	2.3	41.9	2.1	2.6
**Tipo de estufa utilizado para cocinar**					
Estufa de gas	98.2	96.9	62.5	97.5	97.2
Horno cerrado con chimenea	0.2	0.9	11.5	0.2	0.5
Fuego abierto u horno con chimenea o campana	0.0	0.9	12.5	0.9	0.5
Fuego abierto u horno sin chimenea ni campana	0.2	0.4	7.3	1.1	0.5
Otro**	1.3	0.9	6.3	0.2	1.3

Fuente: EESN-NL 2011/2012 (Cuestionario Hogar).
* Incluye queroseno, carbón, carbón vegetal, madera, residuos agrícolas o de cultivos, estiércol animal, matojos o hierba.
** Incluye parrilla eléctrica, estufa de carbón.

Con respecto a la calefacción en los hogares se observó que 75.0% no la utiliza, y solo 25.0% usa algún tipo de calefacción como aparato de calefacción eléctrico o de gas (Figura 1.15).

Figura 1.15 Tipo de calefacción utilizado en los hogares.

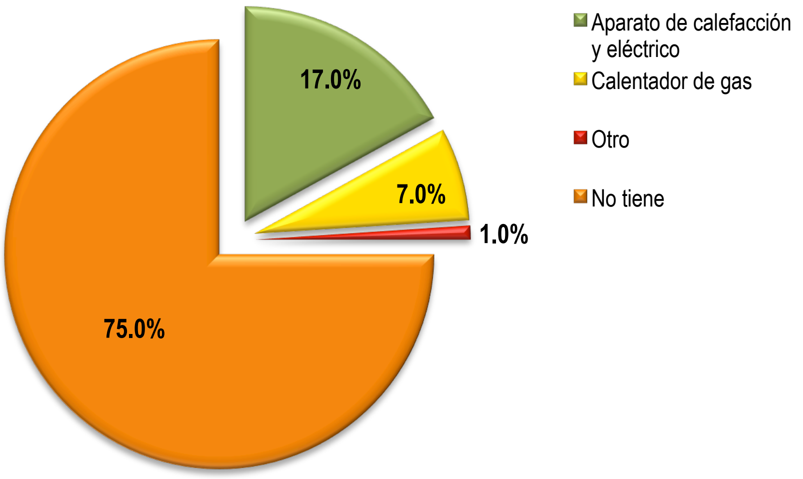

Fuente: EESN-NL 2011/2012 (Cuestionario Hogar).

Bienes o activos del hogar

De los 25 activos del hogar registrados, los de mayor porcentaje a nivel estatal fueron refrigerador con 95.2%, estufa de gas 94.2%, televisión a color 93.6%, licuadora 88.8%, lavadora 87.6% y otros electrodomésticos 87.3% como plancha y la batidora (Figura 1.16).

Figura 1.16 Familias según posesión de activos en el hogar.

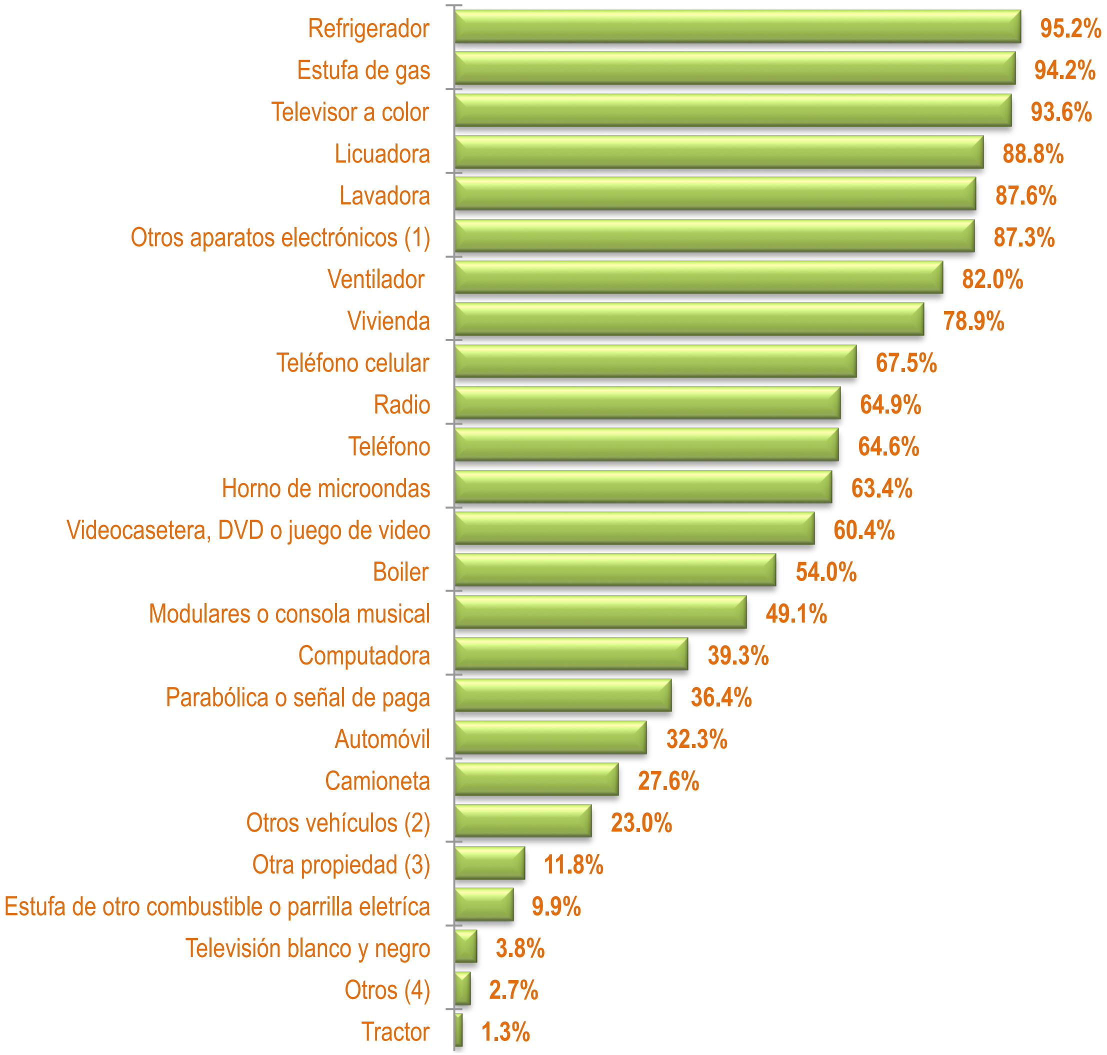

Fuente: EESN-NL 2011/2012 (Cuestionario Hogar).

(1) Incluye otros aparatos electrodomésticos como planchas y batidoras.
(2) Incluye motocicletas, motonetas y bicicletas.
(3) Incluye otra casa o construcción, inmueble, terreno o predio.
(4) Incluye otros bienes que en el hogar han sido considerados como indispensables.

Al realizar el análisis por región se encontró que los cinco activos con mayor presencia en el área metropolitana fueron refrigerador 95.7%, estufa de gas 94.7%, televisión a color 94.1%, licuadora 91.5% y otros aparatos electrónicos 89.1%; en la Región Norte fueron refrigerador 96.3%, estufa de

gas 94.6%, televisión a color 92.5%, ventilador 87.1% y lavadora 81.3%, estos mismos activos en el hogar fueron los cinco principales de la Región Centro con 96.1%, 95.9%, 94.0%, 91.3% y 86.0% respectivamente. Para la Región Sur los activos más presentes fueron vivienda 84.9%, televisión a color 78.2%, refrigerador 74.6%, estufa de gas 71.2% y lavadora 67.0%.

Respecto a los cinco activos que tuvieron menor presencia en cada una de las regiones fueron: para el área Metropolitana, Región Norte y Región Centro, otra propiedad como otra casa o construcción, inmueble, terreno o predio, con 11.6%, 10.4% y 14.5%, estufa de otro combustible o parrilla con 10.7%, 6.6% y 7.1%, televisión blanco y negro 3.8%, 3.8%, 2.4%, otros bienes considerados como indispensables en el hogar: 2.5%,1.3% y 5.4% y tractor con 1.1%, 2.5% y 2.2%. Para la Región Sur fueron televisión blanco y negro 8.4%, computadora 5.0%, estufa de otro combustible o parrilla 4.2%, tractor 2.5% y otros aparatos como planchas o batidoras 1.7% (Tabla 1.9).

Tabla 1.9. Activos del hogar, dividido por región geográfica.

Activos	Metropolitana	Norte	Sur	Centro
	%	%	%	%
Refrigerador	95.7	96.3	74.6	96.1
Estufa de gas	94.7	94.6	71.2	95.9
Televisión a color	94.1	92.5	78.2	94.0
Licuadora	91.5	80.4	56.3	82.3
Lavadora	89.0	81.3	67.0	86.0
Otros aparatos electrónicos (1)	89.1	79.3	65.6	84.5
Ventilador	82.3	87.1	28.6	91.3
Vivienda	79.1	78.3	84.9	76.0
Teléfono celular	69.8	58.6	24.4	66.5
Radio	66.4	60.4	38.1	62.6
Teléfono	70.9	39.2	17.8	42.4
Horno de microondas	66.3	55.8	25.2	55.8
Videocasetera, DVD o juego de video	66.0	37.8	13.6	43.3
Boiler	59.3	37.5	16.1	33.7
Modular o consola de sonido	52.9	28.2	26.9	37.0
Computadora	43.8	17.1	5.0	26.1
Parabólica o señal de paga	36.3	32.9	33.1	39.8
Automóvil	33.5	25.8	11.0	32.0
Camioneta	25.5	38.5	36.4	35.3
Otros vehículos (2)	22.1	23.8	17.7	30.7
Otra propiedad (3)	11.6	10.4	11.8	14.5
Estufa de otro combustible o parrilla	10.7	6.6	4.2	7.1
Televisión blanco y negro	3.8	3.8	8.4	2.4
Otros (4)	2.5	1.3	1.7	5.4
Tractor	1.1	2.5	2.5	2.2

Fuente: EESN-NL 2011/2012 (Cuestionario Hogar).
(1) Incluye otros aparatos electrodomésticos como planchas y batidoras.
(2) Incluye motocicletas, motonetas y bicicletas.
(3) Incluye otra casa o construcción, inmueble, terreno o predio.
(4) Incluye otros bienes que en el hogar han sido considerados como indispensables.

Destaca que en el Estado el 59.9% de los hogares refieren tener automóvil o camioneta, el porcentaje más alto lo tiene la Región Centro con un 67.3%.

En lo relacionado a las tecnologías de comunicación e información el 39.3% de la población en Nuevo León tiene computadora, en contraste con la Región Sur que registra un 5.0%. El 67.5% de la población del Estado tiene celular, siendo la Región Metropolitana la que cuenta con el mayor porcentaje con un 69.8% en comparación con el 24.4% de la Región Sur.

En cuanto a la forma de pago para adquirir los activos, de la población que cuenta con casa propia el 81.6% la pagó de contado, igualmente un 90.7% de la población que cuenta con automóvil o camioneta cubrió su costo de la misma manera.

Los activos del hogar que una mayor parte de la población se encuentra pagándolos son antena parabólica o televisión por cable con un 32.4%, teléfono 24.5%, otra casa, construcción o inmueble 22.9% y casa propia con un 13.3% de la población.

En datos proporcionados por el INEGI en el año 2005 a nivel nacional se cita que 20.0% de las viviendas poseía computadora, 63.0% lavadora, 79.0% refrigerador y 91.0% televisor, mientras que para el año 2011 este mismo instituto a través de las Estadísticas sobre Disponibilidad y Uso de Tecnología, menciona que 44.0% de la población mexicana tiene teléfono fijo, 30.3% computadora, 30.4% TV de paga y 23.3% internet.

Resultados relevantes

- El grupo de 0 a 14 años es el que más población concentra.
- El promedio de habitantes por hogar es de 3.7 individuos por vivienda.
- El 52.9% de familias tienen mascota y el perro representa el 41.9%.
- De los niños de 6 a 14 años 98.5% sí asisten a la escuela.
- Se encuentra trabajando el 41.3% de la población.
- El 68% de las viviendas en Nuevo León cuentan con tres o más habitaciones.
- Cuentan con energía eléctrica 99.8% de las viviendas; más del 90.0% cuentan con servicios básicos de gas, agua y drenaje.
- Los cinco bienes más comunes en los hogares de Nuevo León son: refrigerador, estufa de gas, televisor a color, licuadora y lavadora.
- El 67.5% tienen celulares y el 39.3% computadoras.
- El 32.3% cuentan con automóvil.
- En la Región Sur y Metropolitana se acentúa la distribución de la población en el grupo de 10 a 19 años de edad.
- En la Región Metropolitana el número de habitantes por vivienda es de tres personas, mientras que en el resto de las regiones (Norte, Sur y Centro) es de 2.
- La Región Sur tiene el menor porcentaje de hogares con jefatura de mujeres, con un 14.5% en comparación con el promedio estatal que es de 21.7%.
- En lo referente al estado civil se presenta la estadística en forma contrastante: en la Región Norte el porcentaje de unión libre es de 9.5% en comparación al porcentaje estatal que es de 6.1%.
- Según mascotas presentes, en las viviendas de la Región Sur, el 63% tienen perros, porcentaje superior al estatal que es de 41.9%. Mientras que en la Región Norte el 7.5% tienen gatos, porcentaje superior al estatal que es de 3.3%.
- Respecto a los activos en las viviendas, el refrigerador es el activo con mayor presencia para todas las regiones, mientras que los teléfonos celulares y los videojuegos son los activos con menor presencia en la Región Sur.

Capítulo 2

Salud

EESN – NL 2011/2012

2. Salud

La salud y la enfermedad son el resultado de un proceso de interacción permanente del ser humano con el medio en que vive, tratando de adaptarse a éste; obedecen a los mismos factores externos e internos, salud es adaptación y equilibrio entre ambos, enfermedad es desadaptación [Álvarez, 2012]. Numerosos factores son los que intervienen en la salud y la enfermedad como: los físicos del medio, biológicos, psicológicos, culturales y sociales; y muy variadas las respuestas del organismo. La EESN-NL 2011/2012 muestra la prevalencia de las enfermedades que afectan a la población de Nuevo León, como diabetes, hipertensión arterial, cáncer, daños a la salud por accidentes, y otros factores de riesgo como el consumo de tabaco y alcohol, sin pasar por alto la actividad física.

Enfermedad diarreica aguda

La prevalencia de enfermedades diarreicas agudas (EDA's) en eventos en semanas previas a la encuesta en menores de cinco años, fueron evaluadas en una muestra de 567 niños que representan a 146,469 menores en el Estado. La prevalencia de EDA's en Nuevo León fue del 5.1%, prevalencia menor a lo reportado por la ENSANUT 2012 (11.0%) para el mismo grupo de edad. En las regiones Norte y Sur la prevalencia fue de 10.0% y 10.5% respectivamente, mientras que en las regiones Metropolitana y Centro la prevalencia fue de 4.5% y 5.5% respectivamente (Tabla 2.1).

Tabla 2.1 Niños menores de 5 años con presencia de diarrea en las últimas dos semanas previas a la encuesta, divididos por región geográfica.

Presencia de diarrea	Región				Estatal
	Metropolitana %	Norte %	Sur %	Centro %	%
Sí	4.5	10.0	10.5	5.5	5.1
No	95.5	90.0	89.5	94.5	94.9
Total	100.0	100.0	100.0	100.0	100.0

Fuente: EESN-NL 2011/2012 (Cuestionario Niños de 0 a 9 años).

La EESN-NL 2011/2012 identificó que del total de niños menores de cinco años que registraron un evento de diarrea en las dos semanas previas a la encuesta, los líquidos mayormente consumidos durante la presencia de diarrea fueron Vida Suero Oral con 52.2%, agua de frutas 52.0%, agua sola 48.0%, agua de arroz, caldos y/o sopas 20.0%. En las regiones Norte, Sur y Centro los líquidos

mayormente consumidos son agua sola con un porcentaje de consumo de 60.0% a 85.7%, seguido de Vida Suero Oral, comercial o casero de 40.0% a 85.7%, mientras que en la Región Metropolitana se consume más agua de fruta 69.4%, seguido de vida suero oral 47.2% (Tabla 2.2). Los motivos de consulta de mayor frecuencia en las EDA's fueron: las evacuaciones frecuentes 78.2%, seguido de fiebre 25.2% y vómitos 18.2%. A nivel regional el comportamiento fue similar (Tabla 2.3).

Tabla 2.2 Niños menores de 5 años según líquidos administrados* durante la presencia de diarrea, dividido por región geográfica.

Líquidos administrados	Región				Estatal
	Metropolitana	Norte	Sur	Centro	
	%	%	%	%	%
Vida Suero Oral, suero comercial y suero casero	47.2	40.0	50.0	85.7	52.2
Agua sola	38.9	60.0	50.0	85.7	48.0
Agua de frutas	69.4	20.0	0.0	0.0	52.0
Agua de arroz	27.8	0.0	0.0	0.0	20.0
Caldos/sopas	25.0	0.0	0.0	14.3	20.0
Refresco	0.0	0.0	0.0	28.6	4.0
Ninguno	16.7	0.0	0.0	0.0	12.0

Fuente: EESN-NL 2011/2012 (Cuestionario Niños de 0 a 9 años).
*En la encuesta también fueron incluidos otros líquidos (té, atole, leche, leche rebajada, café y otros), que no fueron referenciados por los encuestados.

Tabla 2.3 Madres de niños menores de 5 años según la percepción del motivo de consulta por diarrea, dividido por región geográfica.

Signos y síntomas	Región				Estatal
	Metropolitana	Norte	Sur	Centro	
	%	%	%	%	%
Mucha sed	14.7	20.0	23.5	15.9	15.4
Poca ingesta de líquidos o alimentos	17.7	20.0	29.4	15.9	18.0
Estar muy quieto (débil)	14.2	20.0	35.3	17.5	15.6
Evacuaciones frecuentes	80.5	72.0	64.7	69.8	78.2
Vómitos	15.7	32.0	17.6	28.6	18.2
Fiebre	22.8	28.0	23.5	39.7	25.2
Llanto sin lágrimas	11.1	4.0	17.6	7.9	10.6

Fuente: EESN-NL 2011/2012 (Cuestionario Niños de 0 a 9 años).

Infecciones respiratorias agudas

Con respecto a las infecciones respiratorias agudas (IRA's), los datos fueron obtenidos de una muestra de 1,368 menores de 10 años de edad, que representan a una población de 355,719 niños. La prevalencia estatal de IRA's dos semanas previas a la entrevista fue de 37.5%, prevalencia menor a la reportada en ENSANUT 2012 que fue de 41.0%; a nivel regional la prevalencia fue de 20.2% a 41.1%, donde la Región Metropolitana mostró el mayor valor e igual a la media nacional (Tabla 2.4).

Tabla 2.4 Niños de 0 a 9 años según presencia de infección respiratoria aguda durante las dos últimas semanas previas a la encuesta, dividido por región geográfica.

Infección Respiratoria	Región				Estatal
	Metropolitana	Norte	Sur	Centro	
	%	%	%	%	%
Si	41.1	25.4	23.6	20.2	37.3
No	58.8	74.6	76.3	79.7	62.6
Total	100.0	100.0	100.0	100.0	100.0

Fuente: EESN-NL 2011/2012 (Cuestionario Niños de 0 a 9 años).

La detección de signos de alerta, referidos por las madres en los niños ante la presencia de IRA's fueron: la fiebre por más de tres días (39.1%), seguido de "se ve más enfermo" (28.0%) y "no come ni bebe" el (21.8 %). Estos signos de alerta son los mayormente identificados a nivel regional (Tabla 2.5).

Tabla 2.5 Madres de niños de 0 a 9 años según la percepción del motivo de consulta por infección respiratoria aguda dividido por región geográfica.

Signos y Síntomas	Región				Estatal
	Metropolitana	Norte	Sur	Centro	
	%	%	%	%	%
Respiración rápida	10.5	5.1	18.6	15.5	0.0
No puede respirar	19.0	20.3	24.0	13.6	23.6
No come, ni bebe	21.0	15.2	37.3	21.3	21.8
Se pone frío	3.2	5.1	16.0	2.6	3.6
Se ve más enfermo	27.0	30.4	29.3	27.2	28.0
Más de tres días con fiebre	37.8	35.5	29.3	42.7	39.1
Presencia de pus en oídos	2.5	1.7	0.0	1.3	2.2
Puntos blancos en la garganta	8.0	23.7	8.0	12.9	9.3
Otros	46.3	32.1	34.6	29.7	43.3

Fuente: EESN-NL 2011/2012 (Cuestionario Niños de 0 a 9 años).

Lactancia materna

La lactancia materna es la forma ideal de aportar a los niños menores de dos años los nutrientes necesarios para asegurar un crecimiento y desarrollo saludable. El 80.2% de los niños menores de 2 años son alimentados al seno materno, porcentaje similar en cada una de las regiones con valores desde 75.0% a 87.5% (Figura 2.11)

Figura 2.11 Niños menores de 2 años según lactancia materna.

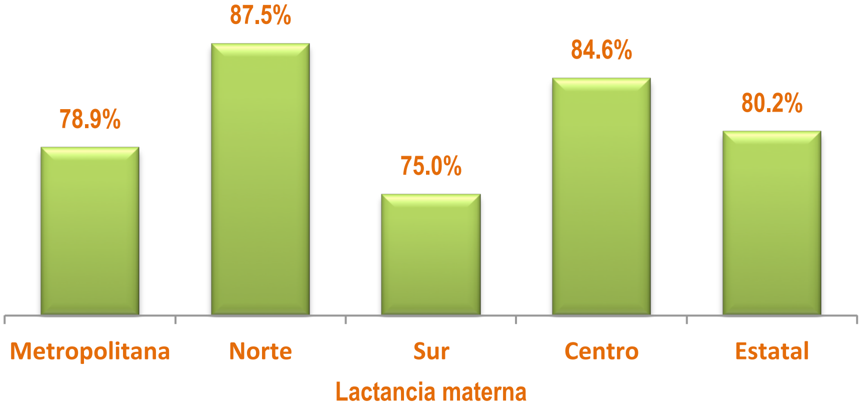

Fuente: EESN-NL 2011/2012 (Cuestionario Niños de 0 a 9 años).

Respecto al tiempo de duración de lactancia materna en niños menores de 2 años, se observa que 37.1% es de 0 a 3 meses de edad, 29.2% de 4 a 6 meses de edad y el 33.7% es mayor o igual a 7 meses de edad. (Figura 2.12). Según región geográfica, la Región Metropolitana muestra el mayor porcentaje de práctica de lactancia materna en periodo de edad de 0 a 3 meses con 40.3%, en la Región Centro el mayor valor es de 60.0% en la edad de 4 a 6 meses, mientras que en las regiones Norte y Sur el 50% de los menores de dos años tienen lactancia materna igual o mayor a 7 meses de edad (Tabla 2.32).

Figura. 2.12 Niños menores de 2 años según duración de lactancia materna.

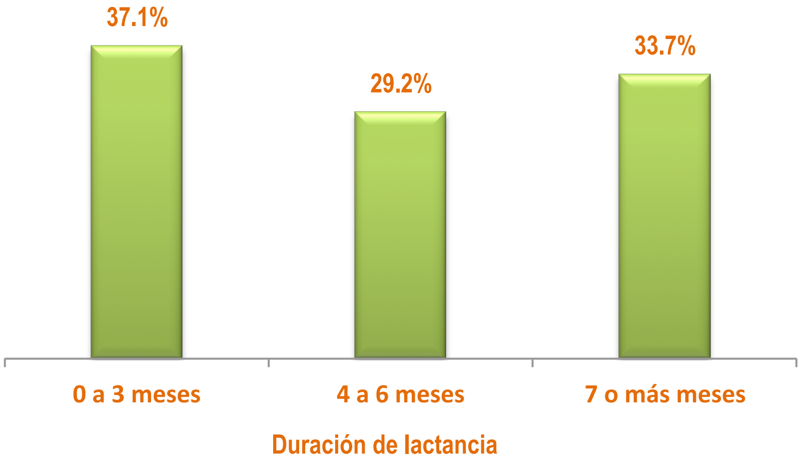

Fuente: EESN-NL 2011/2012 (Cuestionario Niños de 0 a 9 años).

Tabla 2.32 Tiempo de duración de lactancia materna en niños menores de 2 años, divididos por región geográfica.

Duración	Región				Estatal
	Metropolitana %	Norte %	Sur %	Centro %	%
0 a 3 meses	40.3	37.5	25.0	20.0	37.1
4 a 6 meses	26.9	12.5	25.0	60.0	29.2
7 o más meses	32.8	50.0	50.0	20.0	33.7
Total	100.0	100.0	100.0	100.0	100.0

Fuente: EESN-NL 2011/2012 (Cuestionario Niños de 0 a 9 años).

Vacunación

La EESN - NL 2011/2012 obtuvo datos de vacunación de una población de 556 niños menores de 5 años, los cuales representan a una población de 145,020 individuos de ese rango de edad, de ellos el 97.0% refiere tener cartilla, de los cuales el 73.3% mostró la cartilla de vacunación. (Figura. 2.6). Los mayores porcentajes de esquema completo se presentan en los niños de 4 años (Figura 2.7).

Figura 2.6 Niños menores de 5 años que poseen cartilla de vacunación.

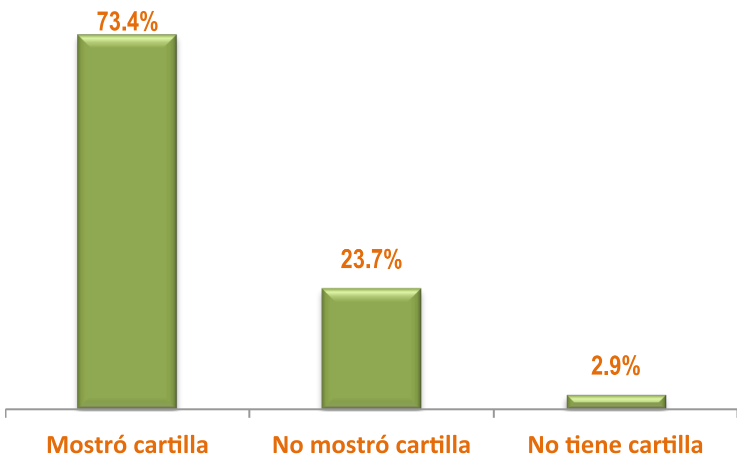

Fuente: EESN-NL 2011/2012 (Cuestionario Niños de 0 a 9 años).

Figura 2.7 Esquema completo de vacunación en menores de 5 años.

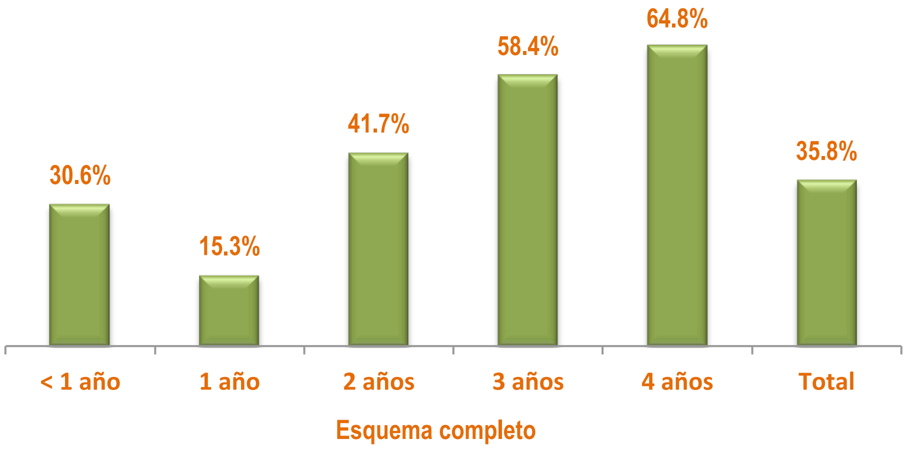

Fuente: EESN-NL 2011/2012 (Cuestionario Niños de 0 a 9 años).

Regionalmente y según grupo etario, se observa que en los esquemas por su estructura de aplicación se incrementa el porcentaje según la edad, en los menores de un año, la Región Centro muestra los porcentajes más altos de esquemas completos de vacunación en los primeros dos años de vida, a partir de los tres años en la Región Metropolitana más del 70.0% de los menores de cinco años cuentan con esquema completo de vacunación (Figura 2.8).

Figura 2.8. Esquemas de vacunación según edad en menores de 5 años, divididos según región geográfica.

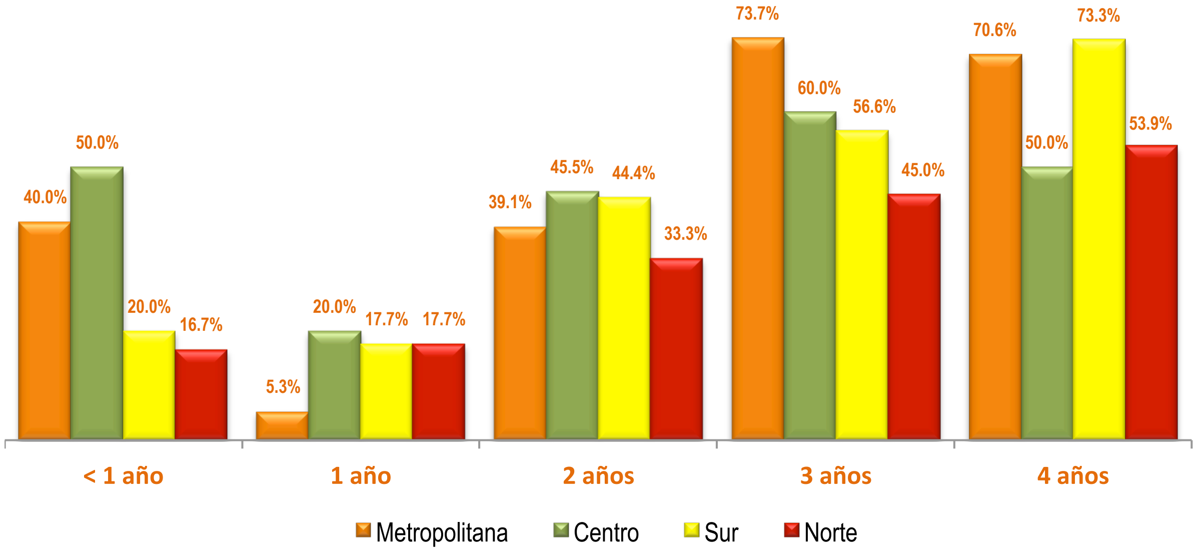

Fuente: EESN-NL 2011/2012 (Cuestionario Niños de 0 a 9 años).

Actividad física

Para conservar el balance energético que conduce a un peso saludable, la actividad física es un componente primordial de la ecuación, por lo tanto, investigar los hábitos de actividad física fue una parte fundamental de la EESN-NL 2011/2012.

El cuestionario de actividad física permitió captar información sobre las horas semanales en que los individuos realizaban actividad física o permanecían inactivos.

Se preguntó sobre: a) la actividad física vigorosa, definida como actividades que requieren un gasto de energía equivalente a cinco MET/hora (unidad de gasto energético por actividad física), es decir, que demandan un gasto de energía considerable, incluyendo actividades como jugar futbol, basquetbol, voleibol, karate o artes marciales, conducir bicicleta, patinar o usar patineta, bailar o tomar clases de baile, correr, hacer gimnasia, aeróbics o ballet, nadar y otros juegos o deportes, o actividades en las que es necesario correr o agitarse; b) actividades moderadas que requieren un gasto menor de energía, como limpiar o arreglar la casa, caminar (incluso a la escuela) cargando cosas en el campo; c) actividades sedentarias que requieren un gasto menor de energía: horas en que se ve televisión, películas, videos o se usan videojuegos. Se investigó sobre el tiempo dedicado a cada una de estas categorías de lunes a viernes y durante el fin de semana.

Los grupos de edad evaluados fueron: 761 menores (5-9 años), que representan a 222,617 individuos; 1,319 adolescentes (10-19 años) que representan una población de 464,751 individuos; 3,125 adultos (20-59 años) que representan a 956,176 individuos y 1,475 adultos mayores (60 años y más) que representan a una población de 362,412 individuos.

De todos los grupos de edad evaluados en cuanto a su actividad física semanal, del 66.1% al 85.8% no realiza ningún tipo de actividad física vigorosa, del 39.8% al 74.1% no realiza actividad física moderada, del 39.8% al 74.1% no realiza actividad física ligera (Figura 2.1). El comportamiento en cuanto actividad física por regiones es similar al estatal (Tablas 2.6, 2.7, 2.8 y 2.9).

Figura 2.1 Tipo de actividad física y frecuencia por grupo de edad.

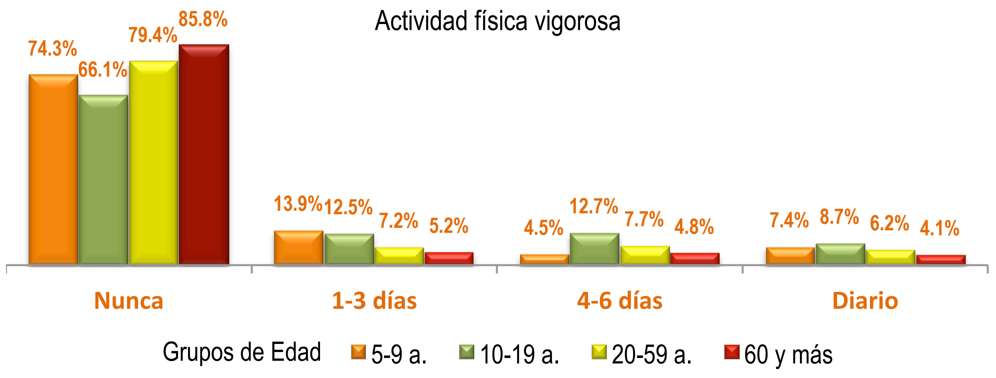

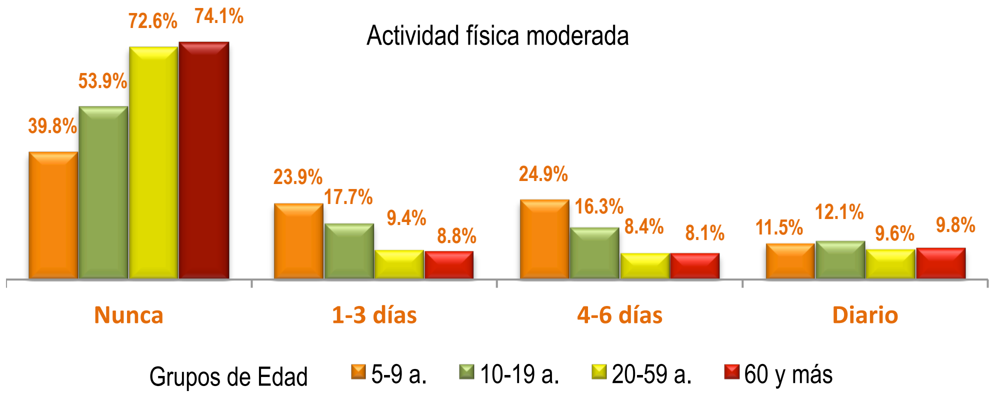

Figura 2.1 Tipo de actividad física y frecuencia por grupo de edad. (Continuación)

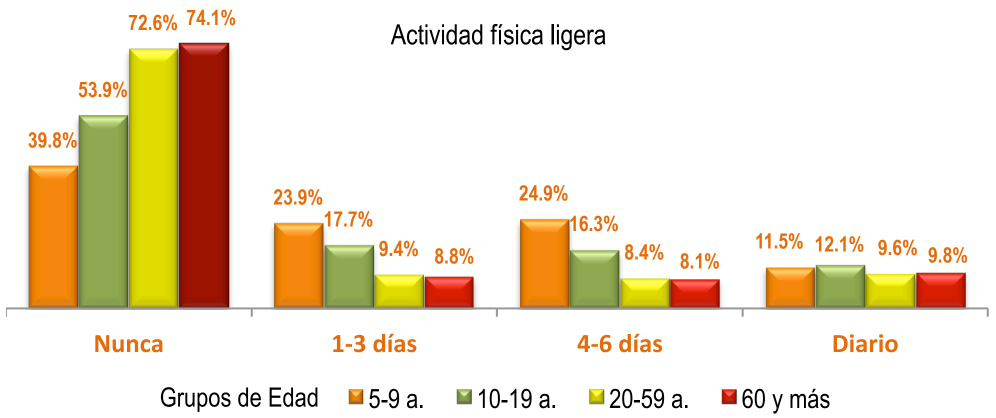

Fuente: EESN-NL 2011/2012 (Cuestionario Niños de 0 a 9, 10 a 19, 20 a 59 y 60 años y más).

Tabla 2.6 Tipo de actividad física y frecuencia en niños de 5 a 9 años, dividido por región geográfica.

Tipo de actividad física	Días	Región				Estatal
		Metropolitana	Norte	Sur	Centro	
		%	%	%	%	%
Vigorosa	1 a 3	14.9	6.0	5.0	11.7	13.8
	4 a 6	2.9	9.1	5.0	12.7	4.6
	Diario	6.4	18.2	15.0	8.4	7.4
	Nunca	75.8	66.7	75	67.2	74.2
	Total	100.0	100.0	100.0	100.0	100.0
Moderada	1 a 3	23.7	25.0	20.0	24.2	23.8
	4 a 6	24.9	21.8	10.0	28.4	24.9
	Diario	9.9	12.5	40.0	14.7	11.4
	Nunca	41.5	40.7	70.0	32.7	39.9
	Total	100.0	100.0	100.0	100.0	100.0
Ligera	1 a 3	15.5	3.1	5.0	12.5	14.3
	4 a 6	43.5	59.4	30.0	34.4	42.7
	Diario	26.4	21.9	60.0	38.5	28.7
	Nunca	14.6	15.6	5.0	14.6	14.3
	Total	100.0	100.0	100.0	100.0	100.0

Fuente: EESN-NL 2011/2012 (Cuestionario Niños de 0 a 9 años).

Tabla 2.7 Tipo de actividad física y frecuencia en adolescentes de 10 a 19 años, dividido por región geográfica.

Tipo de actividad física	Días	Región				Estatal
		Metropolitana %	Norte %	Sur %	Centro %	%
Vigorosa	1 a 3	12.8	10.3	4.6	8.0	12.0
	4 a 6	12.6	12.1	4.6	10.3	12.2
	Diario	7.8	6.9	22.7	11.0	8.3
	Nunca	66.8	70.7	68.1	70.7	67.5
	Total	100.0	100.0	100.0	100.0	100.0
Moderada	1 a 3	16.8	17.3	9.2	21.9	17.2
	4 a 6	15.8	13.8	13.7	18.3	16.0
	Diario	10.4	15.5	45.5	16.1	11.8
	Nunca	57.0	53.4	31.6	43.7	55.0
	Total	100.0	100.0	100.0	100.0	100.0
Ligera	1 a 3	13.3	12.1	9.2	12.3	13.0
	4 a 6	36.2	32.8	18.2	28.3	34.9
	Diario	35.7	31.0	63.6	34.8	35.9
	Nunca	14.8	24.1	9.0	24.6	16.2
	Total	100.0	100.0	100.0	100.0	100.0

Fuente: EESN-NL 2011/2012 (Cuestionario Adolescentes de 10 a 19 años)

Tabla 2.8 Tipo de actividad física y frecuencia en adultos de 20 a 59 años, dividido por región geográfica.

Tipo de actividad física	Días	Región				Estatal
		Metropolitana %	Norte %	Sur %	Centro %	%
Vigorosa	1 a 3	7.1	4.4	9.3	4.3	6.4
	4 a 6	7.8	5.5	8.4	6.7	7.2
	Diario	4.5	5.6	35.5	6.1	13.4
	Nunca	80.6	84.5	46.8	82.9	73.0
	Total	100.0	100.0	100.0	100.0	100.0
Moderada	1 a 3	9.9	6.4	13.8	8.6	9.9
	4 a 6	8.1	10.2	16.2	11.0	11.5
	Diario	7.0	11.0	48.2	10.0	19.7
	Nunca	75.0	72.4	21.8	70.4	58.9
	Total	100.0	100.0	100.0	100.0	100.0
Ligera	1 a 3	16.9	11.2	9.9	12.7	12.7
	4 a 6	26.3	25.1	23.3	26.8	25.3
	Diario	32.1	26.5	59.9	31.8	38.1
	Nunca	24.7	37.2	6.9	28.7	23.9
	Total	100.0	100.0	100.0	100.0	100.0

Fuente: EESN-NL 2011/2012 (Cuestionario Adultos de 20 a 59 años).

Tabla 2.9 Tipo de actividad física y frecuencia en adultos de 60 años y más, dividido por región geográfica.

Tipo de actividad física	Días	Región				Estatal
		Metropolitana %	Norte %	Sur %	Centro %	%
Vigorosa	1 a 3	6.3	0.8	4.4	2.0	5.1
	4 a 6	5.5	3.3	4.4	2.5	4.9
	Diario	4.0	3.3	15.6	2.9	4.1
	Nunca	84.2	92.6	75.6	92.6	85.9
	Total	100.0	100.0	100.0	100.0	100.0
Moderada	1 a 3	9.9	3.3	6.6	6.8	8.8
	4 a 6	8.4	8.3	15.5	5.3	8.1
	Diario	7.7	12.4	26.7	10.1	9.0
	Nunca	74.0	76.0	51.2	77.8	74.1
	Total	100.0	100.0	100.0	100.0	100.0
Ligera	1 a 3	17.8	9.1	13.3	11.2	16.1
	4 a 6	21.0	16.6	26.6	18.3	20.4
	Diario	21.9	32.2	46.7	30.4	24.7
	Nunca	39.3	42.1	13.4	40.1	38.8
	Total	100.0	100.0	100.0	100.0	100.0

Fuente: EESN-NL 2011/2012 (Cuestionario Adultos de 60 años y más).

Diabetes

En la EESN-NL 2011/2012 se entrevistó a 3,072 personas que representan a 938,894 individuos de 20 a 59 años de edad y a 1,466 personas que representan a 360,088 individuos del grupo de 60 años y más. La prevalencia de diabetes tipo 2 en el grupo de 20 a 59 años, quienes conocían padecer diabetes tipo 2, es 8.8%. En el grupo de 60 años y más, la prevalencia de diabetes conocida es de 27.4%. Tomando en cuenta a ambos grupos para la prevalencia DMT2 en individuos mayores a 20 años, la prevalencia es de 14.6% en el Estado (Tabla 2.10).

Tabla 2.10 Prevalencia de DMT2 en individuos de 20 años y más.

Prevalencia	20-59 años %	60 años y más %	Total %
Diabetes Mellitus tipo 2	8.8	27.4	14.6
Sin Diabetes Mellitus tipo 2 conocida	91.2	72.6	85.4
Total	100.0	100.0	100.0

Fuente: EESN-NL 2011/2012 (Cuestionario Adultos de 20 a 59 años).

Tabla 2.11 Prevalencia de DMT2 en individuos mayores de 20 años y más, divididos por región geográfica.

Diagnóstico de diabetes	Región				Estatal
	Metropolitana	Norte	Sur	Centro	
	%	%	%	%	%
Adultos de 20 a 59 años					
Sí	8.5	7.5	4.7	12.3	8.8
No	91.5	92.5	95.3	87.7	91.2
Total	100.0	100.0	100.0	100.0	100.0
Adultos de 60 años y más					
Sí	29.4	24.2	17.8	20.5	27.4
No	70.6	75.8	82.2	79.5	72.6
Total	100.0	100.0	100.0	100.0	100.0

Fuente: EESN-NL 2011/2012 (Cuestionario Adultos de 20 a 59 años).

La prevalencia de DMT2 conocida según regiones geográficas en Nuevo León, en el grupo de 20 a 59 años, los más altos porcentajes se observan en las regiones Metropolitana y Centro con 8.5% y 12.3% respectivamente; y en el grupo de 60 años y más la prevalencia es mayor en las regiones Metropolitana y Norte con 29.4% y 24.2% respectivamente (Tabla 2.11).

Según el tiempo de evolución de la DMT2 de la población en los grupos de edad de 20 a 59 años y 60 años y más, es de 71.5% y 53.7% respectivamente (Figura 2.2).

Figura 2.2 Tiempo de evolución de DMT2 por grupo de edad.

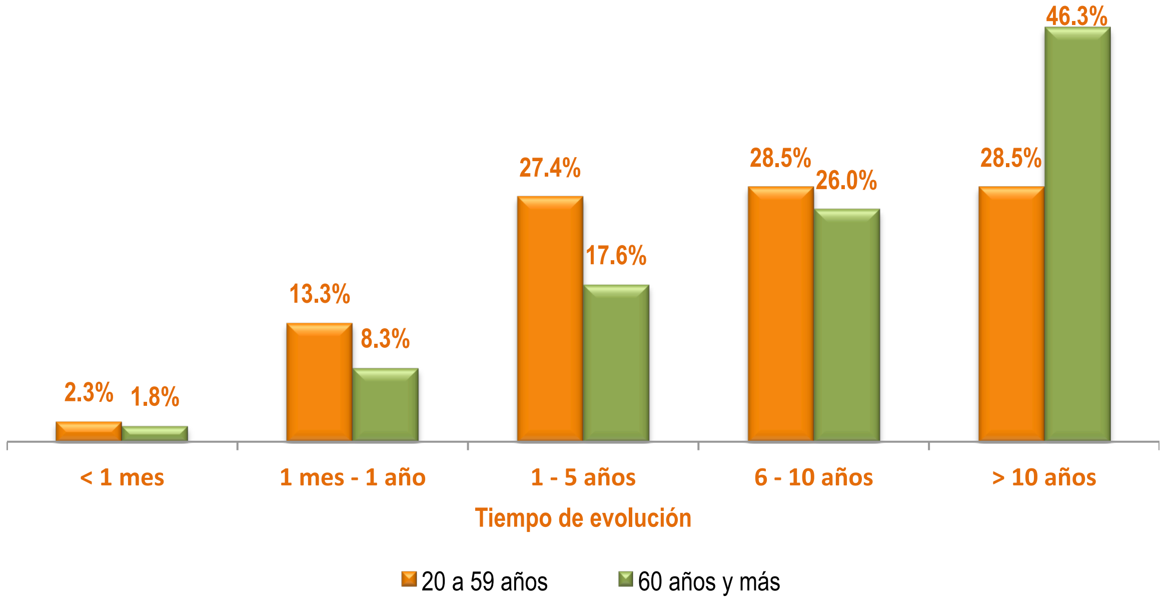

Fuente: EESN-NL 2011/2012 (Cuestionario Adultos de 20 a 59 años y 60 años y más).

En el grupo de 20 a 59 años 74.3% utiliza antidiabéticos orales (ADO) como tratamiento, 19.4% insulina, 0.8% la combinación de ADO + insulina y 5.5% no hacen uso de tratamiento farmacológico. Mientras que en el grupo de 60 años y más, 79.8% utiliza antidiabéticos orales (ADO) como tratamiento, 13.7 insulina, 3.9% la combinación de ADO + insulina y 2.6% no hacen uso de tratamiento farmacológico; a nivel regional el tratamiento para la diabetes es similar al comportamiento estatal, ya que se observa que en ambos grupos de edad más del 70.0% de la población utiliza los antidiabéticos orales (Tabla 2.12).

Tabla 2.12 Adultos de 20 años y más con diabetes según tratamiento farmacológico, dividido por región geográfica.

Tratamiento farmacológico	Región				Estatal
	Metropolitana	Norte	Sur	Centro	
	%	%	%	%	%
Adultos de 20 a 59 años					
Insulina	18.4	12.2	6.9	26.6	19.4
Pastillas	75.2	78.6	80.2	68.8	74.3
Ambas	0.2	8.0	9.0	0.5	0.8
No/Ninguno	6.2	1.2	3.9	4.1	5.5
Total	100.0	100.0	100.0	100.0	100.0
Adultos de 60 años y más					
Insulina	13.1	10.2	18.5	13.3	13.7
Pastillas	76.2	84.1	80.2	78.3	79.8
Ambas	4.8	4.5	0.0	6.0	3.9
Ninguno	5.9	1.1	1.2	2.4	2.6
Total	100.0	100.0	100.0	100.0	100.0

Fuente: EESN-NL 2011/2012 (Cuestionario Adultos de 20 a 59 años y 60 años y más).

Hipertensión

Para determinar la prevalencia del diagnóstico previo de HTA en la EESN-NL 2011/2012 se preguntó a 3,116 individuos del grupo de edad de 20 a 59 años, quienes representan a 953,729 individuos, encontrando una prevalencia de 13.0%. En el grupo de 60 años y más, se cuestionó a 1,471

personas que representan a una población de 361,387 personas en donde la prevalencia fue de 45.5%. La prevalencia estatal de HTA en mayores de 20 años de edad fue de 23.4% (Tabla 2.13).

La prevalencia de hipertensión arterial por región geográfica en adultos de 20 a 59 años muestra valores entre 12.8% y 13.9%, mientras que en el grupo de 60 años y más la prevalencia es mayor a 40.0%, siendo las regiones Metropolitana y Norte las que muestran valores superiores, 46.6% y 45.8% respectivamente (Tabla 2.14).

Tabla 2.13 Prevalencia de hipertensión arterial en adultos de 20 años y más.

Prevalencia	20-59 años<br>%	60 años y más<br>%	Total<br>%
Hipertensión arterial	13.0	45.5	23.4
Sin hipertensión arterial conocida	87.0	54.5	76.6
Total	100.0	100.0	100.0

Fuente: EESN-NL 2011/2012 (Cuestionario Adultos de 20 a 59 años y 60 años y más).

Tabla 2.14 Adultos de 20 años y más según diagnóstico de hipertensión arterial, dividido por región geográfica.

Diagnóstico de hipertensión	Región				Estatal
	Metropolitana<br>%	Norte<br>%	Sur<br>%	Centro<br>%	%
Adultos de 20 a 59 años					
Si	12.8	13.9	13.4	13.7	13.0
No	87.2	86.1	86.6	86.3	87.0
Total	100.0	100.0	100.0	100.0	100.0
Adultos de 60 años y más					
Sí	46.6	45.8	35.6	41.0	45.5
No	53.4	54.2	64.4	59.0	54.5
Total	100.0	100.0	100.0	100.0	100.0

Fuente: EESN-NL 2011/2012 (Cuestionario Adultos de 20 a 59 años y 60 años y más).

El 72.1% de los hipertensos en el grupo de edad de 20 a 59 años reciben tratamiento farmacológico, y las regiones Sur y Centro muestran los porcentajes más altos de personas con hipertensión que reciben tratamiento, 87.5% y 82.2% respectivamente. En el grupo de 60 años y más el 95.2% recibe tratamiento, porcentaje similar observado en cada una de las regiones del Estado (Tabla 2.15).

Tabla 2.15 Adultos de 20 a 59 años con hipertensión arterial según tratamiento farmacológico, dividido por región geográfica.

Tratamiento	Región				Estatal
	Metropolitana	Norte	Sur	Centro	
	%	%	%	%	%
Adultos de 20 a 59 años					
Sí	69.8	77.3	87.5	82.2	72.1
No	30.2	22.7	12.5	17.8	27.9
Total	100.0	100.0	100.0	100.0	100.0
Adultos de 60 años					
Sí	96.6	95.3	93.8	95.6	95.2
No	3.4	4.7	6.3	4.4	4.8
Total	100.0	100.0	100.0	100.0	100.0

Fuente: EESN-NL 2011/2012 (Cuestionario Adultos de 20 a 59 años y 60 años y más).

La prevalencia de HTA encontrada en adultos de 20 años y más fue de 21.0%; si tomamos en cuenta la clasificación por grados, la distribución es la siguiente: HTA grado I fue de 13.3%, grado II 5.5% y grado III 0.8%; hipertensión sistólica aislada: 1.4%.

Respecto a la prevalencia de hipertensión arterial detectada durante la encuesta, encontramos que de la población con edades entre 20 y 59 años, se evaluó con toma de tensión arterial a un total de 2,003 personas que refirieron no padecer hipertensión arterial y que representan a 613,153 personas, de éstas, se detectó hipertensión arterial en un 16.9%, que representan a una población de 103,765 personas. El 83.1% no tienen hipertensión, encontrando la mayoría, un 44.2%, con presión normal, y un 38.8 % con tensión arterial óptima.

Con respecto al grupo etario de 60 años y más, se encuestaron 603 personas que refirieron no padecer de hipertensión arterial y que representan a un total de 140,464 personas. En este grupo se encontró hipertensión arterial en 29.4%, y con ausencia de hipertensión 70.6% de las personas, dentro de la cuales el 30.1% tiene una tensión arterial óptima y un 40.4% se encuentra en lo normal (Figura 2.3).

Figura 2.3 Porcentaje de sujetos con diagnóstico de hipertensión arterial durante la encuesta por grupo de edad.

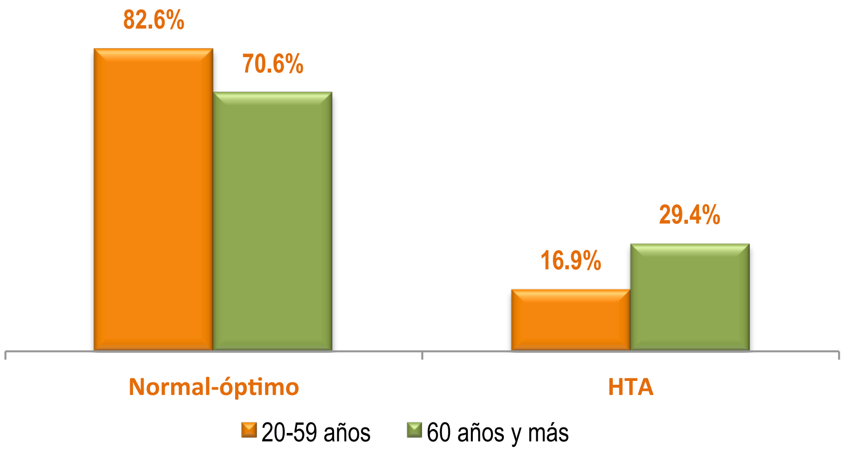

Fuente: EESN-NL 2011/2012 (Cuestionario Adultos de 20 a 59 años y 60 años y más)

La prevalencia de hipertensión arterial en la EESN - NL 2011/2012, teniendo en cuenta los casos ya diagnosticados más lo casos encontrados, nos muestran un prevalencia de hipertensión arterial en el grupo de 20 a 59 años de edad del 29.9% (13.0% con diagnóstico previo y 16.9% con la medición al momento de la encuesta); y en el grupo de 60 años y más, de 74.8% (45.5% ya diagnosticados y 29.4% con la medición al momento de la encuesta). La prevalencia de HTA en adultos de 20 años y más fue de 36.8% (23.4% ya diagnosticado y 13.4% con la medición al momento de la encuesta) (Figura 2.4).

Figura 2.4 Prevalencia de hipertensión arterial según su hallazgo por grupos de edad.

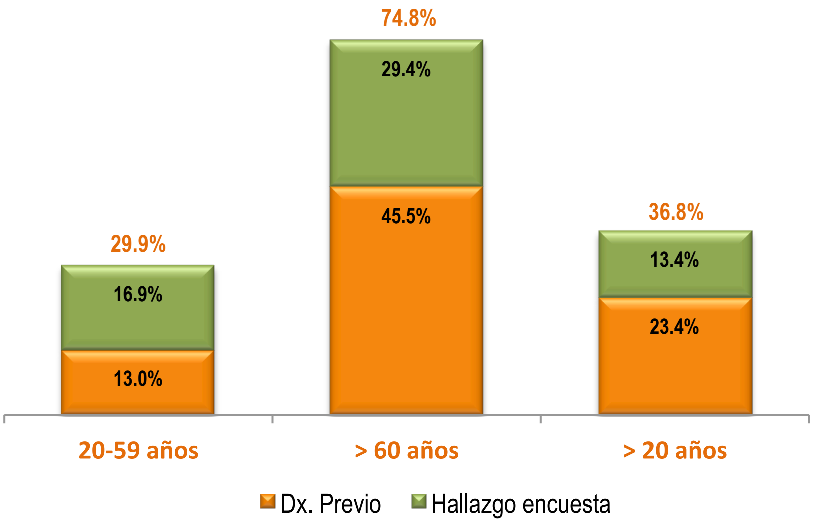

Fuente: EESN-NL 2011/2012 (Cuestionario Adultos de 20 a 59 años y 60 años y más).

Depresión

La EESN-NL 2011/2012 evaluó la presencia del diagnóstico de depresión en los grupos de edad de 20 a 59 años en 3,106 personas que representan a 950,558 personas; y en el grupo de 60 años y más a 1,471 personas que representan a 361,516 adultos mayores.

La prevalencia de depresión fue de 8.6% en los adultos de 20 a 59 años y de 15.1% en los adultos mayores. El 48.6% de los adultos de 20 a 59 años admitieron haber recibido tratamiento para la depresión, de los cuales, el 18.2% reporta haber tomado tratamiento en la semana previa a la entrevista; el 46.9% de los adultos mayores de 60 años y más admitieron haber recibido tratamiento la semana previa a la entrevista, de los cuales, el 14.2% tomó medicamento para la depresión en la semana previa a la encuesta (Tabla 2.16).

La escala de Yesavage nos da resultados de niveles de depresión, leve, moderada, severa y ausencia de la misma, en este caso la importancia de su aplicación fue identificar presencia de depresión o ausencia de la misma con o sin tratamiento.

Tabla 2.16 Diagnóstico de depresión en adultos de 20 años y más.

Presencia de depresión con o sin tratamiento	Grupo de edad	
	20 a 59 años	60 años y más
Diagnóstico de depresión		
Sí	8.6	15.1
No	91.4	84.9
Recibieron tratamiento		
Sí	48.6	46.9
No	51.4	53.1

Fuente: EESN-NL 2011/2012 (Cuestionario Adultos de 20 a 59 años y 60 años y más).

La prevalencia de depresión en adultos de 60 años y más es mayor en la Región Metropolitana con 17.6%, las regiones Norte y Centro muestran valores similares de 8.3% y 8.2% respectivamente, mientras en la Región Sur la prevalencia es de 4.4% (Tabla 2.17).

Tabla 2.17 Adultos de 60 años y más según diagnóstico de depresión, dividido por región geográfica

Diagnóstico de depresión	Región				Estatal
	Metropolitana %	Norte %	Sur %	Centro %	%
Sí	17.6	8.3	4.4	8.2	15.1
No	82.4	91.7	95.6	91.8	84.9
Total	100.0	100.0	100.0	100.00	100.00

Fuente: EESN-NL 2011/2012 (Cuestionario Adultos de 60 años y más).

Evaluación cognitiva y física

La EESN-NL 2011/2012 evaluó la presencia de deterioro cognitivo mediante la aplicación de dos cuestionarios a 1,471 adultos de 60 años y más que representan a 362,109 personas. El primero consistió en la aplicación de la Escala de Pfeiffer que consta de 10 ítems y mide el grado de función cognitiva, principalmente sustracción y la capacidad para retener y recordar información. De la población estudiada, 20.6% presenta deterioro cognitivo leve, 8.3% deterioro cognitivo moderado y 2.7% deterioro cognitivo severo (Figura 2.5).

El segundo instrumento fue el Índice de Barthel, que mide el grado de desempeño en las actividades de la vida diaria y la movilidad, siendo un marcador de la capacidad para vivir solo en casa, grado de independencia al darse de alta de un hospital, grado de cambio funcional en pacientes en rehabilitación y en la predicción del desempeño funcional después de un evento vascular cerebral. De la población encuestada, 86.7% es independiente en su vida libre, 0.8% presenta dependencia total, 3.6% dependencia severa, 4.9% dependencia moderada y 4.0% dependencia escasa. Los porcentajes de adultos de 60 años y más con deterioro cognitivo en las cuatro regiones tienen comportamiento similar (Tabla 2.18).

Figura 2.5 Prevalencia de deterioro cognitivo según la Escala de Pfeiffer en adultos de 60 años y más.

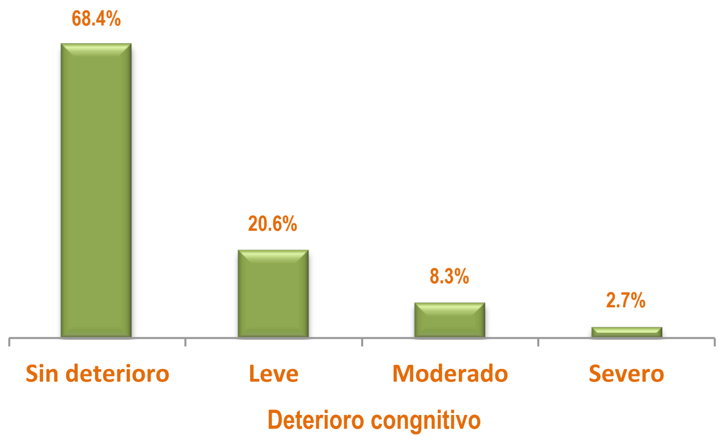

Fuente: EESN-NL 2011/2012 (Cuestionario Adultos de 60 años y más).

Tabla 2.18 Nivel de dependencia física según el Índice de Barthel en adultos de 60 años y más, dividido por región geográfica.

Dependencia física	Región				Estatal
	Metropolitana	Norte	Sur	Centro	
	%	%	%	%	%
Total	0.8	0.0	2.9	0.6	0.8
Severa	3.8	5.1	2.9	2.3	3.6
Moderada	4.2	3.1	5.7	9.4	4.9
Escasa	4.0	2.0	2.9	5.3	4.0
Independiente	87.2	89.8	85.7	82.5	86.7
Total	100.0	100.0	100.0	100.0	100.0

Fuente: EESN-NL 2011/2012 (Cuestionario Adultos de 60 años y más).

El grado de fragilidad se evaluó con el cuestionario de Barber, prueba que determina el riesgo de dependencia, reportada con utilidad para seleccionar a personas con riesgo de hospitalización, institucionalización o muerte, validada con alta sensibilidad para predecir caídas, vulnerabilidad, riesgo de enfermedad y aislamiento social. De las personas encuestadas, se describe con fragilidad el 56.5% y sin fragilidad el 43.5%. La Región Metropolitana presentó el mayor índice de fragilidad: 60.5% (Tabla 2.19).

Tabla 2.19. Prevalencia de fragilidad según cuestionario de Barber, en adultos de 60 años y más, dividido por región geográfica.

Presencia de fragilidad	Región				Estatal
	Metropolitana	Norte	Sur	Centro	
	%	%	%	%	%
Si	60.5	49.6	30.0	47.2	56.5
No	39.5	50.4	70.0	52.8	43.5
Total	100.0	100.0	100.0	100.0	100.0

Fuente: EESN-NL 2011/2012 (Cuestionario Adultos de 60 años y más).

Cáncer

La prevalencia de cáncer en el grupo de edad de 20 a 59 años fue de 0.8%, de un total de 2,768 personas. En el grupo de adultos mayores (60 años y más), se evaluaron 1,475 personas y la prevalencia fue de 2.1%. El cáncer más frecuente en mujeres fue el de cérvix 58.8%, seguido por el cáncer de mama 29.4%. En los hombres, el cáncer de próstata fue el más frecuente 14.2% (Tabla 2.20).

Tabla 2.20 Prevalencia de cáncer* en adultos de 20 años y más, según género.

Grupo de edad	Tipo de cáncer	Género Hombres %	Mujeres %	Estatal %
20-59 años	Piel (melanoma)	0.0	5.3	8.3
	Mama	0.0	26.3	41.7
	Próstata	0.0	10.5	16.7
	Otro	25.0	15.8	33.3
60 años y más	Cérvix	0.0	58.8	41.7
	Mama	0.0	29.4	21.7
	Próstata	14.2	0.0	4.0
	Leucemia	0.0	11.7	8.3
	Otro	85.7	0.0	24.0

Fuente: Fuente: EESN-NL 2011/2012 (Cuestionario Adultos de 20 a 59 años y 60 años y más).
*En la encuesta también fueron incluidos otros tipos de cáncer (estómago, pulmón y otros), que no fueron referenciados por los encuestados.

No se observa diferencia en las prevalencias de cáncer en el grupo de edad de 20 a 59 años por región. En el grupo de 60 años y más la prevalencia de cáncer fue mayor en la Región Centro (Tabla 2.21).

Tabla 2.21 Prevalencia de cáncer en adultos de 20 años y más, dividido por región geográfica.

Diagnóstico de cáncer	Región Metropolitana %	Norte %	Sur %	Centro %	Estatal %
20 a 59 años	1.6	0.6	0.3	0.9	0.8
60 años y más	1.7	2.5	0.0	4.3	2.1

Fuente: EESN-NL 2011/2012 (Cuestionario Adultos de 20 a 59 años y 60 años y más).

Salud reproductiva

La salud reproductiva es reconocida como un derecho en la mayoría de los países del mundo. Para la Organización Mundial de la Salud, la salud reproductiva es la condición de bienestar físico, mental y social vinculada al sistema reproductivo. Abarca aspectos de sexualidad y reproducción en todas las etapas de la vida. Promueve que la población goce de una vida sexual segura y satisfactoria, con capacidad de tener hijos y la libertad de decidir si quiere tenerlos, cuándo y con qué frecuencia;

supone el derecho de hombres y mujeres de estar informados y tener acceso a los servicios de planificación familiar y salud reproductiva, que permitan la asistencia profesional a la mujer embarazada y durante el parto, asegurando el nacimiento de hijos sanos.

El estudio de la salud reproductiva en la EESN-NL 2011/2012 tomó en cuenta a la población de 12 años y más, describiendo aspectos de educación y planificación familiar, antecedentes de embarazo, instituciones y personal que atendió a las embarazadas, y acciones realizadas para el control de la gestación, entre otros.

- **Métodos de anticoncepción (conocimiento y uso)**

En el grupo de 12 a 19 años se preguntó a 965 personas, que representan a 351,411 adolescentes de Nuevo León, sobre el conocimiento de métodos anticonceptivos. La encuesta reveló que 89.5% de los jóvenes sí conocía o había oído hablar de algún método para evitar el embarazo, cifra similar a la reportada por la ENSANUT 2012 (90.0%) (Tabla 2.22). En el ámbito regional de Nuevo León, el comportamiento fue similar. En relación a tener o no pareja, de 493 mujeres de 12 a 19 años que refirieron haber estado embarazadas, que representan a 162,267 adolescentes, 65.7% negaron tener actualmente una pareja; si consideramos solo aquellas que tienen pareja (34.3%), el 34.2% utiliza algún método para prevenir embarazos.

En cuanto a las mujeres de 20 a 49 años, se entrevistó a 1,746 mujeres que representan a 521, 291 mujeres de Nuevo León, de estas, 82.8% sí tienen pareja y de ellas 62.4% evita el embarazo a través del uso de métodos de anticoncepción (Tabla 2.23 y 2.24).

Tabla 2.22 Adolescentes de 12 a 19 años que conocen o han oído hablar de métodos anticonceptivos, dividido por región geográfica.

Conocimiento de métodos anticonceptivos	Región				Estatal
	Metropolitana %	Norte %	Sur %	Centro %	%
SÍ	89.7	86.0	87.5	89.5	89.5
No	10.3	14.0	12.5	10.5	10.5
Total	100.0	100.0	100.0	100.0	100.0

Fuente: EESN-NL 2011/2012 (Cuestionario Niños de 10 a 19 años).

Tabla 2.23 Mujeres con pareja que utilizan métodos anticonceptivos.

	Tiene pareja	Uso de métodos anticonceptivos
Adolescentes de 12 a 19 años	34.3%	34.2%
Adultas de 20 a 59 años	82.8%	62.4%

Fuente: EESN-NL 2011/2012 (Cuestionario Niños de 0 a 9 años y Adultos de 20 a 59 años).

Tabla 2.24 Adultos de 20 a 49 años que conocen o han oído hablar de métodos anticonceptivos, dividido por región geográfica.

Método anticonceptivo	Región				Estatal
	Metropolitana	Norte	Sur	Centro	
	%	%	%	%	%
Sí	59.6	68.5	58.1	65.1	62.4
No	40.4	31.5	41.9	34.9	37.6
Total	100.0	100.0	100.0	100.0	100.0

Fuente: EESN-NL 2011/2012 (Cuestionario Adultos de 20 a 59 años).

• Tipo de métodos anticonceptivos

En relación con el uso de métodos anticonceptivos en el Estado, los métodos más utilizados son la salpingoclasia (operación femenina o ligadura) con 44.7%, el dispositivo intrauterino (DIU) 20.0%, pastillas o píldoras anticonceptivas 9.9% y preservativo o condón 9.8% (Figura 2.9).

Figura 2.9 Métodos anticonceptivos

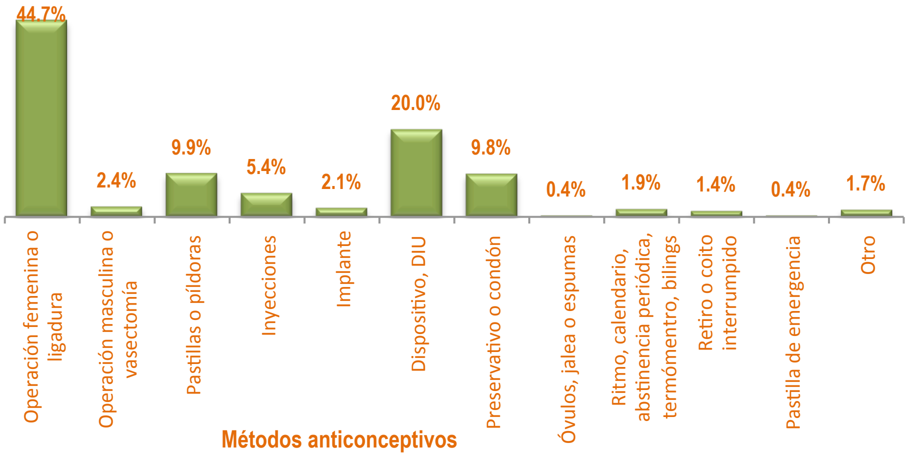

Fuente: EESN-NL 2011/2012 (Cuestionario Adultos de 20 a 59 años).

De los encuestados que refirieron utilizar algún método anticonceptivo de 20 a 49 años de edad, el tipo de método más utilizado en las diferentes regiones del Estado fue la operación femenina o ligadura, encontrándose que en la Región Norte predomina más su uso con 51.6%, seguido de la Región Centro con una prevalencia de 48.3%, en la Región Metropolitana el uso de este método fue de 45.9% y con menor uso en la Región Sur (34.3%).

Los métodos de anticoncepción que no tienen prevalencia: en la Región Metropolitana fue el empleo de implante, en la Región Norte el uso de la pastilla de emergencia y en la Región Centro tanto el uso de óvulos, jaleas o espuma y pastilla de emergencia. En la Región Sur, el tipo de método menos utilizado para evitar embarazos fue el empleo de óvulos, jaleas o espumas, encontrándose sólo 0.7% (Tabla 2.25).

Tabla 2.25 Adultos de 20 a 49 años según acciones* realizadas para no tener hijos, dividido por región geográfica.

Acciones	Región			
	Metropolitana %	Norte %	Sur %	Centro %
Operación femenina o ligadura	45.9	51.6	34.3	48.3
Operación masculina o vasectomía	3.7	2.4	1.3	2.1
Pastillas o píldoras	4.1	12.7	12.0	10.8
Inyecciones	1.5	5.6	11.0	3.2
Implante	0.0	3.2	4.7	0.4
Dispositivo, DIU	27.8	13.9	18.0	19.9
Preservativo o condón	10.4	6.0	12.7	9.8
Óvulos, jalea o espumas	0.4	0.4	0.7	0.0
Ritmo, calendario, abstinencia periódica, termómetro, Billings	3.0	2.0	1.0	1.8
Retiro o coito interrumpido	1.5	1.6	1.7	1.1
Pastilla de emergencia	0.4	0.0	1.0	0.0
Otro	1.5	0.8	1.7	2.8

Fuente: EESN-NL 2011/2012 (Cuestionario Adultos de 20 a 59 años).
*En la tabla se muestra información sobre los principales métodos utilizados por región geográfica.

• Antecedentes de Embarazo

En relación al antecedente de embarazo en las jóvenes de 12 a 19 años, el 7.0% refirió haber estado embarazada. El 31.9% de los adolescentes utilizan algún método anticonceptivos (Tabla 2.26).

El 75.0% dijeron no haber presentado complicaciones durante el parto. El 25.0% de las jóvenes de la Región Norte mencionaron haber presentado complicaciones durante el parto y 32.3% en el área Metropolitana; mientras que el 100.0% de la Región Sur dijeron no haber presentado complicaciones durante el embarazo. (Tabla 2.27).

Tabla 2.26 Mujeres adolescentes de 12 a 19 años que ellas o sus parejas están haciendo algo para no tener hijos, dividido por región geográfica.

Anticonceptivo	Región				Estatal
	Metropolitana	Norte	Sur	Centro	
	%	%	%	%	%
Sí	31.3	33.3	66.7	28.6	31.9
No	61.2	66.7	33.3	64.3	61.1
Está embarazada	7.5	0.0	0.0	7.1	7.0
Total	100.0	100.0	100.0	100.0	100.0

Fuente: EESN-NL 2011/2012 (Cuestionario Adolescentes de 10 a 19 años).

Tabla 2.27 Antecedentes de embarazo* en mujeres adolescentes de 12 a 19 años, dividido por región geográfica.

Antecedente	Embarazo	Región				Estatal
		Metropolitana	Norte	Sur	Centro	
		%	%	%	%	%
Han estado embarazadas alguna vez	Sí	6.1	13.0	16.7	9.3	6.9
	No	93.9	87.0	83.3	90.7	93.1
Según conclusión del embarazo	Nacidos vivos aunque hayan muerto	100.0	100.0	100.0	100.0	100.0
Con complicación al momento del parto[(1)]	Sí	32.3	25.0	0.0	0.0	25.0
	No	67.7	75.0	100.0	100.0	75.0

Fuente: EESN-NL 2011/2012 (Cuestionario Adolescentes de 10 a 19 años).
*En la encuesta también fueron incluidos otros antecedentes de embarazo, como nacidos muertos y abortos, que no fueron referidos por los encuestados.
[(1)] En las regiones Sur y Centro no fueron referenciadas complicaciones al momento del parto.

Las mujeres mayores de 20 años brindaron información relacionada a los antecedentes de embarazo y niños nacidos con un peso mayor a los 4kg. (niños macrosómicos), destacando que en el grupo de 20 a 59 años 88.3% tuvieron antecedentes de embarazo, de estas 19.1% mencionaron haber tenido hijos de peso mayor a los 4 kg al momento del nacimiento, mientras que en el grupo de 60 años y más 91.9% tuvieron antecedentes de embarazo, de estas 31.2% mencionaron haber tenido hijos nacidos con un peso mayor a los 4kg.

A nivel regional, en el grupo de mujeres de 20 a 59 años el antecedente de hijo con peso menor a 4kg. se presentó en un rango de 12.2% a 22.5%, mientras que en el grupo de 60 años y más el antecedente hijo con peso menor a 4kg. se presentó en un rango de 23.1% a 34.4% (Tabla 2.28).

Tabla 2.28 Mujeres de 20 años y más, según antecedentes de hijos con peso mayor a 4 kg. al nacimiento, dividido por región geográfica.

Grupo de edad	Antecedente de hijo con peso mayor a 4 kg. al nacimiento	Región				Estatal
		Metropolitana %	Norte %	Sur %	Centro %	%
20 a 59 años	Si	21.3	22.5	12.2	20	19.1
	No	78.7	77.5	87.8	80	80.9
60 años y más	Sí	30.7	32.7	23.1	34.4	31.2
	No	69.3	67.3	76.9	65.6	68.8

Fuente: EESN-NL 2011/2012 (Cuestionario Adultos de 20 a 59 años y 60 años y más).

- ## Atención del embarazo, instituciones y personal de salud

Se interrogó a 63 madres de niños menores de un año que representan a 14,073 mujeres sobre las instituciones de salud que atendieron sus embarazos; 55.6% fueron atendidas por el Instituto Mexicano del Seguro Social (IMSS), 22.2% por la Secretaría de Salud/Seguro Popular, 11.1% por servicios particulares y el resto por otras instituciones (Tabla 2.29).

Tabla 2.29 Madres de niños menores de 1 año según institución de salud donde fue atendido su embarazo, dividido por región geográfica.

Institución	Región				Estatal
	Metropolitana	Norte	Sur	Centro	
	%	%	%	%	%
IMSS	67.3	0.0	0.0	33.3	55.6
SSA Centro de salud u hospital	0.0	40.0	100.0	16.7	9.5
Programa Seguro Popular(1)	10.2	20.0	0.0	33.3	12.7
PEMEX	0.0	0.0	0.0	16.7	1.6
Consultorio u hospital privado	14.3	0.0	0.0	0.0	11.1
Otra(2)	8.2	40.0	0.0	0.0	9.5

Fuente: EESN-NL 2011/2012 (Cuestionario Adultos de 20 a 59 años).

(1) Se refiere al porcentaje de la población que financió la atención médica durante el embarazo a través del programa del Seguro Popular, sin especificar la institución en la que fue atendido.

(2) En la encuesta también fueron incluidas otras instituciones de salud como IMSS Oportunidades, ISSSTELEON, ISSSTE, Marina/Defensa, Cruz Roja y otras que no fueron referidas por los encuestados.

En relación a la atención del último parto se interrogó a 2,027 mujeres de 20 a 59 años que representan a 617,613, destacando que 50.8% fueron atendidas en el IMSS a nivel estatal (Tabla 2.30 y Figura 2.10).

Tabla 2.30 Lugar o institución donde se atendió el último parto de mujeres de 20 años y más, dividido por región geográfica.

Institución	20 a 59 años Región				Estatal	60 años y más Región				Estatal
	Metropolitana	Norte	Sur	Centro		Metropolitana	Norte	Sur	Centro	
	%	%	%	%	%	%	%	%	%	%
IMSS Oportunidades	0.3	0.0	0.0	0.3	0.3	0.0	0.0	0.0	2.9	0.4
IMSS	53.2	29.4	9.8	50.1	50.5	45.3	24.6	5.9	35.9	41.6
Secretaría de Salud	10.2	37.6	72.5	13.4	13.4	2.8	15.8	35.3	9.7	5.4
Seguro Popular	2.6	5.7	5.7	3.2	2.9	0.0	0.0	0.0	0.0	0.0
DIF, Cruz Roja, INI	0.0	0.9	0.0	0.0	0.1	1.2	0.0	0.0	0.0	0.9
ISSSTE, ISSSTE estatal	1.8	1.8	0.9	1.5	1.7	4.3	1.8	0.0	1.9	3.7
Marina/Defensa/Pemex	0.4	0.2	0.0	8.1	1.3	0.7	0.0	0.0	0.0	0.5
Particular	23.5	14.6	2.1	15.6	21.6	15.3	17.5	5.9	21.4	16.1
Hospital Civil	2.8	1.8	1.0	2.3	2.6	12.5	5.3	0.0	2.9	10.4
Casa de la entrevistada	0.6	2.6	2.4	0.3	0.7	7.3	24.6	41.2	11.7	9.9
Otro lugar*	1.8	1.7	0.2	0.4	1.6	1.5	3.5	0.0	1.9	1.7
Casa de la partera	0.2	0.0	4.8	0.6	0.3	3.3	7.0	11.8	10.7	4.8
Otras instituciones(1)	2.8	3.8	0.5	4.2	3.0	5.8	0.0	0.0	1.0	4.6

Fuente: EESN-NL 2011/2012 (Cuestionario Adultos de 20 a 59 años y 60 años y más).

(1) En la encuesta también se incluyeron “otro lugar” y “otras instituciones”, que no fueron referidas por los encuestados.

Figura 2.10 Lugar de atención del último parto por grupo de edad dividido por región geográfica.

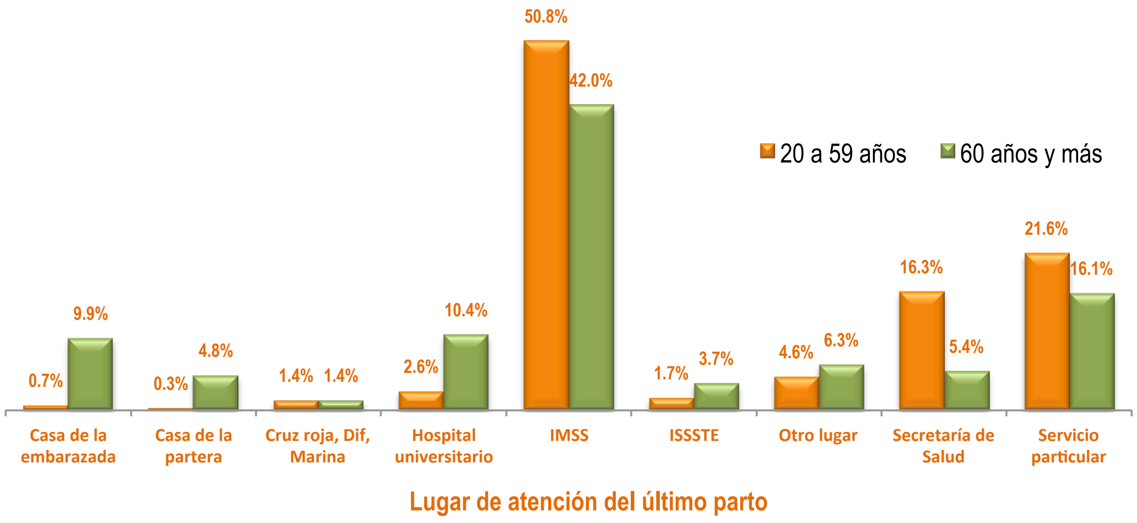

Fuente: EESN-NL 2011/2012 (Cuestionario Adultos de 20 a 59 años y 60 años y más).

En cuanto al personal de salud que atendió el último parto de las mujeres de 20 a 59 años, los resultados refieren que el 96.2% fueron atendidas por médicos (ENSANUT 2012 reportó 95.0%). A nivel regional para este grupo de edad el rango fue de 92.4% a 99.5%, mientras que en el grupo de edad de 60 años y más el 84.9% fueron atendidos por médicos en su último parto con un rango de 47.1% a 88.5% a nivel regional (Tabla 2.31).

Tabla 2.31 Mujeres de 20 años y más, según personal de salud* que atendió su último parto, dividido por región geográfica.

Grupo de edad	Personal de salud	Región				Estatal
		Metropolitana	Norte	Sur	Centro	
		%	%	%	%	%
20 a 59 años	Médico	99.5	96.4	92.4	96.6	96.2
	Enfermera	0.0	2.0	0.5	2.1	1.1
	Partera	0.5	1.4	7.0	1.0	2.5
	Nadie	0.0	0.3	0.0	0.3	0.1
	Total	100.0	100.0	100.0	100.0	100.0
60 años y más	Médico	88.5	72.4	47.1	76.9	84.9
	Enfermera	0.7	5.2	0.0	1.0	1.0
	Partera	9.5	22.4	52.9	22.1	13.1
	Nadie	1.3	0.0	0.0	0.0	1.0
	Total	100.0	100.0	100.0	100.0	100.0

Fuente: EESN-NL 2011/2012 (Cuestionario Adultos de 20 a 59 años y 60 años y más).
* En la encuesta también fueron incluidos tipos de personal de salud como promotora, auxiliar o asistente de salud, que no fueron referenciados a las encuestadas.

Daños a la salud por accidente

Los accidentes representan un riesgo para la salud y supervivencia de la población; como problema de salud pública, pueden generar consecuencias en la mortalidad, morbilidad y discapacidad. El análisis de daños a la salud causado por accidentes en los doce meses previos a la encuesta fue considerado en toda la población, identificando una prevalencia promedio de 5.7%. El rango de prevalencias por grupo de edad es de 5.9% al 8.7% (Figura 2.13).

Figura 2.13 Prevalencia de daños a la salud por accidente según grupos de edad.

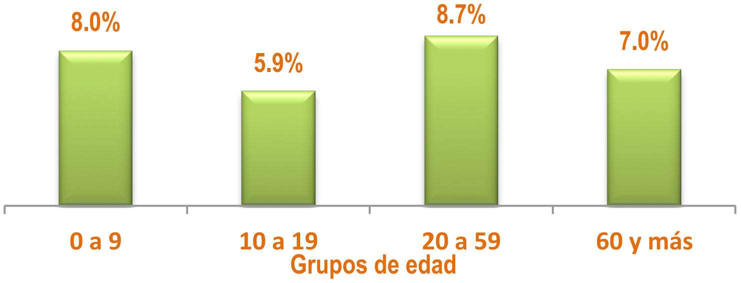

Fuente: EESN-NL 2011/2012 (Cuestionario Niños de 0 a 9 años, Adolescentes de 10 a 19 años, Adultos de 20 a 59 años y Adultos de 60 años y más).

Los daños a la salud ocasionados por accidentes en los niños divididos por región geográfica se muestran en la Tabla 2.33, donde las caídas de un nivel a otro y en un mismo nivel son los más frecuentes en este grupo de edad.

Tabla 2.33 Niños de 0 a 9 años según tipo de accidente que ocasionó daño a la salud en los últimos doce meses previos a la encuesta, dividido por región geográfica.

Tipo de accidente	Región				Estatal
	Metropolitana %	Norte %	Sur %	Centro %	%
Atropellamiento	5.7	0.0	0.0	0.0	4.6
Caída de un nivel a otro (1)	25.0	0.0	100.0	15.8	23.9
Caída de un mismo nivel	50.0	100.0	0.0	52.6	50.5
Exposición a fuerzas mecánicas inanimadas(2)	4.5	0.0	0.0	21.1	7.3
Exposición a fuego, humo y llamas (3)	1.1	0.0	0.0	5.3	1.8
Contacto con calor y sustancias calientes	10.2	0.0	0.0	5.3	9.2
Exposición accidental a otros factores (4)	3.4	0.0	0.0	0.0	2.8

Fuente: EESN-NL 2011/2012 (Cuestionario Niños de 0 a 9 años).

(1)Incluye: caída desde un mueble, escalera, andamio, árbol, edificio o casa habitación.
(2)Incluye: golpes, aplastamiento por o contra objetos, maquinaria o equipos, herramientas, vidrios, explosiones accidentales, cuerpos extraños en ojo u orificio natural.
(3) Incluye: Incendios, fogatas, chimeneas, ignición de combustibles, ropa, etc.
(4) Incluye: exceso de esfuerzo físico, privación de alimentos y agua y exposición a otros factores.

Los daños a la salud ocasionados por accidentes en los adolescentes, así como los lugares más frecuentes en los que sucedieron (caída de un mismo nivel, escuela y vía pública), divididos por región geográfica se muestran en las Tablas 2.34 y 2.35.

Tabla 2.34 Adolescentes de 10 a 19 años según tipo de accidente que ocasionó daño a la salud en los últimos doce meses, dividido por región geográfica.

Tipo de accidente	Región				Estatal
	Metropolitana %	Norte %	Sur %	Centro %	%
Choque de o entre vehículos	0.0	0.0	0.0	10.0	1.4
Atropellamiento	3.3	0.0	0.0	10.0	4.1
Caída de un nivel a otro[1]	5.0	0.0	0.0	30.0	8.2
Caída de un mismo nivel	60.0	50.0	100.0	30.0	56.2
Exposición a fuerzas mecánicas inanimadas[2]	6.7	0.0	0.0	0.0	5.5
Exposición a fuerzas mecánicas animadas[3]	0.0	0.0	0.0	10.0	1.4
Exposición a fuego y/o humo[4]	5.0	0.0	0.0	0.0	4.1
Contacto con calor y sustancias calientes	0.0	0.0	0.0	10.0	1.4
Otros accidentes que obstruyen la respiración[5]	5.0	0.0	0.0	0.0	4.1
Exposición accidental a otros factores	15.0	50.0	0.0	0.0	13.7

Fuente: EESN-NL 2011/2012 (Cuestionario Adolescentes de 10 a 19 años).
[1]Incluye: caída desde un mueble, escalera, andamio, árbol, edificio o casa habitación.
[2]Incluye: golpes, aplastamiento por o contra objetos, maquinaria o equipos, herramientas, vidrios, explosiones accidentales, cuerpos extraños en ojo u orificio natural.
[3]Incluye: Mordedura, golpes, patada o picadura de animal no ponzoñoso como rata, perro, cocodrilo, etc. Aplastamiento por estampida humana y contacto con plantas no venenosas.
[4]Incluye: Incendios, fogatas, chimeneas, ignición de combustibles, ropa, etc.
[5]Incluye: sofocación o estrangulamiento accidental, inhalación de contenido gástrico, alimentos u objetos, encierro en lugares herméticamente cerrados, sofocación por bolsa de plástico.

Tabla 2.35 Adolescentes de 10 a 19 años según el lugar donde ocurrió el accidente, dividido por región geográfica.

Lugar	Región				Estatal
	Metropolitana %	Norte %	Sur %	Centro %	%
Hogar	13.4	0.0	0.0	20.0	13.8
Escuela	47.8	0.0	0.0	10.0	41.3
Trabajo	9.0	0.0	0.0	10.0	8.8
Vía pública	9.0	50.0	100.0	30.0	13.8
Campo	3.0	0.0	0.0	0.0	2.5
Lugar de recreo o deportivo	7.5	0.0	0.0	10.0	7.5
Establecimiento comercial	0.0	50.0	0.0	0.0	1.3
Otro	0.0	0.0	0.0	10.0	1.3

Fuente: EESN-NL 2011/2012 (Cuestionario Adolescentes de 10 a 19 años).

Factores de riesgo

En esta sección se describen los hallazgos encontrados en relación a dos factores de riesgo asociados a enfermedades crónicas degenerativas: alcohol y tabaco.

• Tabaquismo

En la EESN-NL 2011/2012 se preguntó a 5,919 individuos que representan a 1,783,336 personas de 10 años y más sobre el consumo de cigarrillos de tabaco. El 66.3% mencionó haber fumado en algún momento de su vida y 21.1% declaró haber consumido más de 100 cigarrillos a lo largo de su vida. En la tabla siguiente se desglosa la información por grupos de edad y región geográfica.

Tabla 2.36 Adultos de 20 a 59 años según tabaquismo activo, dividido por región geográfica.

Tabaquismo activo	Región				Estatal
	Metropolitana %	Norte %	Sur %	Centro %	%
Sí	22.5	16.2	13.3	18.0	17.4
No	77.5	83.8	86.7	82.0	82.6
Total	100.0	100.0	100.0	100.0	100.0

Fuente: EESN-NL 2011/2012 (Cuestionario Adultos de 20 a 59 años).

Tabla 2.37 Consumo de por lo menos cien cigarrillos durante su vida según grupo de edad, dividido por región geográfica.

Grupo de edad	Consumo de Tabaco	Región				Estatal
		Metropolitana %	Norte %	Sur %	Centro %	%
10 a 19 años	Sí	7.2	3.4	4.8	5.9	6.8
	No	54.4	62.1	61.9	45.9	54.1
	Nunca ha fumado	38.4	34.5	33.3	48.2	39.1
	Total	100.0	100.0	100.0	100.0	100.0
20 a 59 años	Sí	27.9	18.7	15.6	21.1	26.3
	No	41.7	51.2	53.1	47.9	43.2
	Nunca ha fumado	30.4	30.1	31.3	31	30.5
	Total	100.0	100.0	100.0	100.0	100.0
60 años y más	Sí	22.4	25.0	23.3	24.6	23.0
	No	40.4	48.3	53.5	40.9	41.5
	Nunca ha fumado	37.2	26.7	23.3	34.5	35.5
	Total	100.0	100.0	100.0	100.0	100.0

Fuente: EESN-NL 2011/2012 (Cuestionario Adultos de 20 a 59 años).

- ## Alcohol

El consumo de alcohol fue investigado en 4,494 personas de 20 años y más, de estos, 91.0% reportó haberlo consumirlo en alguna ocasión, de los cuales 22.3% lo consume actualmente, 9.0% de individuos reportó que nunca ha tomado alcohol. Los resultados por grupos de edad y género se muestran en la Figura 2.14 destacando en todos los grupos de edad un mayor consumo en los hombres; el grupo de los adultos mayores presentó el menor porcentaje.

Figura 2.14 Consumo regular de alcohol en la población por grupos de edad y género

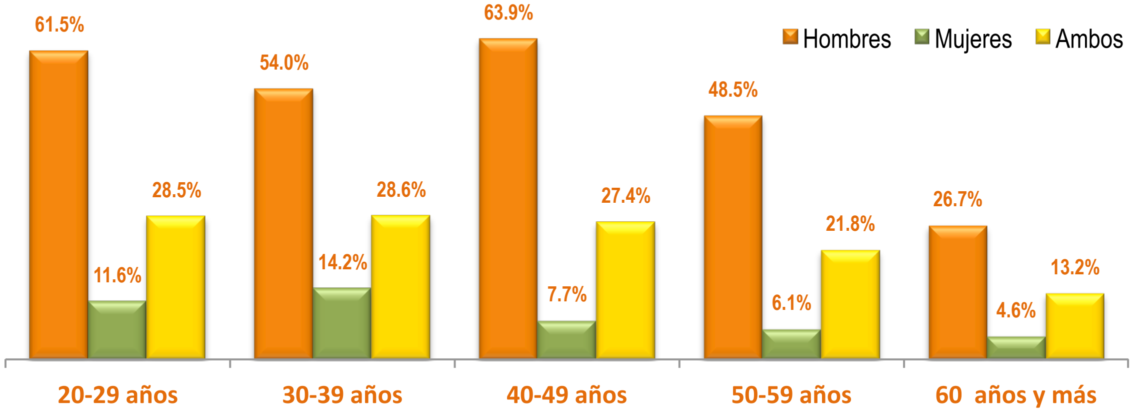

Fuente: EESN-NL 2011/2012 (Cuestionario Adultos de 20 a 59 años y 60 años y más).

Los rangos de la ingesta de alcohol en las regiones del Estado para el grupo de 20 a 59 años de edad va de 17.3% a 27.4%, mientras que para el grupo de 60 años y más el rango fue de 8.3% a 14.3%. (Tabla 2.38).

Tabla 2.38. Ingesta de alcohol en adultos por grupos de edad, dividido por región geográfica.

Grupo de edad	Ingesta de alcohol	Región				Estatal
		Metropolitana	Norte	Sur	Centro	
		%	%	%	%	%
20 a 59 años	Sí	27.4	17.3	19.5	22.2	21.6
	No	65.4	69.1	62.0	61.7	64.4
	Nunca ha tomado	7.3	13.6	18.5	16.1	13.9
60 años y más	Sí	14.3	8.3	15.6	8.8	13.1
	No	74.6	75.0	68.9	67.8	73.5
	Nunca ha tomado	11.0	16.7	15.6	23.4	13.4

Fuente: EESN-NL 2011/2012 (Cuestionario Adultos de 20 a 59 años y 60 años y más).

Resultados relevantes

- La prevalencia de Enfermedades Diarreicas Agudas (EDA) en menores de cinco años en Nuevo León es de 5.1%, menor a la reportada en la ENSANUT 2012 (11.6%).
- La prevalencia de Infecciones Respiratorias Agudas presentes en las dos semana previas a la encuesta fue de 37.3% en menores de 10 años, prevalencia inferior a la reportada nacionalmente (42.1%).
- La falta de actividad física sobre todo en los menores de 20 años de edad, es motivo de preocupación, ya que 70 % de los niños y adolescentes no realizan ninguna actividad física vigorosa y solo el 32% realiza actividad física leve durante toda la semana.
- En los adultos mayores de 20 años 82.6 % no realizan ninguna actividad física vigorosa y solo 28.13% realizan actividad física ligera durante toda la semana.
- La prevalencia de diabetes tipo 2 en adultos mayores de 20 años fue de 14.6%, dato superior al reportado a nivel nacional por la ENSANUT 2012 (9.2%) para el mismo grupo de edad.

- En adultos de 60 años y más la prevalencia de diabetes tipo 2 es de 27.4%, porcentaje mayor a lo que ENSANUT 2012 reportó nacionalmente, en los diferentes grupos de edad (60 a 69, 70 a 79 y 80 y más años), un rango de 19.9% a 25.3%.

- La prevalencia de hipertensión arterial en adultos de 20 a 59 años fue de 13.0%. En el grupo de 60 años la prevalencia fue de 45.5%, dato superior al nacional, reportado por la ENSANUT 2012 por grupo de edad (60 a 69, 70 a 79 y 80 y más años), con un rango de 37.5% a 40.2%.

- La prevalencia de hipertensión arterial al momento de la encuesta fue de 16.9% en adultos de 20 a 50 años, lo que incrementa la prevalencia estatal a 29.9% en ese grupo de edad.

- En adultos de 60 años y más, la prevalencia de hipertensión arterial al momento de la encuesta fue de 13.4%, lo que incrementa la prevalencia estatal a 36.8% en este grupo.

- En el diagnóstico de depresión en los adultos de 60 años y más la prevalencia fue de 15.1%, mientras que en el grupo de 20 a 59 años la prevalencia de depresión fue de 8.6%, casi la mitad de ellos admiten haber recibido tratamiento (46.9% y 48.6% respectivamente).

- En los adultos mayores de 60 años de edad la presencia de deterioro cognitivo moderado a severo fue de un 11.0%, que aunado al 20.6% de deterioro leve representan a una tercera parte de la población estudiada que requerirán ayuda para su cuidado.

- La presencia de fragilidad en este grupo de edad fue del 56.5%, situación de alerta por sufrir caídas y fracturas, que requieren la posibilidad de hospitalizaciones.

- La cobertura de esquemas completos de vacunación en el Estado fue de 64.8% en niños de 4 años, 58.4% en los de 3 años, 41.7% en los de 2 años, 15.3% en los de 1 año y 31.0 % en los menores de 1 año de edad, cifras inferiores a la media nacional reportada en la ENSANUT 2012 para estos grupos de edad.

- La mayoría de los adolescentes, 9 de cada 10, cuentan con información sobre métodos anticonceptivos.

- Una tercera parte de las jóvenes de 10 a 19 años, tienen vida sexual activa.

- Una tercera parte de las jóvenes de 10 a 19 años con vida sexual activa usan algún método para prevenir el embarazo, en contraste con el 62.4% en el grupo de 20 a 49 años.

- 2.4% de las jóvenes de 12 a 19 años estaban embarazadas al momento de la encuesta, resultado inferior al nacional reportado en la ENSANUT 2012 (10.7%) para este grupo de edad. El 6.9% de las jóvenes tenían antecedentes de embarazo.

- Más de la mitad de los embarazos de las madres de niños menores de 1 año y de los partos de las mujeres de 20 a 59 años fueron atendidos en el IMSS.

- El 80.2% de las mujeres proporcionaron alimentación al seno materno, porcentaje menor al reportado nacionalmente por la ENSANUT 2012 (93.5%).

- La prevalencia de daño a la salud por accidentes en la población en general es de 5.7%, siendo el grupo de 0-9 años el que tiene la prevalencia más alta, 7.9%. La mayoría de los accidentes son ocasionados por caídas.

- Al menos una cuarta parte de la población de 20 a 59 años (26.3%) ha fumado más de 100 cigarrillos a lo largo de su vida, clasificándolo como fumador. 17.4% mantiene un tabaquismo activo.

- Dos de cada 10 sujetos de 20 años y más consumen alcohol actualmente. Esta cifra en los hombres de 20 a 49 años es mayor al 50.0%. En las mujeres la prevalencia no supera el 15.0%. A nivel nacional la ENSANUT 2012 reportó, para el grupo de edad de 20 años o más, en las mujeres un 67.8% y un 41.3% para los hombres.

- La prevalencia de enfermedades diarreicas es mayor en la Región Norte y Sur con 10.0% y 10.5% respectivamente, y de un 40.0% a un 85.7% se le administró Suero Vida Oral.

- La Región Metropolitana muestra el mayor porcentaje de prevalencia de infecciones respiratorias agudas con 41.1%.

- La prevalencia mayor de diabetes tipo 2 en adultos de 20 a 59 años, se observa en las regiones Centro y Metropolitana, con 12.3% y 8.5% respectivamente.

- En adultos de 60 años y más la prevalencia de diabetes fue mayor en las regiones Metropolitana y Norte con 29.4% y 24.2% respectivamente.

- En adultos de 60 años y más se observa la mayor prevalencia de hipertensión arterial, principalmente en las regiones Metropolitana y Norte con 46.6% y 45.8% respectivamente. La mayor prevalencia de depresión en adultos de 60 años y más se observa en la Región Metropolitana, con 17.6%.

- La Región Metropolitana muestra el mayor índice de fragilidad con 60.5% en adultos de 60 años y más.

- En las regiones Sur y Metropolitana más del 70.0% de los menores de cinco años muestran esquemas completos de vacunación.

- La práctica de lactancia materna es mayor en las regiones Norte y Centro del Estado con 87.5% y 84.6% respectivamente.

Capítulo 3

Estado Nutricio

3. Estado Nutricio

La valoración del estado nutricional de una población permite proponer políticas de salud de alta calidad al identificar a los individuos que se encuentran en riesgo de salud y nutrición. El estado de nutrición del individuo es complejo, por lo que una valoración integral debe incluir el análisis de la ingesta de alimentos, las mediciones antropométricas y la evaluación de indicadores bioquímicos, entre otros.

De acuerdo con la Organización de las Naciones Unidas para la Agricultura y la Alimentación (FAO), un número creciente de países emergentes presentan la doble carga de la desnutrición y la mala nutrición por exceso. Es decir, la persistencia de desnutrición, especialmente entre niños, junto con un rápido incremento de sobrepeso, obesidad y enfermedades crónicas relacionadas [Kennedy, 2006]. La desnutrición materna debida a una inadecuada alimentación durante la atención prenatal aumenta las tasas de mortalidad infantil y la posibilidad de que los niños nazcan con bajo peso [Bhan, 2003]. De igual forma, durante los dos primeros años de edad pueden presentarse deficiencias en el aporte dietario de vitaminas y minerales junto con enfermedades parasitarias y causar alteraciones en el crecimiento, como una baja talla o estatura. Por ello, los primeros años de edad representan una oportunidad para la promoción de programas de salud y nutrición en países emergentes [Alison, 2007; Martorell, 1994]. En menores de cinco años, las consecuencias asociadas a la desnutrición pueden alterar la capacidad para el trabajo físico, el desempeño intelectual y el escolar en la adolescencia y la edad adulta [Victoria, 2008].

En el otro extremo, nuestra población ha iniciado el proceso de envejecimiento demográfico. Por ello, los adultos mayores comienzan a recibir más atención a sus problemas de salud y nutrición. El proceso de envejecimiento del adulto mayor implica un aumento del riesgo de padecer osteoporosis y sarcopenia (disminución de la masa muscular). Además, en este grupo de edad se presenta naturalmente una redistribución y aumento en la proporción de grasa corporal [Visser, 2009]. Todos estos cambios pueden tener un impacto negativo para la salud y calidad de vida del adulto mayor.

Por otro lado, la mala nutrición por exceso representa un desafío emergente de salud pública. El sobrepeso y la obesidad resultan de un desequilibrio entre la ingesta y el gasto de energía. Este desequilibrio está principalmente asociado con una dieta de alta densidad energética, acoplada con una reducción en la actividad física [Astrid, 2006]. Los cambios en los hábitos de la actividad física están relacionados con la transformación de las nuevas tecnologías en las ciudades modernas y los nuevos patrones de trabajo, transporte y recreación [Jain, 2005]. El sobrepeso y la obesidad son factores de riesgo de varias enfermedades crónicas como diabetes e hipertensión arterial. Los altos costos que genera la atención de estas enfermedades pueden comprometer la estructura económica de los gobiernos involucrados [Secretaría de Salud, 2010].

En el ámbito estatal, Nuevo León cuenta con información sobre el estado nutricio de la población a través del Diagnóstico Nutriológico de las Familias (DNF) y Menores de 5 años, que se realizó en el año 2000 [FASPYN & SSA, 2000]. De acuerdo con esta encuesta, los niños preescolares presentaron 27.3% de bajo peso para la edad, mientras que los escolares 23.8%. Se observó además que 20.3% de los preescolares presentaban emaciación (bajo peso para la talla); mientras que los escolares con esta condición fueron 17.1%. En el indicador baja talla para la edad, 17.0% de los preescolares presentaron desnutrición crónica; mientras que los escolares sólo 3.2%. En cuanto a la prevalencia de sobrepeso con el indicador z >2 del peso para la talla, los preescolares clasificados con esta condición fueron 16.6%; entretanto, los escolares fueron 19.8%. Por su parte, la población adulta había presentado 54.0% de obesidad. En el 2006, la Secretaría de Salud Federal publicó los resultados de la ENSANUT (Encuesta Nacional de Salud y Nutrición del 2007) y de las entidades federativas, en este caso los correspondientes a Nuevo León [INSP & SSA, 2007]. En preescolares, el 1.7% presentó bajo peso para la edad, 4.6% baja talla y 7.4% obesidad. En escolares, 2.7% presentó baja talla y 28.6 sobrepeso y obesidad. En adultos, se reportó una prevalencia de obesidad de 71.0%.

El objetivo de este capítulo es mostrar los resultados de la evaluación nutricional de los niños, adolescentes, adultos y adultos mayores de la Encuesta Estatal de Salud y Nutrición del Estado de Nuevo León 2011/2012 (EESN-NL 2011/2012).

A continuación se describe la metodología empleada para la evaluación antropométrica y los índices e indicadores antropométricos utilizados para la evaluación nutricional de preescolares, escolares, adolescentes, adultos y adultos mayores.

Metodología

Las mediciones antropométricas fueron realizadas por personal capacitado y de acuerdo a las recomendaciones de la Organización Mundial de la Salud (OMS) [de Onis, 2004]. Todas las mediciones se realizaron del lado derecho del cuerpo y por duplicado. El promedio de dos evaluaciones fue considerado como el valor final para el análisis. El peso corporal en todos los grupos de edad se realizó utilizando una báscula electrónica (Seca, Seca 813, Hamburgo, Alemania). En niños menores de dos años el peso se midió estando en brazos de la mamá, después, se restó el peso de la madre. Cuando el peso de la mamá fue mayor a 100 kg, otra persona de menor peso sostuvo a los niños en brazos. La estatura se midió con un estadiómetro de manufactura local, con precisión en milímetros y calibrado. En menores de dos años la longitud se midió con un infantómetro (Seca 210).

En el grupo de adultos se midió además la circunferencia de cintura al nivel de la cicatriz umbilical. Las circunferencias corporales se midieron utilizando una cinta métrica de fibra de vidrio Gulick®. En los sujetos en los que no fue posible medir la estatura de pie, la talla se estimó a partir de la altura de rodilla de acuerdo con la técnica recomendada para este grupo de edad [Chumlea, 1998], utilizando un antropómetro Lafayette (LA-01290, Lafayette, EU).

• Menores de 5 años, escolares y adolescentes

Para la evaluación del estado nutricional en menores de 5 años, escolares y adolescentes de la EESN-NL 2011/2012, se empleó el patrón de referencia de la OMS 2006-2007. La OMS recomendó un nuevo patrón de referencia para niños de 0 y menores de 5 años. Posteriormente, en el 2007 se publicaron las tablas complementarias de crecimiento para niños y jóvenes de 5 a 19 [WHO, 2006; de Onis, 2007]. Los índices e indicadores sugeridos por la OMS para evaluar el crecimiento en menores de 5 años, escolares y adolescentes se muestran en la Tabla 3.1.

Es importante señalar que los puntos de corte y clasificación de sobrepeso y obesidad son distintos para menores de 5 años y niños mayores [WHO, 2006]. Será conveniente que las encuestas estatales en el país reporten en adelante el estado nutricional de niños de 0 a 19 años utilizando esta misma referencia.

Tabla 3.1 Indicadores para evaluar el estado nutricio en preescolares, escolares y adolescentes sugeridos por la OMS, 2006 – 2007.

Talla-Longitud/ edad	Peso / talla	Peso / edad	IMC/ edad preescolares	IMC/ edad escolares y adolescentes	Punto de corte puntuación Z
Muy alta	Obesidad	Sobrepeso<br>Confirmar con IMC/edad el grado de	Obesidad	Obesidad severa	> 3.0
Adecuada	Sobrepeso	Sobrepeso<br>Confirmar con IMC/edad el grado de	Sobrepeso	Obesidad	> 2.0
Adecuada	Adecuado<br>con Posible riesgo de sobrepeso	Adecuado<br>Confirmar clasificación de adecuado o grado de sobrepeso con IMC/edad	Adecuado<br>con Posible Riesgo de sobrepeso	Sobrepeso	> 1.0
Adecuada	Adecuado	Adecuado	Adecuado	Adecuado	Mediana
Adecuada	Adecuado	Adecuado	Adecuado	Adecuado	< −1.0
Desmedro	Emaciación	Bajo peso	Emaciación	Delgadez	< −2.0
Desmedro severo	Emaciación severa	Bajo peso severo	Emaciación severa	Delgadez severa	< −3.0

WHO. (2006). WHO Child Growth Standards: Methods and development: Length/height-for-age, weight-for-age, weight-for-length, weight-for-height and body mass index-for-age. Geneva: World Health Organization.

La Longitud / talla para la edad indica desnutrición crónica o el estado nutricio pasado: refleja el crecimiento lineal del esqueleto. Permite identificar a los niños con retardo en el crecimiento (desmedro o baja talla) debido a una prolongada desnutrición por falta de alimentos, por la presencia de enfermedades infeccionas frecuentes o una combinación de ambas. Del otro lado, una adecuada talla para la edad sugiere que tanto en un individuo como en una comunidad o país no se han presentado problemas de nutrición muy prolongados. El peso para la edad indica desnutrición aguda y crónica o el estado nutricio presente y pasado. Se utiliza para evaluar si un niño tiene un bajo peso o un peso bajo grave. El peso para la edad puede ser práctico para dar una idea general de los problemas de salud de una comunidad o el cambio en alguna dirección. El peso para la edad permite seguir el patrón de crecimiento de un niño en el tiempo y detectar problemas de crecimiento

antes de que sean severos. No obstante, no se sugiere utilizarlo para clasificar a un niño con sobrepeso u obesidad (15). El peso para la edad es un índice compuesto que refleja el crecimiento de un niño en términos de masa (tejido adiposo y muscular) y del esqueleto (crecimiento lineal). Por lo tanto, no puede distinguir entre niños que son pequeños para su edad pero con sobrepeso (e incluso bien proporcionados), de aquellos niños quienes son altos y delgados, lo que es importante para clasificar el estado de nutrición. El peso para la estatura (peso para la talla) indica desnutrición aguda o el estado nutricio presente. Evalúa la ganancia o pérdida tanto de tejido adiposo como muscular. Es un indicador que solventa los problemas con el uso del peso para la edad pues permite distinguir entre niños que son pequeños para su edad (desmedro) y con bajo peso (en el presente), de aquellos quienes son pequeños pero no desnutridos (sin bajo peso) en el presente. El término emaciación indica un desgaste del tejido adiposo y muscular. El bajo peso para la talla es un indicador especialmente útil en situaciones donde no es posible conocer la edad del niño (por ejemplo, en refugiados). El peso para la talla es un índice que puede ser suficiente para realizar un tamizaje del estado nutricio en situaciones de emergencia. Al igual que el índice de masa corporal, el peso para la talla puede reflejar la incapacidad para ganar o perder peso. Las tablas de peso para la longitud y peso para la estatura ayudan a identificar a los niños con bajo peso para la talla, es decir, que pueden estar emaciados o emaciados gravemente. El índice también permite identificar a los niños con peso para la estatura elevado y que podrían estar en riesgo de tener sobrepeso u obesidad. El IMC esperado para la edad es un índice que es especialmente útil para detectar el riesgo de sobrepeso y obesidad. El IMC indica si el peso corporal es excesivo o insuficiente en relación con la estatura. En este sentido, es más útil que el peso para la talla y por ello las nuevas tablas de la OMS consideran la evaluación del riesgo de sobrepeso con el IMC/edad desde el primer año de vida hasta los 19 años.

Para el análisis del índice estatura para la edad se consideraron como valores válidos los que se encontraron entre -5.5 y +3.0 puntaciones z. Para el índice peso para la edad fueron válidos los valores entre -5.0 y +5.0 unidades z. Para el peso para la talla aquellos entre -4.2 y +5.0 unidades z, respecto a la media de las tablas de referencia. Se excluyó del análisis la información de los individuos con datos incompletos en peso o talla. En el caso del grupo de preescolares, cuando alguno de los valores se ubicó fuera del intervalo de los valores arriba señalados, fueron eliminados todo el conjunto de índices de ese sujeto. En menores de 5 años y escolares se consideraron como

datos válidos todos los valores de IMC que estuvieron entre 10.0 y 38.0 kg/m^2. En el caso de adolescentes, se consideraron como datos válidos para el análisis del índice de talla para la edad aquellos encontrados entre -5.5 y +3.0 puntuaciones Z, y para el IMC los datos entre 10.0 y 58.0 kg/m^2.

- ## Adultos

La evaluación del estado nutricional de la población adulta (igual o mayor a 20 años y hasta 59.9 años) se realizó mediante 2 índices: el índice de masa corporal (IMC) y la circunferencia de cintura. La Tabla 3.2 muestra los indicadores y puntos de corte sugeridos para el IMC en adultos por la Organización Mundial de la Salud [WHO, 2000].

Tabla 3.2 Indicadores para evaluar la presencia de sobrepeso y obesidad de acuerdo al IMC en adultos. OMS, 2000.

Indicadores para el IMC	Punto de Corte
Bajo peso	<18.50
Delgadez leve	17.00 a 18.49
Delgadez moderada	16.00 a 16.99
Delgadez severa	< 16.00
Normal	18.50 a < 24.99
Sobrepeso o pre-obesidad	≥ 25.00
Obesidad	≥ 30.00

WHO. (2006). WHO Child Growth Standards: Methods and development: Length/height-for-age, weight-for-age, weight-for-length, weight-for-height and body mass index-for-age. Geneva: World Health Organization.

Los valores de IMC incluidos como válidos fueron todos aquellos entre 10.00 y 58.00 kg/m^2. Los valores válidos de estatura se consideraron entre 130.0 y 200.0 cm. Para el análisis de la circunferencia de cintura se empleó el criterio propuesto por la Federación Internacional de Diabetes [IDF, 2012]. Se consideró como obesidad abdominal una circunferencia de cintura mayor a 80.0 cm en mujeres y mayor a 90.0 cm en hombres. Los valores válidos de cintura se consideraron entre 50.0 y 180.0 cm para ambos sexos.

- **Adultos mayores**

La evaluación del estado nutricional en la población de 60 años y más se realizó empleando 2 indicadores que fueron la presencia de bajo peso y sobrepeso mediante el IMC.

Los puntos de corte para definir un peso bajo o sobrepeso con el IMC en adultos jóvenes, y que son sugeridos por la OMS, son cuestionados para su aplicación en adultos mayores. A la fecha, no hay acuerdo en cuanto a un punto de corte universal de IMC para adultos mayores. Por lo tanto, cualquier sugerencia de clasificación de bajo peso o sobrepeso en adultos mayores debe interpretarse con precaución. El IMC tiene distinto significado en los adultos mayores que en los adultos jóvenes a causa de la edad y los efectos sobre la reducción de la estatura, la redistribución de grasa corporal y disminución de la masa muscular [WHO, 1995]. Esto es particularmente cierto en adultos mayores de 70 años o más donde la delgadez implica un riesgo mayor de mortalidad que el sobrepeso [WHO, 1995; Dylan, 2005]. Varios estudios sugieren que cuando el IMC es menor al intervalo de 19.00 a 25.00 se incrementa el riesgo de mortalidad en adultos con 65 años o más [WHO, 1995; A Rissanen, 1991; Jan-Magnus, 2012; Breeze, 2006].

Los mismos estudios sugieren que el IMC saludable puede encontrarse entre 25.00 y 29.99 en este grupo de edad. En 1995, la OMS sugirió preliminarmente que se podría clasificar con bajo peso a los adultos mayores de 60 a 69 años si presentan un IMC menor a 18.50 y con sobrepeso si presentan un IMC mayor o igual a 30.00 [WHO, 1995]. El bajo peso y el sobrepeso en adultos mayores de 60 a 69 años se definió cuando tuvieran respectivamente un IMC de menor a 18.50 ó mayor o igual a 30.00 de acuerdo a los puntos de corte sugeridos preliminarmente por el grupo de expertos de la OMS [WHO 1995]. En las personas de 70 y más años el bajo peso se definió con un IMC menor a 25.00, y el sobrepeso con un IMC mayor o igual a 30.00 [Jan-Magnus, 2012; Breeze 2006]. Los valores de IMC incluidos como válidos fueron todos aquellos entre 10.00 y 58.00 kg/m^2.

Resultados

Menores de 5 años

En los menores de 5 años el 8.6% presenta baja talla, 5.6% bajo peso, 5.1% desnutrición aguda (emaciación) y 10.8% sobrepeso (Tabla 3.3). La prevalencia de baja talla en niñas es 4.4% menor a la de niños. La desnutrición aguda en Nuevo León permanece estable y la desnutrición crónica (baja

talla) se redujo de 15.2% en el 2000 a 8.6% en el 2012, lo que representa una disminución de 6.6% (Figura 3.1). De igual forma, la prevalencia de sobrepeso y obesidad descendió 3.0% del 2000 al 2012. La población estudiada de niños menores de 5 años fue de 947 niños que representan a 212,477 preescolares.

Tabla 3.3 Prevalencia de talla baja y sobrepeso en menores de 5 años, dividido por género.

Condición	Masculino Presentan la condición			Femenino Presentan la condición			Ambos sexos Presentan la condición		
	F	%	IC95%	F	%	IC95%	F	%	IC95%
Baja talla (<-2 z Talla/edad)	13,000	10.5	8.1, 13.5	5,368	6.1	4.2, 8.7	18,368	8.6	7, 10.6
Bajo peso (<-2z Peso/edad)	6,480	5.2	3.5, 7.5	5,454	6.2	4.2, 8.7	11,934	5.6	4.3, 7.2
Emaciación (<-2 z Peso/talla)	6,495	5.1	3.5, 7.5	4,603	5.1	3.5, 7.7	11,098	5.1	3.9, 6.8
Sobrepeso (>2 z IMC/edad)	14,951	12.1	9.5, 15.2	7,896	8.9	6.7, 12.1	22,847	10.8	9, 12.9

Fuente: EESN-NL 2011/2012 (Cuestionario Hogar).

Figura 3.1. Prevalencia estatal de bajo peso, baja talla, emaciación y sobrepeso en menores de cinco años de edad.

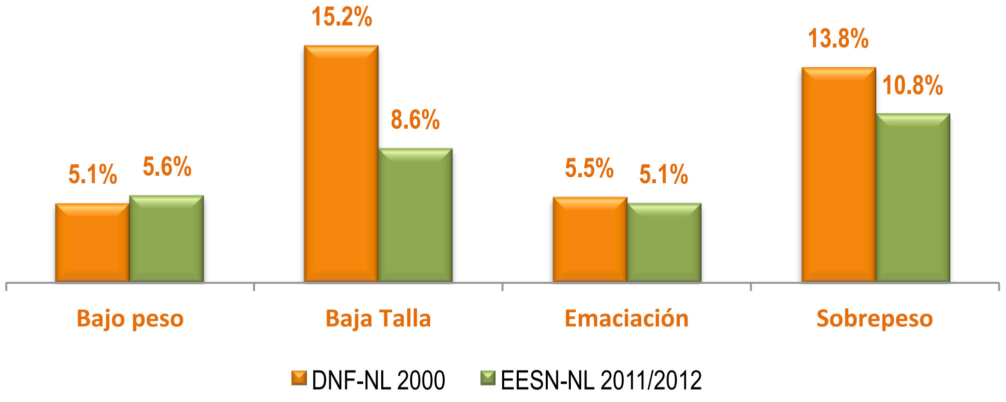

Fuente: EESN-NL 2011/2012 (Cuestionario Hogar).
DNF-NL=Diagnóstico Nutriológico de las Familias y Menores de 5 años de Nuevo León 2000.
EESN-NL=Encuesta Estatal de Salud y Nutrición de Nuevo León 2011/2012.

Los datos por región en el estado nutricio de los menores de cinco años muestran que la presencia de desnutrición crónica es mayor en la Región Sur, la presencia de desnutrición aguda (emaciación) es mayor en la Región Norte y que el sobrepeso tiene mayor presencia en la Zona Metropolitana (Tabla 3.4; Figura 3.2).

Tabla 3.4 Prevalencia de talla baja y sobrepeso en menores de 5 años, divididos por región geográfica.

Condición	Región											
	Metropolitana			Norte			Sur			Centro		
	F	%	IC95%	F	%	IC95%	F	%	IC95%	F	%	IC95%
Baja talla (<-2 z Talla/edad)	12,475	7.6	4.9, 12.4	1,589	13.0	8.8, 18.7	1,152	16.6	13.0, 20.8	3,287	10.2	6.9, 15.0
Bajo peso (<-2z Peso/edad)	9,096	5.6	3.0, 9.4	930	7.6	4.3, 12.2	269	3.9	2.4, 6.6	1,697	5.3	2.9, 8.9
Emaciación (<-2 z Peso/talla)	7,999	4.9	2.7, 8.8	1,244	10.2	6.5, 15.5	434	6.3	4.2, 9.3	1,349	4.2	2.2, 7.8
Sobrepeso (>2 z IMC/edad)	14,449	8.9	5.7, 13.5	659	5.4	3.1, 10.1	403	5.8	3.7, 8.6	2,409	7.5	4.6, 11.7

Fuente: EESN-NL 2011/2012 (Cuestionario Hogar).

Figura 3.2 Prevalencia estatal de bajo peso, baja talla, emaciación y sobrepeso en menores de cinco años de edad, divididos por región geográfica

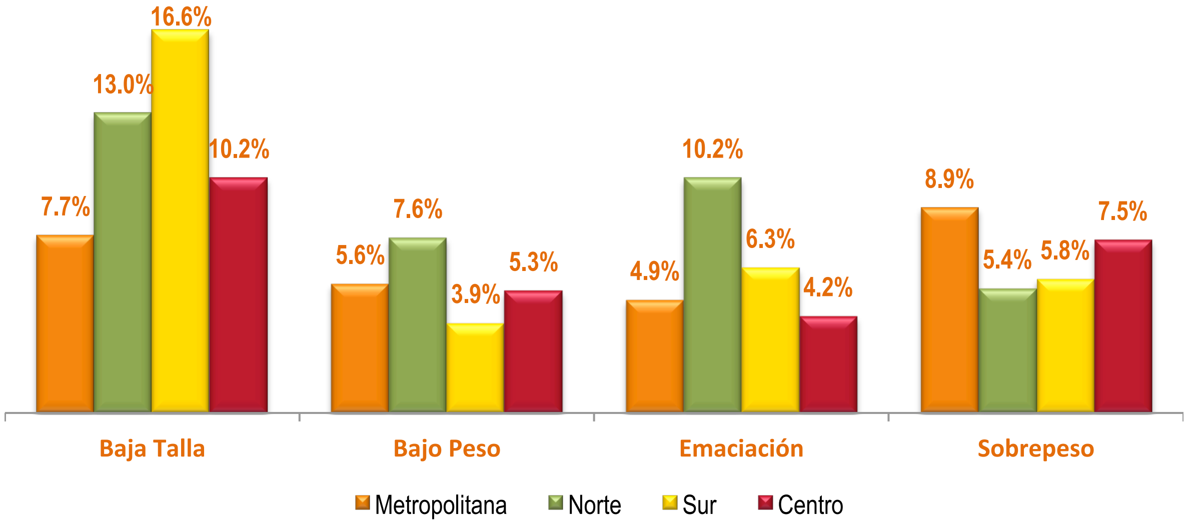

Fuente: EESN-NL 2011/2012 (Cuestionario Hogar).

Niños de 5 a 11 años de edad

El sobrepeso y la obesidad afectan a 33.9% de los niños de 5 a 11 años de Nuevo León (Tabla 3.5. La prevalencia de sobrepeso y obesidad es 0.6% mayor en mujeres que en hombres, no obstante, la obesidad afecta más a los varones con 19.4%, comparada con 17.6% en las mujeres. Desde el 2000 al 2012 la presencia de sobrepeso y la obesidad se incrementó 4.5% (Figura 3.3).

La población estudiada en niños escolares de 5 a 11 años fue de 1,561 que al aplicar los factores de expansión representan a 442,425 escolares.

Tabla 3.5 Prevalencia de talla baja, sobrepeso y obesidad en niños de 5 a 11 años. EESN-NL 2011/2012.

Condición	Masculino			Femenino			Ambos sexos		
	Presentan la condición			Presentan la condición			Presentan la condición		
	F	%	IC95%	F	%	IC95%	F	%	IC95%
Baja talla (<-2 z Talla/edad)	11,822	5.5	4.1, 7.3	14,251	7.1	5.5, 9.2	26,073	6.3	5.2, 7.6
Delgadez (<-2 z IMC/edad)	12,171	5.6	4.2, 7.4	5,572	2.8	1.8, 4.2	17,743	4.3	3.3, 5.3
Sobrepeso (>1 z IMC/edad)	30,993	14.4	12.2, 17.0	33,300	16.5	14.0, 19.3	64,293	15.4	13.7, 17.3
Obesidad (>2 z IMC/edad)	41,797	19.4	16.8, 22.3	35,428	17.6	15.0, 20.4	77,225	18.5	16.7, 20.5

Fuente: EESN-NL 2011/2012 (Cuestionario Hogar).

Figura 3.3. Prevalencia de sobrepeso y obesidad en escolares de 5 a 11 años por género.

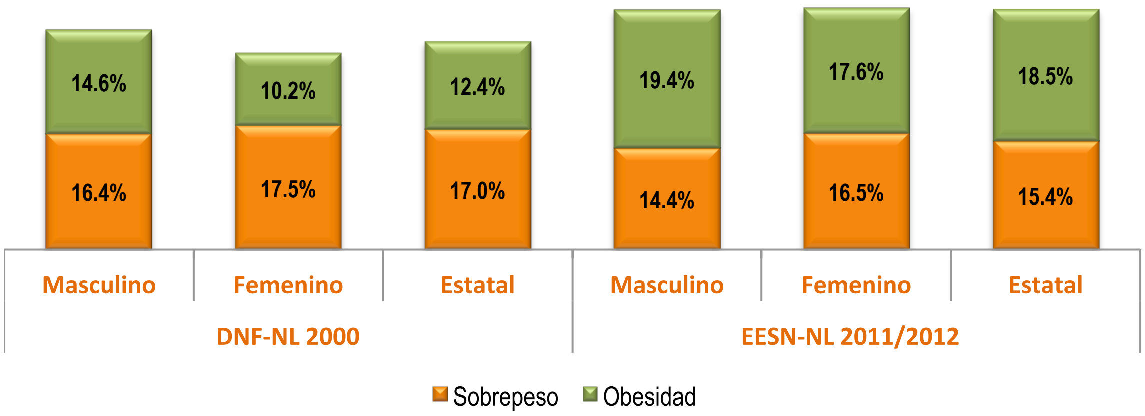

Fuente: EESN-NL 2011/2012 (Cuestionario Hogar).
DNF-NL=Diagnóstico Nutriológico de las Familias y Menores de 5 años de Nuevo León 2000.
EESN-NL=Encuesta Estatal de Salud y Nutrición de Nuevo León 2011/2012.

Tabla 3.6 Prevalencia de talla baja, sobrepeso y obesidad en niños de 5 a 11 años, dividido por región geográfica.

Condición	Región											
	Metropolitana			Norte			Sur			Centro		
	F	%	IC95%	F	%	IC95%	F	%	IC95%	F	%	IC95%
Baja talla (<-2 z Talla/edad)	20,890	6.3	4.3, 9.2	3,302	6.5	4.4, 9.7	971	5.0	3.1, 8.5	892	7.6	5.7, 10.2
Delgadez (<-2 z IMC/edad)	8,642	2.6	1.4, 4.6	7,369	14.5	11.3, 18.8	659	3.4	1.8, 6.2	1,201	10.3	8, 13.1
Sobrepeso (>1 z IMC/edad)	54,803	16.4	13.2, 20.5	4,732	9.3	6.6, 12.7	2,440	12.5	9.1, 17	1,328	11.3	8.9, 14.3
Obesidad (>2 z IMC/edad)	62,611	18.8	15.2, 22.9	9,346	18.4	14.7, 22.8	3,535	18.1	13.9, 23	1,449	12.4	9.9, 15.5

Fuente: EESN-NL 2011/2012 (Cuestionario Hogar).

Los datos por región en el estado nutricio de los escolares muestran que la presencia de sobrepeso y obesidad son mayores en la región Metropolitana. La presencia de sobrepeso es menor en la Región Centro y la de obesidad en la Región Sur (Tabla 3.6; Figura 3.4).

Figura 3.4 Prevalencia de sobrepeso y obesidad en escolares de 5 a 11 años. Datos por región.

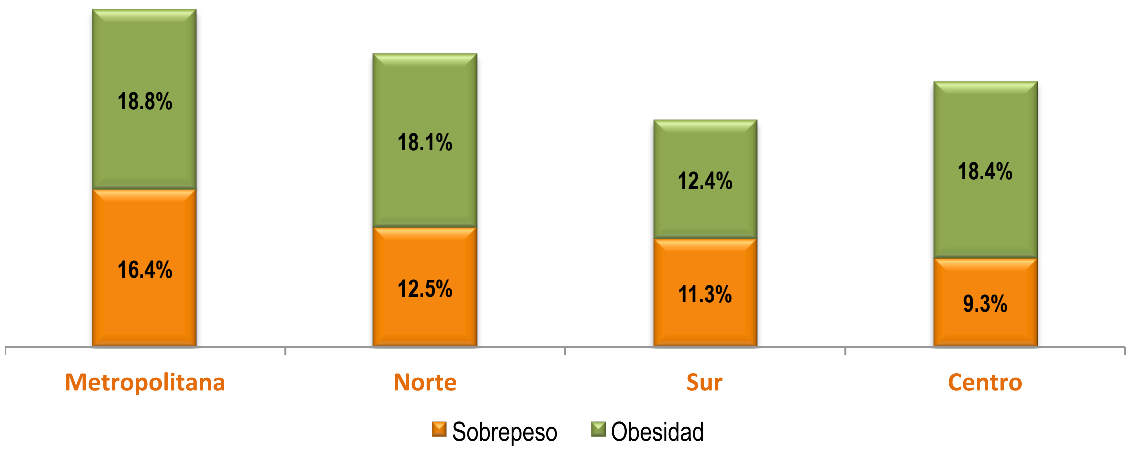

Fuente: EESN-NL 2011/2012 (Cuestionario Hogar).

Adolescentes.

Los resultados de la EESN-NL 2011/2012 muestran que 39.3% de los adolescentes de 12 a 19 años presentan sobrepeso y obesidad (Tabla 3.7). La prevalencia de varones con sobrepeso y obesidad es 9.1% mayor que la de mujeres. La presencia de sobrepeso y obesidad en el Estado se incrementó de 28.1% en el 2000 a 39.3% en el 2012; lo que representa un aumento de 11.2% (Figura 3.5). La población estudiada en niños adolescentes o púberes de 12 a 19 años fue de 1,189 que al aplicar los factores de expansión representan a 390,350 individuos.

Tabla 3.7 Prevalencia de baja talla, sobrepeso y obesidad en adolescentes de 12 a 19 años.

Condición	Masculino			Femenino			Ambos sexos		
	Presentan la condición			Presentan la condición			Presentan la condición		
	F	%	IC95%	F	%	IC95%	F	%	IC95%
Baja talla (<-2 z Talla/edad)	7,469	3.5	2.4, 5.2	9,903	4.8	3.5, 6.6	17,372	4.1	3.2, 5.3
Delgadez (<-2 z IMC/edad)	14,036	6.6	4.9, 8.7	16,576	8.0	6.1, 10.1	30,612	7.3	6.0, 8.8
Sobrepeso (>1 z IMC/edad)	46,826	22.0	19.0, 25.4	48,424	23.3	20.3, 26.6	95,250	22.6	20.5, 24.9
Obesidad (>2 z IMC/edad)	46,412	21.8	18.7, 25.0	23,663	11.4	9.3, 14.0	70,075	16.7	14.8, 18.7

Fuente: EESN-NL 2011/2012 (Cuestionario Hogar).

Figura 3.5 Prevalencia de sobrepeso y obesidad en adolescentes de 12 a 19 años por género.

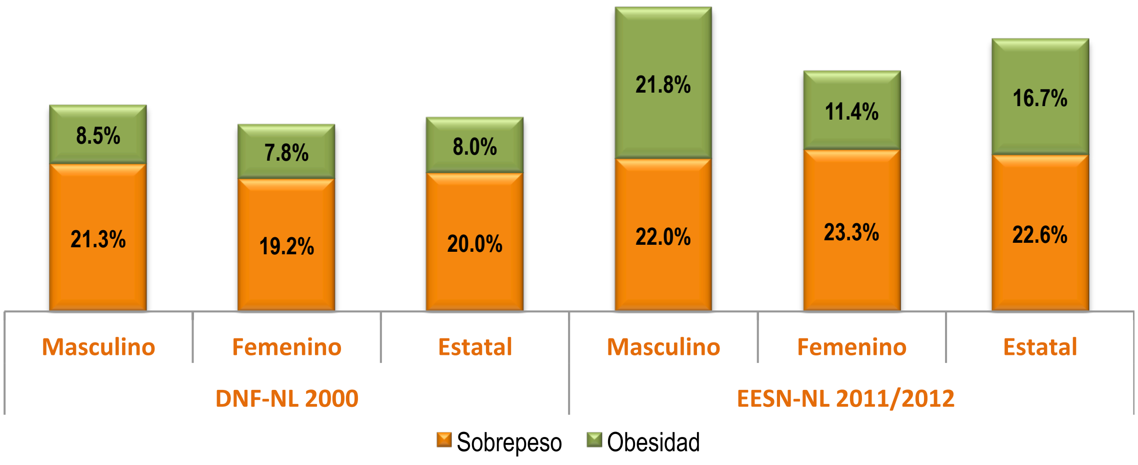

Fuente: EESN-NL 2011/2012 (Cuestionario Hogar).
DNF-NL=Diagnóstico Nutriológico de las Familias y Menores de 5 años de Nuevo León 2000.
EESN-NL=Encuesta Estatal de Salud y Nutrición de Nuevo León 2011/2012.

En el grupo de adolescentes de 12 a 19 años de edad los resultados por región muestran una mayor prevalencia de sobrepeso en la Región Metropolitana y de obesidad en la Región Norte. La menor prevalencia de sobrepeso y obesidad se presentó en la Región Sur (Tabla 3.8; Figura 3.6).

Tabla 3.8 Prevalencia de talla baja, sobrepeso y obesidad en adolescentes de 12 a 19 años, dividido por región geográfica.

Condición	Región											
	Metropolitana			Norte			Sur			Centro		
	F	%	IC95%	F	%	IC95%	F	%	IC95%	F	%	IC95%
Baja talla (<-2 z Talla/edad)	15,784	4.3	2.7, 6.8	849	4.6	2.7, 8.1	819	9.7	7.3, 12.8	3,063	7.4	4.8, 11.1
Delgadez (<-2 z IMC/edad)	58,492	15.9	12.7, 19.9	2,447	13.2	9.5, 17.8	1,149	13.6	10.6, 17.0	8,135	19.6	15.3, 24.7
Sobrepeso (>1 z IMC/edad)	85,749	23.3	19.4, 27.8	3,669	19.8	15.4, 25.2	1,736	20.5	17.0, 24.4	7,963	19.2	14.9, 24.3
Obesidad (>2 z IMC/edad)	61,677	16.8	13.4, 20.8	3,936	21.2	16.8, 26.9	575	6.8	4.8, 9.5	7,522	18.1	14.0, 23.1

Fuente: EESN-NL 2011/2012 (Cuestionario Hogar).

Figura 3.6 Prevalencia de sobrepeso y obesidad en adolescentes de 12 a 19 años, divididos por región geográfica.

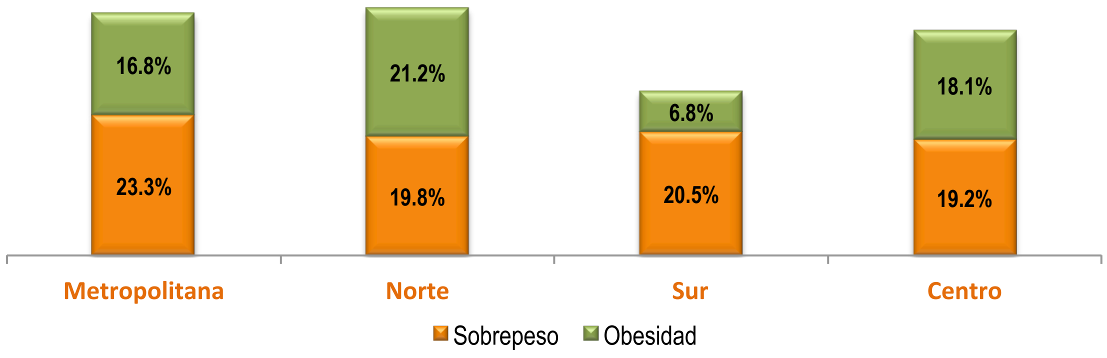

Fuente: EESN-NL 2011/2012 (Cuestionario Hogar).

Adultos

El sobrepeso y la obesidad afectan a 71.7% de la población adulta de 20 a 59 años de Nuevo León. Los proporción de hombres con esta condición es 3.1% mayor que en la mujeres (Tabla 3.9). En el 2000 la prevalencia de sobrepeso fue de 58.5% y se incrementó 13.2% hasta el 2012 (Figura 3.7).

Debido a que la presencia de obesidad abdominal representa un factor de riesgo importante para el desarrollo de enfermedades crónicas como la diabetes, en la EESN-NL 2011/2012 se consideró su evaluación a través de la medición de la circunferencia de cintura. La presencia de obesidad abdominal en los adultos de Nuevo León es de 72.4% (Figura 3.8). Las mujeres tienen 9.8% mayor prevalencia de obesidad abdominal que los hombres. La población estudiada de adultos de 20 a 59 años fue de 5,198 que al aplicar los factores de expansión representaron a 1, 513,193 individuos.

Tabla 3.9 Distribución porcentual del IMC en población adulta de 20 a 59 años. EESN-NL 2011/2012.

Condición	Masculino			Femenino			Ambos sexos		
	Presentan la condición			Presentan la condición			Presentan la condición		
	F	%	IC95%	F	%	IC95%	F	%	IC95%
Bajo peso	9,958	1.7	1.2, 2.3	15,743	1.7	1.3, 2.2	25,701	1.7	1.4, 2.1
Adecuado	147,581	24.8	23.0, 26.7	256,098	27.9	26.3, 29.5	403,679	26.7	25.5, 27.9
Sobrepeso	276,456	46.5	44.4, 48.6	290,341	31.6	30.0, 33.3	566,797	37.5	36.2, 38.8
Obesidad	160,547	27.0	25.2, 28.9	356,469	38.8	37.1, 40.5	517,016	34.2	32.9, 35.5
Obesidad abdominal	368,944	66.4	64.3, 68.4	675,900	76.2	74.6, 77.7	1,044,844	72.4	71.2, 73.6

Fuente: EESN-NL 2011/2012 (Cuestionario Hogar).

Figura 3.7. Prevalencia de sobrepeso y obesidad en adultos de 20 a 59 años por género.

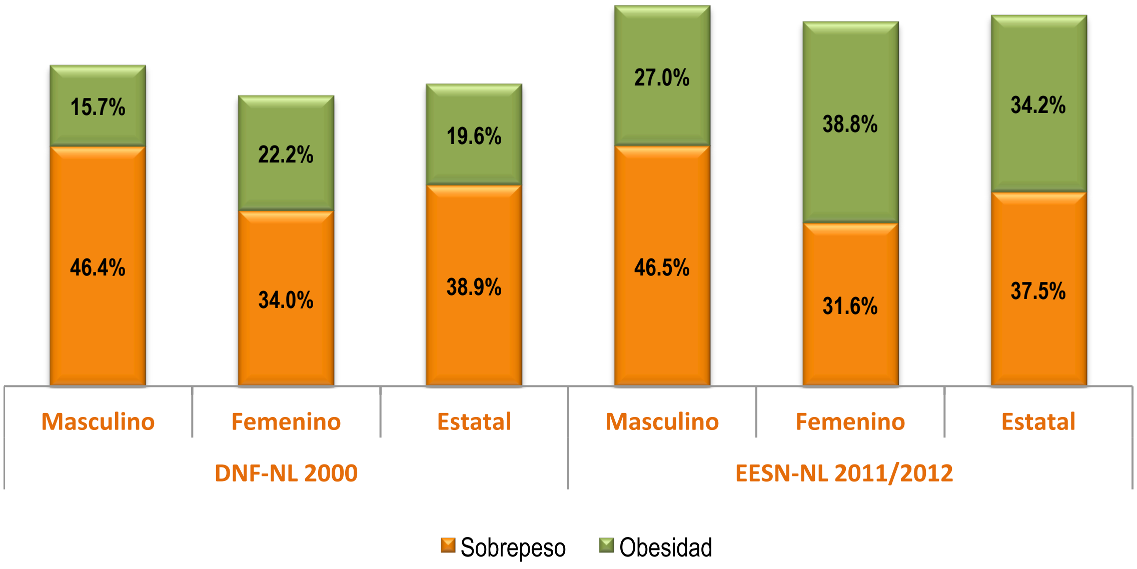

Fuente: EESN-NL 2011/2012 (Cuestionario Hogar)
DNF-NL=Diagnóstico Nutriológico de las Familias y Menores de 5 años de Nuevo León 2000.
EESN-NL=Encuesta Estatal de Salud y Nutrición de Nuevo León 2011/2012.

Figura 3.8 Prevalencia de obesidad abdominal en adultos de 20 a 59 años por género.

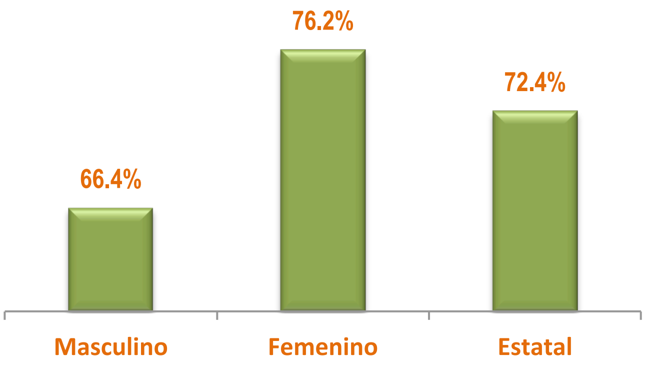

Fuente: EESN-NL 2011/2012 (Cuestionario Hogar).

En el grupo de adultos de 20 a 59 años de edad los datos por región muestran que la Región Sur presenta la mayor prevalencia de sobrepeso y la Región Norte la mayor proporción de sujetos con obesidad (Tabla 3.10; Figura 3.9). La prevalencia de obesidad abdominal es mayor en la Región Norte y la menor se encontró en la Región Sur (Figura 3.10).

Tabla 3.10. Distribución porcentual del IMC en la población adulta de 20 a 59 años, divididos por región geográfica.

Condición	Región											
	Metropolitana			Norte			Sur			Centro		
	F	%	IC95%	F	%	IC95%	F	%	IC95%	F	%	IC95%
Bajo peso	20,768	1.7	1.1, 2.5	1,294	1.6	1.0, 2.5	914	2.9	2.1, 3.8	2,726	1.5	1.0, 2.4
Adecuado	318,927	26.1	23.9, 28.6	20,536	25.4	22.9, 28.0	10,771	33.7	31.3, 36.0	53,444	29.9	27.4, 32.6
Sobrepeso	467,039	38.2	35.6, 40.9	28,921	35.7	33.0, 38.5	12,162	38.0	35.6, 40.5	58,674	32.8	30.2, 35.5
Obesidad	414,773	34.0	31.5, 36.6	30,228	37.3	34.6, 40.2	8,145	25.5	23.4, 27.7	63,822	35.7	33.1, 38.5
Obesidad abdominal	841,151	68.9	66.3, 71.3	60,602	74.8	72.2, 77.2	20,797	65.0	62.6, 67.3	122,246	68.4	65.7, 71.0

Fuente: EESN-NL 2011/2012 (Cuestionario Hogar).

Figura 3.9 Prevalencia de sobrepeso y obesidad en adultos de 20 a 59 años. Datos por región.

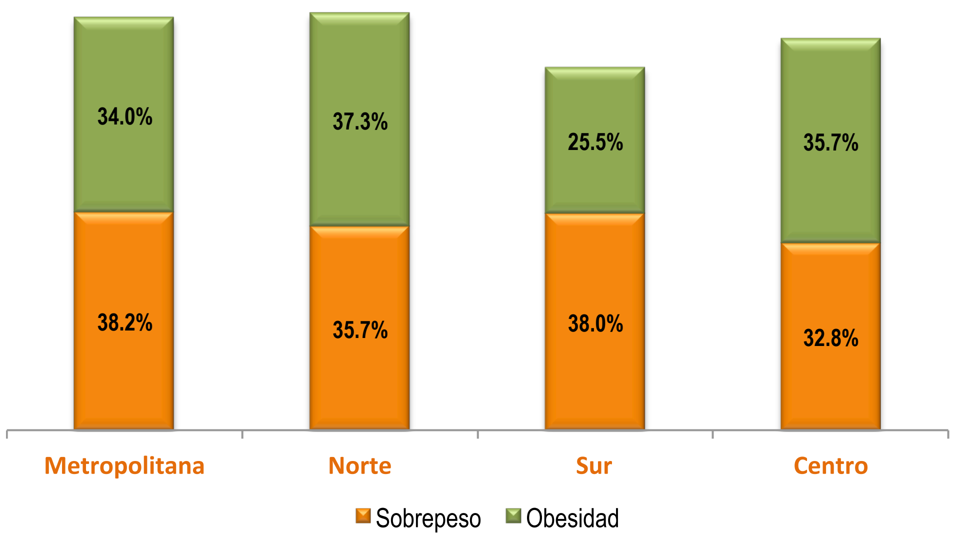

Fuente: EESN-NL 2011/2012 (Cuestionario Hogar).

Figura 3.10 Prevalencia de obesidad abdominal en adultos de 20 a 59 años. Datos por región.

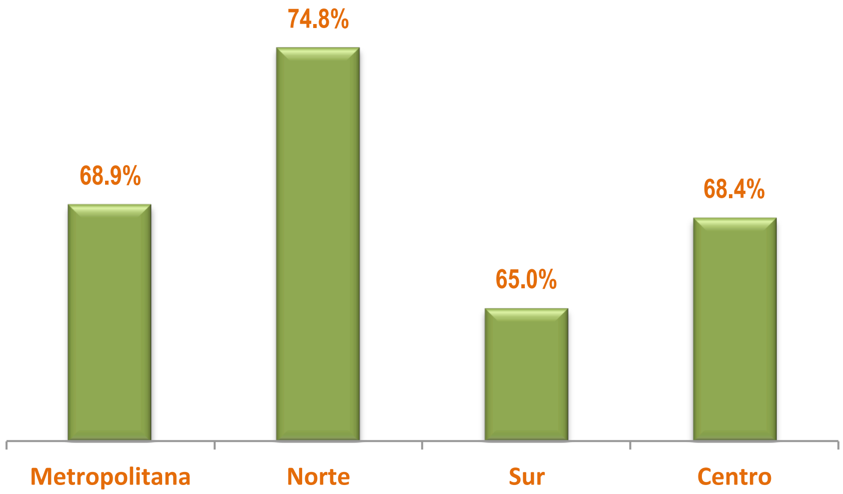

Fuente: EESN-NL 2011/2012 (Cuestionario Hogar).

Adultos mayores

La población adulta mayor de 60 años de Nuevo León presenta 32.9% de sobrepeso y 16.1% de bajo peso. El sobrepeso afecta más a las mujeres con 39.2%, que a los hombres con 24.4%. El bajo peso se encontró en hombres en 17.9% y en las mujeres en 14.7% (Tabla 3.11; Figura 3.11). Esta es la primera vez que se presentan en una encuesta de nutrición en México datos por separado de la situación antropométrica nutricional de los adultos mayores considerando los puntos de corte y una clasificación más apropiada para este grupo de edad. En el Diagnóstico Nutriológico de las Familias y Menores de 5 años de Nuevo León del 2000, los adultos mayores no tuvieron una representatividad suficiente por lo que no es posible realizar una estimación precisa del cambio de su situación nutricional en los últimos años. La población estudiada del grupo de 60 años y más fue 1,937 personas, las cuales representan a 430,257 individuos.

Tabla 3.11 Distribución porcentual del IMC en población adulta mayor (60 años y más).

Condición	Masculino			Femenino			Ambos sexos		
	Presentan la condición			Presentan la condición			Presentan la condición		
	F	%	IC95%	F	%	IC95%	F	%	IC95%
Bajo peso	32,798	17.9	15.4, 20.6	36,976	14.7	12.8, 17.0	69,774	16.1	14.5, 17.8
Peso adecuado	105,807	57.7	54.3, 61.0	115,509	46.1	43.2, 49.0	221,316	51.0	48.8, 53.2
Sobrepeso	44,791	24.4	21.6, 27.5	98,206	39.2	36.4, 42.1	142,997	32.9	30.9, 35.1

Fuente: EESN-NL 2011/2012 (Cuestionario Adultos de 60 años y más).

Figura 3.11. Prevalencia de bajo peso y sobrepeso en adultos mayores de Nuevo León.

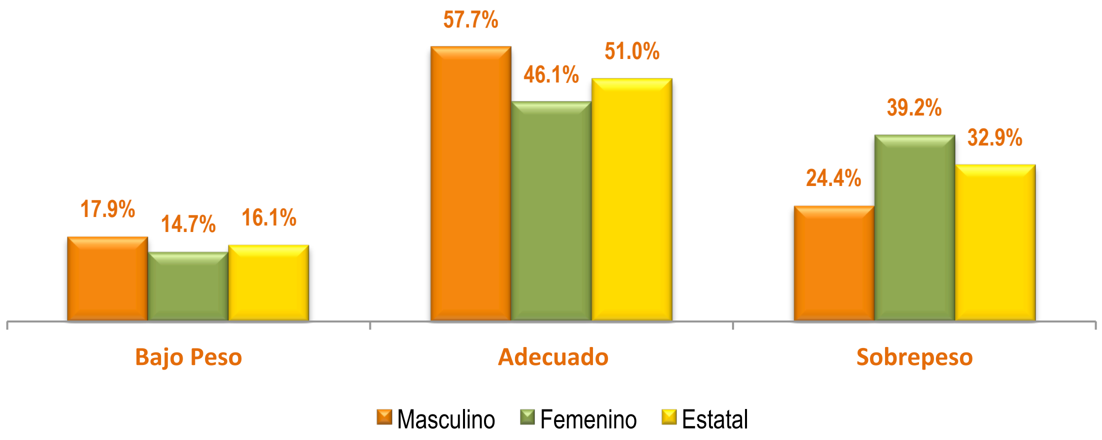

Fuente: EESN-NL 2011/2012 (Cuestionario Adultos de 60 años y más).

En el grupo de adultos mayores los resultados por región muestran que el bajo peso se presenta en mayor proporción en la Región Sur del Estado, mientras que el sobrepeso es más prevalente en la Región Norte (Tabla 3.12; Figura 3.12).

Tabla 3.12 Distribución porcentual del IMC en población adulta mayor (60 y más años), divididos por región geográfica.

Condición	Región											
	Metropolitana			Norte			Sur			Centro		
	F	%	IC95%	F	%	IC95%	F	%	IC95%	F	%	IC95%
Bajo peso	48,225	15.8	12.1, 20.0	6,143	16.2	13.3, 19.7	3,268	26.4	23.1, 29.9	10,895	18.0	14.8, 22.1
Adecuado	155,527	50.8	45.5, 56.2	17,072	45.1	40.9, 49.5	6,704	54.1	50.2, 57.9	30,625	50.7	46.0, 55.4
Sobrepeso	102,133	33.4	28.6, 38.7	14,608	38.6	34.5, 42.8	2,429	19.6	16.7, 22.8	18,850	31.2	27.0, 35.7

Fuente: EESN-NL 2011/2012 (Cuestionario Adultos 60 años y más).

Figura 3.12 Prevalencia de bajo peso y sobrepeso en adultos mayores, divididos por región geográfica.

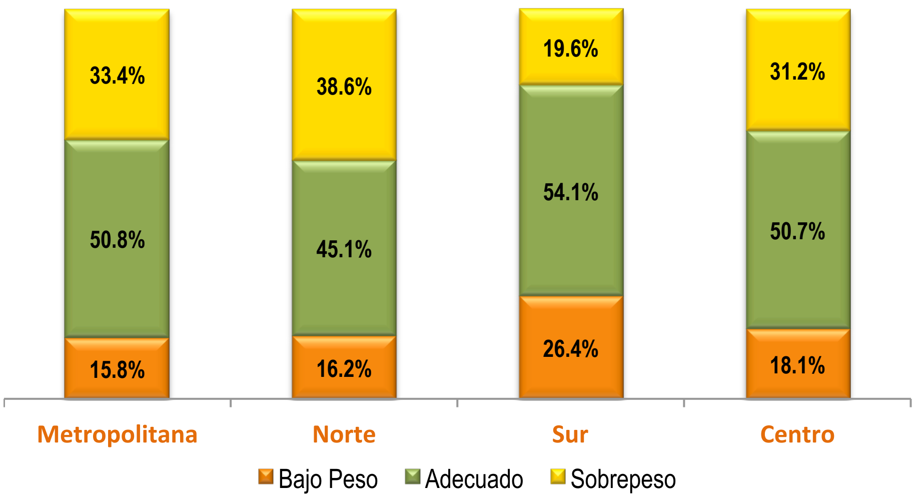

Fuente: EESN-NL 2011/2012 (Cuestionario Adultos 60 años y más).

Resultados relevantes

Niños menores de 5 años.

- En los menores de 5 años la desnutrición aguda es de 5.6% y ha permanecido estable durante los últimos 12 años. La desnutrición crónica es de 8.6% y tuvo un descenso importante de 6.6% del 2000 al 2012.
- La desnutrición aguda (bajo peso/edad) en menores de 5 años es 3.5% mayor al promedio nacional (2012). No obstante, la desnutrición crónica (baja talla/edad) es 5.0% menor que la media nacional.
- El porcentaje de niños menores de 5 años con sobrepeso y obesidad es de 10.8% y disminuyó 3.0% en los últimos 12 años.
- La prevalencia de sobrepeso y obesidad en los preescolares de Nuevo León es 1.1% mayor a la media nacional.

En niños de 5 a 11 años.

- La presencia de sobrepeso es de 15.4% y la de obesidad de 18.5%.
- La prevalencia de sobrepeso disminuyó 1.6% y la de obesidad se incrementó 6.5% en los últimos 12 años.
- El sobrepeso afecta en mayor proporción a las niñas y la obesidad a los niños.
- El sobrepeso es 4.4% menor y la obesidad 3.9% mayor a la media nacional.

Adolescentes de 12 a 19 años.

- La presencia de sobrepeso en adolescentes es de 22.6% y la de obesidad de 16.7%. El sobrepeso se incrementó 2.6% y la obesidad 8.7% del 2000 a 2012.
- El sobrepeso afecta en mayor proporción a las niñas y la obesidad a los niños.
- En adolescentes el sobrepeso es 1.0 % superior y la obesidad 3.4% más alta que el promedio nacional.

Adultos de 20 a 59 años.

- La prevalencia de sobrepeso es de 37.5% y de obesidad 34.2%. Aunque el sobrepeso disminuyó 1.4% del 2000 al 2012, la obesidad se incrementó 14.6% en el mismo periodo.

- El sobrepeso es más presente en los hombres y la obesidad más en las mujeres.

- El sobrepeso en hombres es 3.9% mayor y la obesidad 0.2% superior al promedio nacional

- El sobrepeso en mujeres es 3.9% menor al promedio nacional pero la obesidad es 1.3% superior a la media nacional.

- La presencia de obesidad abdominal es de 72.4% y es 9.8% mayor en la mujeres que en hombres.

- La prevalencia de obesidad abdominal en hombres es 1.9% mayor a la media nacional y en mujeres es 6.6% menor que el promedio nacional.

Adultos mayores de 60 años y más.

- Para el grupo de adultos mayores, por primera vez una encuesta estatal en el país trata de forma particular la situación nutricional en este grupo poblacional. El 16.1% de los adultos mayores presenta bajo peso y el 32.9% sobrepeso.

- Los hombres se encuentran más afectados por el bajo peso y las mujeres por el sobrepeso.

Datos por Región

- En general, en todos los grupos de edad, las regiones Metropolitana y Norte presentan mayores proporciones de indicadores nutricionales relacionados con el exceso de peso, mientras que en la Región Sur se observa una mayor presencia de desnutrición.

Menores de 5 años

- Los datos por región muestran que los preescolares de la Región Sur son los más afectados por la desnutrición crónica.

- Hay una alta presencia de desnutrición aguda en el Norte mientras que en la Región Sur se presenta la menor prevalencia.

- La Región Centro, seguida de la Región Metropolitana son las más afectadas por la presencia de sobrepeso y obesidad.

Niños de 5 a 11 años

- El sobrepeso y la obesidad afectan más a la Región Metropolitana y en menor grado a la Región Centro.

Adolescentes de 12 a 19 años

- El sobrepeso afecta más a la Región Metropolitana y la obesidad a la Región Norte.

Adultos de 20 a 59 años

- El sobrepeso es más elevado en la Región Metropolitana y en la Región Sur.
- La obesidad tiene mayor presencia en la Región Norte.
- La obesidad abdominal afecta también más a la Región Norte.

Adultos Mayores de 60 años y más

- El bajo peso aqueja más a la Región Sur y el sobrepeso tienen mayor presencia en la Región Norte

Capítulo 4

Alimentación

EESN – NL 2011/2012

4. Alimentación

Alimentación en los diferentes grupos de edad

La alimentación adecuada, sin importar el grupo de edad, conduce a un buen estado de nutrición y, en buena medida, a un satisfactorio estado de salud; por el contrario, una alimentación defectuosa, en variedad y /o cantidad, puede llevar a alteraciones de tipo físico, funcional o psicológico, lo que puede favorecer la aparición de enfermedades [Ramos *et al*. 2009].

La Norma Oficial Mexicana NOM-043-SSA2-2012, que se refiere a la promoción para la salud en materia alimentaria, define a la alimentación como el "conjunto de procesos biológicos, psicológicos y sociológicos relacionados con la ingestión mediante el cual el organismo obtiene del medio los nutrimentos que necesita, así como las satisfacciones intelectuales, estéticas y socioculturales que son indispensables para una vida humana plena". Así mismo, la NOM-043 refiere las características de una dieta correcta y agrupa a los alimentos en tres grupos principales que son: a) verduras y frutas, b) cereales y tubérculos, leguminosas y c) alimentos de origen animal, los cuales componen el Plato del Buen Comer, el cual es utilizado en las campañas de orientación alimentaria [SS, 2006].

En el año 2000, el 50% o más de las familias consumía siete alimentos, los mayores porcentajes de la población consumían: tortilla de maíz (100.0%), aceite vegetal (71.6%), huevo entero (67.1%), bebidas de cola (64.4%), frijol (59.4%), leche (56.8%), azúcar (54.5%) y jitomate (50.4%). También, bajo el concepto de universalidad, según el cual para la construcción de la canasta básica alimentaria, se requiere que un alimento sea consumido por al menos el 35.0% de la población [Ramos, 2009], o bien, el 25.0% de la población [Menchú, 2002], se pueden agregar: carnes procesadas (44.0%), cebolla blanca (38.0%), papa (34.4%), arroz (29.3%) y pastas (28.4%).

En relación a la alimentación, cada grupo de edad tiene características y necesidades propias: la infancia lo requiere para el crecimiento, en el cual está comprendido el aumento de unidades metabólicas; en la adolescencia, tiene cambios morfológicos, psicológicos y metabólicos; la etapa adulta es el momento de introducir la dieta mixta y equilibrada; la etapa del adulto mayor, es un grupo muy heterogéneo en el cual el inicio de la etapa fisiológica no está muy definida [Martínez *et al*. 2002].

Se analizó en forma general y por grupos de edad el consumo y la frecuencia de 105 alimentos en los grupos de 2 a 9 y 10 a 19 años; de 106 alimentos en el grupo de 20 a 59 años, y de 30 alimentos en el grupo de 60 años y más.

El objetivo del capítulo es describir el consumo y la frecuencia del consumo de alimentos por grupos de edad en la población de Nuevo León.

Consumo de alimentos en Nuevo León

En los últimos 12 años se producen cambios importantes en los lugares que ocupan los alimentos según el porcentaje de la población que los consume; a excepción del azúcar y la tortilla, en todos los alimentos el porcentaje de la población que los consumió fue mayor en el año 2012 que en el año 2000.

La tortilla de maíz (todos los tipos) la consumen el 84.9%, sin embargo, al desagruparla se encontró que el 40.7% consume la tortilla de maíz comprada en tortillería, 20.3% la tortilla de nixtamal hecha en casa y 21.2% la tortilla de harina de maíz MASECA o MINSA hecha en casa.

En cuanto al agua sola y el aceite de maíz, las preguntas no fueron similares en ambas encuestas. En la Tabla 4.1 se muestran los alimentos de mayor consumo en Nuevo León.

Tabla 4.1 Los 20 alimentos que consume el mayor porcentaje de la población de Nuevo León 2000-2012.

Alimento	2000		2012	
	Lugar	%	Lugar	%
Tortilla de maíz	1	100.0	8*	84.9*
Aceite de maíz	2	71.6	-	-
Huevo entero	3	67.1	4	90.1
Bebidas de cola	4	64.4	13	75.1
Frijol	5	59.4	2	93.6
Leche	6	56.8	9	80.4
Azúcar	7	54.5	60	28.9
Jitomate	8	50.4	5	88.8
Carnes procesadas	9	44.0	11	75.9
Cebolla blanca	10	38.0	10	75.9
Papa	11	34.4	8	82.5
Arroz	12	29.3	1	94.4
Pastas	13	28.4	14	75.0
Pollo	14	24.8	6	87.7
Pan dulce	15	21.0	28	56.6
Galleta dulce	16	19.7	46	35.2
Carne (molida regular)	17	13.5	7**	84.1
Pan de caja	18	12.2	26	60.0
Zanahoria	19	11.7	16	71.5
Plátano	20	11.0	18	69.5
Agua sola	-	-	3	93.1

Fuente: EESN-NL 2011/2012 (Cuestionario Niños de 0 a 9 años, Adolescentes de 10 a 19 años, Adultos de 20 a 59 años y Adultos de 60 años y más).
*Todos los tipos de tortillas de maíz.
** En el 2012 todos los tipos de carne.

Consumo de alimentos en los diferentes grupos de edad en Nuevo León

En cuanto al consumo de alimentos en el grupo de edad de 0 a 23 meses el día anterior a la encuesta, los primeros lugares en el porcentaje de infantes que los consumen fueron frutas y verduras, agua simple, cereal y leguminosa, leche no materna, otros líquidos. Los porcentajes más bajos de infantes en cuanto a los alimentos que consumieron fueron el huevo, azúcar agregada a alimentos o bebidas, leche LICONSA, NUTRISANO (Tabla 4.2).

Tabla 4.2 Alimentos que consumió el día anterior a la encuesta la población de 0 a 23 meses.

Alimento	Sí %	No %
Frutas y verduras	90.9	9.1
Agua simple	85.8	14.2
Cereales y leguminosas	82.4	17.6
Leche no materna(1)	82.3	17.7
Líquidos(2)	81.1	18.9
Carnes	72.9	27.1
Misceláneos(3)	60.3	39.7
Otros líquidos(4)	59.4	40.6
Lácteos	50.0	50.0
Huevo	38.6	61.4
Azúcar agregada a alimentos o bebidas	9.7	90.3
Leche LICONSA	4.6	95.4
NUTRISANO(5)	0.6	99.4

Fuente: EESN-NL 2011/2012 (Cuestionario Niños de 0 a 9 años).
(1) Leche entera (no LICONSA)
(2) Agua endulzada, té, café, refresco, caldo de frijol, caldo de pollo o res, aguamiel.
(3) Frituras, galletas, dulces o pastelitos.
(4) Atole con agua o leche, otro cereal con agua o leche, café con leche, jugos de frutas,
(5) Papilla OPORTUNIDADES.

Referente a la alimentación complementaria en los encuestados de 0 a 23 meses, el 25.0% de este grupo de edad incorporan once alimentos de manera temprana en la dieta. El 75.0% inicia con leche LICONSA y NUTRISANO en el primer mes de vida, el 50.0% incluye el agua y otra leche en los primeros tres meses de vida. Las frutas y verduras el 50.0% de este grupo de edad las incorpora en los primeros cinco meses (Tabla. 4.3).

Tabla. 4.3 Alimentos que se consumieron como alimentación complementaria en la población de 0 a 23 meses de Nuevo León 2000-2001.

Alimento	Percentil 25	Percentil 50	Percentil 75
Agua simple	1	3	4
Otra leche	0	3	8
Leche LICONSA	0	0	0
Otros líquidos	3	6	7
Cereales y leguminosas	4	6	8
Frutas y verduras	3	5	7
Carnes	3	6	8
Huevo	3	7	12
Lácteos	3	6	9
NUTRISANO	0	0	0
Misceláneos	1	6	8

Fuente: EESN-NL 2011/2012 (Cuestionario Niños de 0 a 9 años).

Los 20 alimentos que consume el mayor porcentaje de la población de 2 a 9 años, están conformados por cinco alimentos que pertenecen al de carne, huevo y embutidos; cada uno de los siguientes grupos como bebidas, verduras, dulces y botanas, leche y derivados, tiene dos alimentos, mientras que el grupo de frutas, comidas rápidas, misceláneos, tortilla de maíz y sopas participan con un alimento; los porcentajes de población que consumen estos alimentos van de 64.8% a 93.7%.

En cuanto a la frecuencia de consumo, los mayores porcentajes de población de este grupo de edad consumen diariamente, agua sola 85.1%, leche 68.3%, tortilla 66.6%, jitomate 56.7%, frijol 44.5%, refresco normal 40.2% (Tabla 4.4).

Tabla 4.4 Veinte alimentos que consume el mayor porcentaje de la población de 2 a 9 años.

Alimento	Consumo		Frecuencia semanal			
	Sí %	No %	1 día %	2 a 4 días %	5 a 6 días %	7 días %
Tortilla de maíz	100.0	0.0	7.7	21.4	4.3	66.6
Arroz	93.7	6.3	23.6	64.4	6.4	5.5
Huevo	93.7	6.3	18.4	49.0	11.1	21.5
Agua sola	91.5	8.5	3.3	7.2	4.4	85.1
Frijol	90.6	9.4	11.7	28.5	15.3	44.5
Pollo	90.5	9.5	37.4	54.1	5.4	3.2
Leche	88.4	11.6	5.7	18.2	7.8	68.3
Papa	87.3	12.7	37.6	54.7	3.8	3.8
Jitomate	84.4	15.6	12.6	21.3	9.4	56.7
Carne de res	83.5	16.5	40.7	49.1	5.6	4.5
Sopa de pasta	82.2	17.8	36.0	51.9	6.9	5.1
Refresco regular	80.2	19.8	15.1	39.1	5.7	40.2
Salchicha de puerco, pavo o combinado	78.9	21.1	40.5	52.2	3.6	3.6
Plátano	77.0	23.0	35.7	46.7	7.8	9.8
Jamón de puerco o de pavo o mortadela	76.0	24.0	43.1	47.5	4.4	5.0
Zanahoria	73.9	26.1	53.5	40.6	1.2	4.7
Frituras	71.9	28.1	35.3	42.0	8.5	14.2
Dulce	69.5	30.5	31.5	45.0	8.5	15.0
Mayonesa	67.6	32.4	52.5	40.1	3.7	3.7
Queso	66.1	33.9	46.8	44.2	4.7	4.3

Fuente: EESN-NL 2011/2012 (Cuestionario Niños de 0 a 9 años).

Los 20 alimentos que consume el mayor porcentaje de la población (66.1% al 93.5%) del grupo de edad de 10 a19 años están compuestos por cinco alimentos que pertenecen al grupo de carne, huevo y embutidos; misceláneos con tres alimentos; leche y derivados, bebidas, cereales y tubérculos con dos alimentos cada uno; leguminosas, verduras, dulces y botanas, sopas y tortilla, de maíz con un alimento. Referente a la frecuencia de consumo, los mayores porcentajes de la población de este grupo de edad consumen diariamente agua sola (80.9%), tortilla de maíz (66.6%), leche (56.1%), refresco normal (54.0%) y jitomate (51.6%); en el resto de los alimentos, el porcentaje de la población que los consume a diario es menor al 50.0% (Tabla 4.5).

Tabla 4.5 Veinte alimentos que consume el mayor porcentaje de la población de 10 a 19 años.

Alimento	Consumo		Frecuencia semanal			
	Sí %	No %	1 día %	2 a 4 días %	5 a 6 días %	7 días %
Tortilla de maíz	100.0	0.0	6.3	19.9	7.2	66.6
Agua sola	93.5	6.5	4.8	9.6	4.6	80.9
Arroz	93.5	6.5	25.5	51.2	8.5	8.4
Frijol	93.4	6.6	11.5	32.4	12.5	43.6
Huevo	88.0	12.0	16.8	46.3	16.6	20.3
Pollo	85.2	14.8	33.2	60.1	4.2	2.4
Carne de res	84.7	15.3	41.8	48.8	6.3	3.0
Refresco regular	83.4	16.6	9.7	27.2	9.1	54.0
Leche	82.1	17.9	10.9	25.2	7.7	56.1
Jitomate	80.4	19.6	12.3	30.0	6.1	51.6
Papa	78.2	21.8	33.8	37.4	4.2	2.8
Limón	77.2	22.8	31.1	55.6	8.1	15.2
Sopa de pasta	76.4	23.6	41.3	50.6	4.0	4.1
Jamón de puerco o de pavo o mortadela	76.1	23.9	47.0	41.0	4.9	7.0
Queso	73.9	26.1	41.2	45.9	3.3	9.5
Salchicha de puerco, pavo o combinado	73.2	26.8	49.4	45.2	3.3	2.1
Mayonesa	71.0	29.0	54.3	43.7	4.8	7.2
Frituras	69.0	31.0	31.31	48.23	6.64	13.83
Chiles	68.0	32.0	22.8	33.8	13.1	40.2
Pan blanco	67.2	32.8	30.7	31.0	2.3	3.2

Fuente: EESN-NL 2011/2012 (Cuestionario Adolescentes de 10 a 19 años).

En el grupo de edad de 20 a 59 años, las verduras y los misceláneos con cuatro alimentos cada uno; las frutas, leche y derivados, carne, huevo y bebidas con dos alimentos cada uno; sopas, tortilla de maíz y leguminosas con un alimento, que conforman el grupo de alimentos que el mayor porcentaje de población de este grupo de edad consume (60.8% al 94.8%).

Referente a la frecuencia diaria del consumo de los alimentos, los mayores porcentajes de la población se observan en agua sola 86.5%, tortilla de maíz 84.1%, chile 63.5%, en jitomate, leche, cebolla y refresco normal el porcentaje de consumo es de 50.6% a 59.7%. El resto de los alimentos los consume diariamente menos del 50.0% de la población (Tabla 4.6).

Tabla 4.6 Veinte alimentos que consume el mayor porcentaje de la población de 20 a 59 años.

Alimento	Consumo		Frecuencia semanal			
	Sí %	No %	1 día %	2 a 4 días %	5 a 6 días %	7 días %
Tortilla de maíz	100.0	0.0	2.9	7.9	5.2	84.1
Arroz	95.0	5.0	20.9	55.6	9.3	9.2
Frijol	94.8	5.2	8.6	32.9	12.4	46.0
Jitomate	94.0	6.0	13.6	18.8	7.9	59.7
Agua sola	93.5	6.5	4.0	4.9	5.5	85.6
Cebolla	85.2	14.8	22.3	25.6	7.9	54.2
Chile	84.4	15.6	16.3	20.6	9.6	63.5
Papa	82.5	17.5	31.6	44.0	3.7	3.2
Carne de res seca (machaca)	79.6	16.3	74.0	21.2	3.1	1.7
Queso	78.8	21.2	42.9	47.9	3.2	6.0
Leche	76.5	23.5	11.2	25.3	8.3	55.2
Lechuga	74.5	25.5	39.6	46.4	6.3	7.6
Zanahoria	72.6	27.4	51.4	41.2	2.1	5.3
Limón	72.5	27.5	30.5	51.6	6.9	21.0
Sopa de pasta	71.6	28.4	41.8	49.6	4.2	4.4
Aguacate	70.4	29.6	42.3	43.7	7.3	6.8
Refresco normal	69.6	30.4	11.2	28.9	9.3	50.6
Carne de puerco	69.6	27.0	74.5	23.5	0.9	1.0
Plátano	68.2	31.8	46.0	42.4	4.1	7.6
Naranja o mandarina	63.8	36.2	33.5	45.1	7.1	14.4

Fuente: EESN-NL 2011/2012 (Cuestionario Adultos de 20 a 59 años).

En el grupo de 60 años y más se estudió el consumo de treinta alimentos. El mayor porcentaje consume arroz 97.4% y el menor porcentaje 6.7% consume masa o nixtamal. Los alimentos que se consumen diariamente por más de 50.0% de la población de 60 años y más son: tortilla de maíz 82.4%, leche 67.7%, maíz 66.2%, grasa 64.3%, frijol 55.9%, azúcar 52.6% (Tabla 4.7).

Tabla 4.7 Frecuencia de consumo de alimentos en la población de 60 años y más.

Alimento	Consumo		Frecuencia semanal						
	Sí %	No %	1 día %	2 días %	3 días %	4 días %	5 días %	6 días %	7 días %
Arroz	97.4	2.6	8.0	18.6	29.4	11.0	10.7	4.0	18.3
Agua	97.0	3.0	6.4	12.9	25.8	16.3	16.6	12.1	9.8
Frijol	95.4	4.6	4.6	10.3	11.1	7.0	8.1	3.1	55.9
Frutas	94.5	5.5	9.7	18.9	14.9	6.9	8.2	3.0	38.4
Verduras	93.9	6.1	8.6	17.1	16.7	8.7	10.2	4.7	34.0
Papa	93.4	6.6	16.8	35.7	33.0	6.2	2.1	0.4	5.8
Pollo	92.3	7.8	22.5	32.1	31.4	8.0	2.3	0.8	3.0
Huevo	90.2	9.9	13.5	25.7	23.5	7.3	5.7	1.5	22.9
Leche	88.3	11.7	5.3	5.3	7.4	6.2	4.3	3.9	67.7
Tortilla	86.7	13.3	0.3	2.5	2.8	2.8	4.4	4.6	82.4
Grasa	84.0	16.0	4.9	11.1	6.8	4.1	6.9	1.9	64.3
Queso	81.6	18.4	27.6	29.0	24.6	4.6	3.6	1.7	8.8
Res o cerdo	80.7	19.3	31.9	33.6	24.5	4.4	2.4	0.2	3.0
Sopa de pasta	80.5	19.6	28.0	29.6	28.3	4.7	4.6	1.0	3.8
Refresco	73.5	26.5	14.6	12.1	9.8	4.4	7.1	2.2	49.7
Leguminosas	69.0	31.0	38.4	22.8	15.3	3.8	2.0	2.1	15.6
Galletas	68.0	32.0	28.5	28.8	19.3	3.1	2.8	1.6	16.0
Azúcar	65.0	35.0	4.7	13.0	15.4	4.0	7.1	3.2	52.6
Pan dulce	62.9	37.2	22.9	33.7	20.3	5.8	2.9	3.6	10.7
Pan blanco	59.5	40.5	40.1	30.5	18.5	2.5	2.8	0.7	4.9
Cereal	56.5	43.5	23.3	24.0	22.3	5.3	6.3	3.8	15.1
Embutidos	55.4	44.6	37.4	36.8	17.3	3.9	2.3	0.0	2.4
Pescado	54.4	45.6	65.3	18.9	13.7	0.3	0.6	0.9	0.3
Maíz	43.5	56.5	4.8	7.0	4.6	7.0	2.9	7.6	66.2
Pan de caja	42.9	57.1	32.0	30.4	23.0	6.1	5.1	1.0	2.5
Tortillas de trigo	37.3	62.7	35.8	33.3	18.6	3.0	1.5	0.9	6.8
Harina	30.7	69.3	37.8	27.1	16.0	4.6	1.5	1.5	11.6
Mariscos	14.9	85.1	66.8	23.7	9.0	0.0	0.0	0.0	0.5
Vísceras	9.3	90.7	55.3	25.0	13.6	3.0	3.0	0.0	0.0
Masa o nixtamal	6.7	93.3	22.7	18.2	4.5	3.4	4.5	4.5	42.0

Fuente: EESN-NL 2011/2012 (Cuestionario Adultos de 60 años y más).

Resultados relevantes

- En el diagnóstico nutriológico del año 2000, de los 452 alimentos que se consumían en Nuevo León, ocho alimentos los consumían el 50.0% o más de las familias, lo que mostraba un número muy bajo de alimentos y por lo tanto, una probabilidad baja de que un porcentaje alto de familias consumieran una alta variedad de alimentos. Cuando se trató el tema de la construcción de la canasta básica alimentaria para Nuevo León en el año 2000, en base al concepto de la universalidad [Ramos, 2009], se agregaron dos alimentos más, lo que aun así, hacía que hubiera pocos alimentos para construirla.

- En 2012, la Encuesta Estatal de Salud y Nutrición de Nuevo León mostró que del 50.0% al 93.6% de la población consume 31 alimentos, entre los que están: tortilla, frijol, agua sola, huevo, jitomate, pollo, carne de res, leche, cebolla, jamón de puerco o de pavo o mortadela, salchicha de puerco, pavo o combinado, refresco normal, sopa de pasta, queso, zanahoria, limón, plátano, lechuga, chile, aguacate, mayonesa, naranja o mandarina, caldo de pollo o res o verduras, manzana o pera, torta o sándwich, longaniza de chorizo, calabacitas; y para la construcción de la canasta básica alimentaria, se pueden agregar 17 alimentos más como son: elote, yogurt, tomate verde, brócoli, dulce, atún y sardina, tortilla de trigo, pescado, chocolate, galletas dulces, jugos o aguas de frutas, café con azúcar. Lo anterior muestra que la variedad de la población ha aumentado en 23 alimentos y para la construcción de la canasta básica en 38 alimentos.

- La EESN NL 2011/2012 muestra que, de los 20 alimentos que consume el mayor porcentaje de la población, nueve de ellos (tortilla de maíz, frijol, leche, agua sola, queso, carne de res, arroz, papa, sopa de pasta) se presentan consistentemente en los cuatro grupos de edad estudiados; en tres grupos de edad el pollo, jitomate y el refresco normal, que los consume del 66.0% al 100.0% de los individuos que conforman dichos grupos de edad. La dieta básica de Nuevo León sigue estando compuesta por el maíz, el frijol y el trigo [Torres, 2007].

- Aunque a doce años de distancia, el 50% o más de la población consume 19 alimentos más, y que en total para la construcción de la canasta básica alimentaria estarían disponibles 48 alimentos más que en el año 2000, se tienen que analizar otros elementos como necesidades energéticas, hábitos alimentarios, calidad y variedad de los alimentos y precio de alimentos [Gobierno de Honduras, 2010].

- Para acatar la NOM 043, que refiere que en una dieta variada, cada tiempo de comida debe contener un alimento de cada uno de los tres grupos de alimentos, sería necesario que un alto porcentaje de la población consumiera regularmente un número mayor a 40 alimentos, provenientes de los tres grupos.

- Lo anterior puede llevar a la observación de que en 11 años, la variedad de alimentación en la población puede haber tenido un cambio, pero aún queda por revisar el grado de cumplimiento de la NOM 043.

Capítulo 5

Servicios de Salud

EESN – NL 2011/2012

5. Servicios de Salud

Hoy en día se contempla una mayor solicitud por parte de la sociedad para tener los beneficios que otorgan los servicios de salud. Esta manifestación no es desconocida, sin embargo, según transcurre el tiempo, el aumento se hace innegable, tanto en magnitud como en diversidad, simbolizando un desafío permanente para las instituciones que otorgan este servicio, y en particular para las administraciones gubernamentales, que son las los encargadas de la procuración de la salud de la comunidad, con el propósito de satisfacer una función social manifestada por necesidades y demandas de la ciudadanía en materia de salud. El presente apartado detalla los hallazgos más relevantes relacionados con la utilización de los servicios de salud, el Seguro Popular y los programas de ayuda alimentaria.

El derecho a la salud está incluido en numerosos tratados internacionales y regionales de derechos humanos y en las leyes de la mayoría de los países. Le corresponde al Estado brindar este servicio y generar condiciones en las cuales la población pueda vivir saludablemente.

El estudio de la utilización de los servicios de salud de orden institucional, sitúa la importancia del análisis desde la apreciación del usuario; así como la percepción de la necesidad y preferencia de la atención que permite observarla y la accesibilidad a la oferta existente de servicios médicos en el estado de Nuevo León, como también la pronta resolución de los problemas de salud de la población, y demuestra además evidencia de los requerimientos y la utilización de estos servicios, que está determinado por factores demográficos, socioeconómicos, calidad sanitaria del ambiente, educación y características familiares, entre otros.

El Seguro Popular es un seguro médico voluntario para los mexicanos, que representa una alternativa para aquellas personas que carecen de empleo o auto-empleadas, y que no se encuentran afiliados a ninguna institución de seguridad social. Este aseguramiento público impulsa el pago anticipado de las actividades de atención médica que se brindarán, para atenuar los desembolsos por eventos o enfermedades catastróficas en salud.

El gran desafío que afrontan las estructuras de gobierno en México hoy en día, es el mejoramiento de los niveles de bienestar de la población, sin embargo, un primer obstáculo que enfrentan las familias, particularmente las más pobres es el nutricional. Nuestro país y Nuevo León, cuentan con

un historial de programas y políticas públicas orientados a mejorar la nutrición de los grupos más vulnerables; en este contexto, se pone a prueba la respuesta social organizada de los diferentes sectores sociales para afrontar dicho problema a través de los programas de apoyo alimentario.

Patrones de utilización de servicios de salud

A continuación se describen los principales resultados de las preguntas relacionadas con los servicios de salud realizadas a 13,335 individuos que representan a 3, 955,761 sujetos del Estado de Nuevo León, de los cuales el 80.0% se localizan en el Región Metropolitana, 12.4% en la Región Centro, 5.5% en la Región Norte y 2.2% en la Región Sur.

En cuanto a las instituciones que brindan servicios de salud a la población, el 86.9% manifestó estar afiliado o recibir servicio médico por alguna institución; la Figura 5.1 muestra las instituciones que brindan estos servicios a la población. Por lo que respecta a la distribución por regiones, en la Sur y Norte, se encontraron los mayores porcentajes de afiliación por los entrevistados, con 94.9% y 90.4% respectivamente; mientras que en las regiones Metropolitana y Centro se observó un comportamiento similar al estatal (Figura 5.2).

Figura 5.1. Distribución de la población derechohabiente por institución de salud.

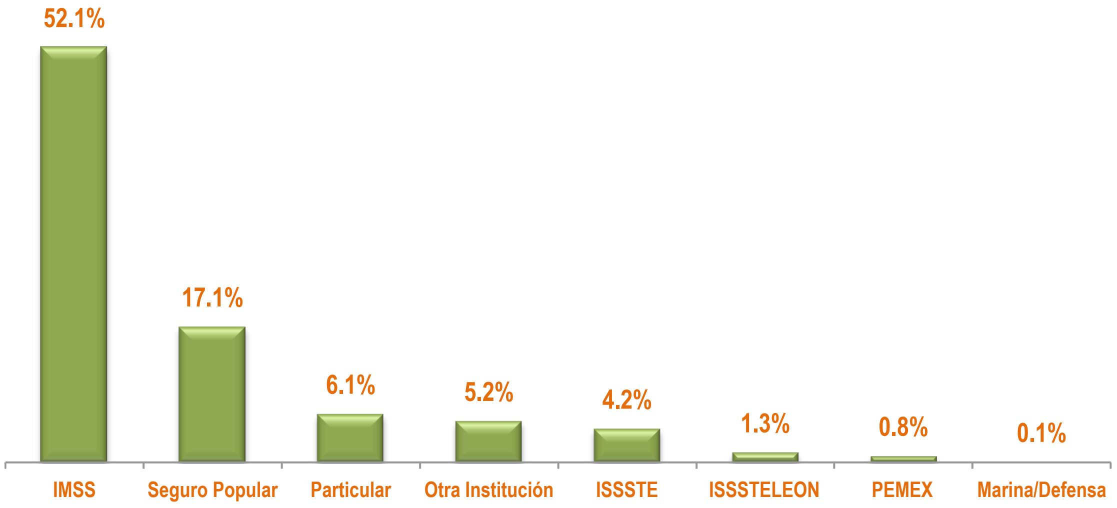

Fuente: EESN-NL 2011/2012 (Cuestionario Hogar).

Figura 5.2. Población afiliada a algún servicio de salud.

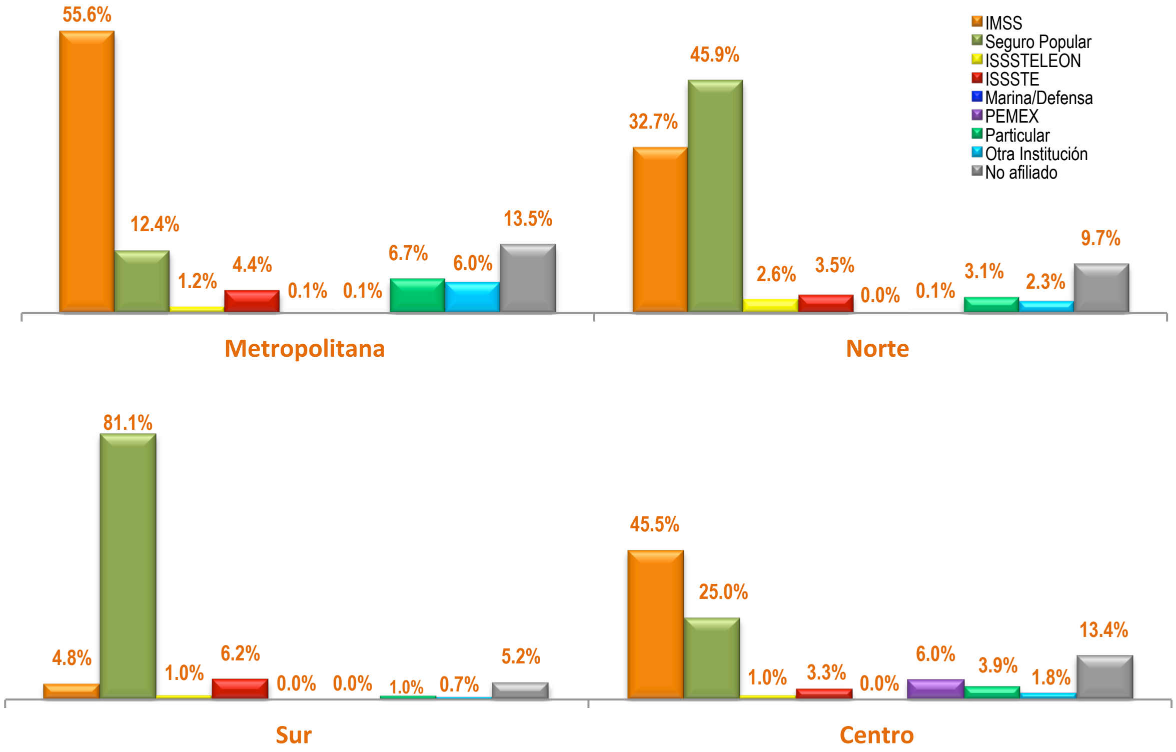

Fuente: EESN-NL 2011/2012 (Cuestionario Hogar).

Los datos arrojados por la EESN-NL 2011/2012 muestran un aumento en la adscripción al servicio médico en contraste con lo reportado en la ENSANUT 2006, en la que el 66.4% estaba afiliado, lo que representa un incremento de 20.5% en ese periodo (Tabla 5.1).

Tabla 5.1 Población según adscripción al servicio médico.

Instituciones	ENSANUT 2006 (Nuevo León)	EESN- NL 2011/2012
IMSS	55.3 %	52.1%
Seguro Popular	4.5 %	17.1%
Particular	2.5 %	6.1%
Otra institución	1.4 %	5.2%
ISSSTE	2.7%	4.2%
ISSSTELEÓN	SD	1.3%
PEMEX	SD	1.0%
Marina/Defensa	SD	0.0%

Fuente: EESN-NL 2011/2012 (Cuestionario Hogar).

Para las regiones Metropolitana y Centro la mayor adscripción de servicio médico es otorgada por IMSS, seguido del Seguro Popular; no siendo así para las regiones Sur y Norte, en las cuales el Seguro Popular alcanzó mayores porcentajes de adscripción, 81.1% y 45.9% respectivamente.

En relación a la fuente del servicio médico que ostenta la población, los resultados muestran que 44.5% está adscrito a través de un familiar, 42.2% por su trabajo, 10.5% por contratación propia y 2.7% por otros servicios como pensión y seguro de estudiante. En el contexto regional, las regiones Metropolitana, Norte y Centro, observan un comportamiento similar al estatal, no así la Región Sur donde el 75.3% recibe el servicio médico por el gobierno. (Tabla 5.2).

Tabla 5.2 Población según fuente del servicio médico, dividido por región geográfica.

Fuente del servicio médico	Región				Estatal
	Metropolitana	Norte	Sur	Centro	
	%	%	%	%	%
Trabajo	32.9	17.9	5.1	27.5	30.5
Familiar en el hogar y algún familiar	47.7	33.1	9.2	38.5	44.5
Muerte del asegurado	1.1	0.9	1.4	1.3	1.1
Ser estudiante	1.9	0.3	0.3	0.8	1.6
Contratación propia	9.6	20.5	8.8	11.6	10.5
Gobierno	6.7	27.3	75.3	20.4	11.7
Total	100.0	100.0	100.0	100.0	100.0

Fuente: EESN-NL 2011/2012 (Cuestionario Hogar).

Respecto a problemas de salud que los entrevistados manifestaron tener en las dos semanas previas a la encuesta, 20.4% declaró haberlos presentado, siendo las enfermedades respiratorias las más frecuentes: 7.4% presentó tos, catarro y/o dolor de garganta y 6.6% infecciones respiratorias a nivel estatal. Estos dos malestares de salud, prevalecen en todas las regiones según los encuestados (Tabla 5.3). De estos, el 52.6% manifestó haber sido atendido por un médico, 1.0% por un farmacéutico, 4.5% acudió a otras instancias (familiar, curandero, hierbero, acupunturista, entre otros) y 42.1% se autoatendió.

A nivel regional se observó que en las regiones Metropolitana, Centro y Norte los encuestados manifestaron ser atendidas por un médico, destacando la última región con un 80.5%, mientras que para la Región Sur el 74.2% manifestó no haber utilizado ningún recurso profesional médico (Tabla 5.4).

Tabla 5.3 Problemas de salud* presentes en las dos semanas previas a la encuesta, dividido por región geográfica.

Problemas de salud	Región				Estatal
	Metropolitana	Norte	Sur	Centro	
	%	%	%	%	%
Infecciones respiratorias y tos, catarro, dolor de garganta	66.2	36.8	42.1	27.8	62.5
Neumonía o bronconeumonía	1.0	0.0	2.6	0.5	0.9
Enfermedad pulmonar obstructiva crónica	1.1	1.1	2.6	0.5	1.1
Cáncer o tumores malignos	0.2	1.1	0.0	0.0	0.2
Tuberculosis	0.2	0.0	2.6	1.0	0.2
Enfermedades del corazón	0.9	0.0	2.6	0.5	0.8
Fiebre reumática	0.1	0.0	0.0	0.5	0.1
Infección de oído	0.4	0.0	0.0	0.5	0.4
Conjuntivitis	0.6	1.1	2.6	2.1	0.7
Asma	0.5	1.1	0.0	1.0	0.6
Diarrea	2.0	3.4	7.9	7.2	2.4
Enfermedades renales	0.2	1.1	0.0	0.5	0.3
Infección de vías urinarias	0.0	1.1	0.0	1.0	0.1
Gastritis o úlcera gástrica	1.5	3.4	2.6	4.6	1.8
Colitis	0.7	1.1	0.0	3.1	0.9
Parasitosis intestinal	0.6	1.1	0.0	0.5	0.6
Dolor de cabeza o cefalea sin manifestación	2.3	0.0	2.6	2.1	2.2
Fiebre sin otra manifestación	1.0	4.6	0.0	1.5	1.1
Enfermedad exantemática (varicela, rubéola escarlatina)	0.2	0.0	0.0	0.5	0.2
Hepatitis	0.1	0.0	0.0	0.0	0.1
Diabetes	1.7	8.0	5.3	5.7	2.2
Hipertensión arterial	2.0	10.3	13.2	9.3	2.9
Embolia o derrame cerebral	0.2	0.0	0.0	0.0	0.1
Artritis	0.3	1.1	0.0	1.0	0.3
Alergias	1.9	3.4	0.0	2.6	1.9
Problemas de la piel	0.5	1.1	0.0	0.0	0.5
Enfermedad bucodental	0.2	0.0	0.0	1.0	0.2
Dengue	0.2	0.0	0.0	0.0	0.1
Tabaquismo	0.0	0.0	0.0	0.5	0.0
Lesión física por accidente	1.3	1.1	5.3	2.6	1.4
Estrés	0.5	0.0	0.0	1.0	0.5
Depresión	0.3	0.0	0.0	1.0	0.3
Susto, empacho, mal de ojo o aire	0.2	0.0	0.0	0.5	0.2
Otro problema	10.4	17.2	5.3	18.0	11.0
Total	100.0	100.0	100.0	100.0	100.0

Fuente: EESN-NL 2011/2012 (Cuestionario Hogar).
*En la encuesta también fueron incluidos otros problemas de salud (obesidad, infección de transmisión sexual, VIH/SIDA, paludismo, intoxicación por veneno de alacrán, serpiente o araña, alcoholismo, padecimientos generados por consumo de drogas, lesión física por agresión, pérdida de la memoria), que no fueron referenciados por los encuestados.

Tabla 5.4 Población según tipo de recurso humano que atendió el problema de salud, dividido por región geográfica.

Recurso humano	Región				Estatal
	Metropolitana	Norte	Sur	Centro	
	%	%	%	%	%
Familiar	2.3	1.1	3.2	0.4	2.2
Amigo / Vecino	0.0	0.0	0.0	0.9	0.1
Farmacéutico	1.1	0.0	0.0	1.3	1.0
Curandero	0.0	1.1	0.0	0.0	0.0
Partera	0.0	1.1	0.0	0.0	0.0
Naturista	0.2	0.0	0.0	0.0	0.2
Médico	52.6	80.5	22.6	51.5	52.6
Dentistas	0.4	1.1	0.0	0.4	0.4
Otro*	1.9	0.0	0.0	0.4	1.7
Nadie	41.6	14.9	74.2	45.1	42.1
Total	100.0	100.0	100.0	100.0	100.0

Fuente: EESN-NL 2011/2012 (Cuestionario Hogar).
En la encuesta también fueron incluidos otros recursos humanos (Hierbero, homeópata, acupunturista, enfermera, encargado de comunidad/promotor/ auxiliar de salud), que no fueron referenciados por los encuestados.

Con respecto a la institución que atendió a los que manifestaron haber tenido un problema de salud, 35.3% expresó haber sido atendido en un servicio particular, 23.4% en el IMSS, 14.4% por el Seguro Popular/SSNL, 3.8% en el ISSSTE y 23.1% en otras instituciones; este comportamiento es semejante al que se observa en la Región Metropolitana, mientras que en la Región Sur las personas acuden preferentemente a los servicios médicos otorgados por el Seguro Popular y la Secretaría de Salud, en un orden del 75.0%. (Tabla 5.5).

Tabla 5.5 Población según institución de atención al problema de salud, dividido por región geográfica.

Instituciones	Región				Estatal
	Metropolitana	Norte	Sur	Centro	
	%	%	%	%	%
IMSS Oportunidades	2.8	1.3	0.0	4.8	2.8
IMSS	19.7	25.3	4.2	30.6	20.6
SSA	3.3	18.7	37.5	21.8	5.9
Seguro Popular	6.3	30.7	37.5	13.7	8.5
DIF	0.2	0.0	0.0	0.0	0.2
ISSSTELEON	0.2	4.0	4.2	0.0	0.4
ISSSTE	3.9	5.3	4.2	2.4	3.8
PEMEX	0.2	0.0	0.0	8.1	0.8
Particular	38.9	10.7	4.2	14.5	35.3
Cruz Roja	0.7	0.0	0.0	0.8	0.7
Hospital Civil	0.4	0.0	0.0	0.0	0.3
Otra institución(1)	22.2	4.0	4.2	3.2	19.7
No sabe	1.1	0.0	4.2	0.0	1.0
Total	100.0	100.0	100.0	100.0	100.0

Fuente: EESN-NL 2011/2012 (Cuestionario Hogar).
(1)En la encuesta también fueron incluidos otras instituciones (Marina/Defensa) que no fueron referenciados por los encuestados.

La EESN-NL 2011/2012 muestra un incremento en la atención privada respecto a la ENSANUT 2006, al pasar de un 22.1% a un 35.3% (Tabla 5.6).

Tabla 5.6 Población según utilización de servicios de salud ambulatorios por institución.

Instituciones	ENSANUT 2006 (Nuevo León)	EESN- NL 2011/2012
Particular	22.1%	35.3%
IMSS	43.2%	23.4%
Seguro Popular/SSA	27.1%	14.4%
ISSSTE	ND	3.8%
Otra institución(1)	7.6%	23.1%

Fuente: EESN-NL 2011/2012 (Cuestionario Hogar).
(1)MSS Oportunidades, SSNL, DIF, ISSSTELEON, MARINA/DEFENSA, PEMEX, CRUZ ROJA Y HOSPITAL UNIVERSITARIO (CIVIL).

En el año previo a la encuesta el 6.0% de la población refirió haber sido diagnosticado con alguna enfermedad crónica, destacando 1.4% con hipertensión y 1.3% diabetes. El comportamiento a nivel regional es equivalente en todas las regiones, prevaleciendo los mismos padecimientos (Tabla 5.7).

Tabla 5.7 Población según diagnóstico de enfermedades crónicas* en el último año, dividido por región geográfica.

Enfermedades	Región				Estatal
	Metropolitana	Norte	Sur	Centro	
	%	%	%	%	%
Asma	0.4	0.6	0.3	0.2	0.4
Hipertensión	1.2	3.0	3.1	2.1	1.4
Diabetes	1.0	2.1	1.7	2.3	1.3
Artritis	0.2	0.1	0.3	0.2	0.2
Enfermedad del corazón	0.1	0.3	0.3	0.2	0.1
Fiebre reumática	0.0	0.1	0.0	0.0	0.0
Enfermedad renal	0.2	0.1	0.0	0.0	0.1
Derrame cerebral	0.1	0.1	0.0	0.1	0.1
Depresión o ansiedad	0.1	0.1	0.0	0.1	0.1
Gastritis o úlcera gástrica	0.5	0.4	0.3	0.6	0.5
Colitis	0.2	0.0	0.0	0.7	0.2
Tumores, cáncer	0.1	0.1	0.0	0.2	0.1
Otra enfermedad con duración mayor a 3 meses(1)	1.8	1.5	0.7	1.3	1.7
No presenta	94.2	91.3	93.1	92.0	93.8
Total	100.0	100.0	100.0	100.0	100.0

Fuente: EESN-NL 2011/2012 (Cuestionario Hogar).
(1)En la encuesta también fueron incluidas otras enfermedades (Tuberculosis, Artosis y SIDA) que no fueron referenciadas por los encuestados.

Respecto a limitaciones físicas tales como debilidad visual o ceguera, auditiva, dificultad para moverse o algún retraso o deficiencia mental, el 6.5% refirió tener al menos una de éstas. En el plano regional encontramos porcentajes similares, siendo más alto en la Región Sur, que presenta un 7.5% de la población con diversas limitaciones. (Tabla 5.8).

Tabla 5.8 Población según limitación física o dificultad mental permanente, dividido por región geográfica.

Limitación física o deficiencia mental	Región				Estatal
	Metropolitana	Norte	Sur	Centro	
	%	%	%	%	%
Moverse, caminar o lo hace con ayuda	1.8	2.7	1.4	2.0	1.9
Usar sus manos o brazos	0.2	0.1	0.0	0.2	0.2
Es sordo o usa aparato para oír	0.3	0.8	0.7	0.4	0.3
Es mudo	0.0	0.1	0.0	0.3	0.0
Es ciego o sólo ve sombras	0.4	0.4	1.4	0.2	0.4
Algún retraso o deficiencia mental	0.4	0.4	0.4	0.4	0.4
Tiene otra limitación	3.6	0.8	3.2	0.8	3.1
No tiene limitación o deficiencia mental	93.0	94.1	92.6	95.8	93.4
No sabe	0.3	0.4	0.4	0.1	0.3
Total	100.0	100.0	100.0	100.0	100.0

Fuente: EESN-NL 2011/2012 (Cuestionario Hogar).

En el último año el 2.5% de la población donó sangre; de éstos 88.1% en apoyo a algún familiar o por participación en alguna campaña y 11.0% no especificó la razón de la donación. El comportamiento a nivel regional es semejante al reportado para el Estado. (Tabla 5.9).

Tabla 5.9 Población según motivo de donación de sangre en el último año, dividido por región geográfica.

Motivo de donación de sangre	Región				Estatal
	Metropolitana	Norte	Sur	Centro	
	%	%	%	%	%
Participación en una campaña	0.1	0.1	0.0	0.1	0.1
Apoyo a algún familiar	2.2	1.2	1.1	2.1	2.1
Obtener recursos económicos[(1)]	0.0	0.0	0.0	0.0	0.0
Otro	0.3	0.1	0.4	0.2	0.3
No	97.3	98.6	98.5	97.6	97.5
Total	100.0	100.0	100.0	100.0	100.0

Fuente: EESN-NL 2011/2012 (Cuestionario Hogar).
[(1)]A nivel estatal, menos del .02% dona sangre para obtener recursos económicos.

Gasto de utilización de servicios de salud

En cuanto a los gastos de utilización de los servicios de salud ambulatorios, preventivos y curativos, fueron evaluados varios aspectos. En cuanto a la percepción del costo de la atención recibida en las últimas dos semanas, el 79.3% lo consideró adecuado (lo pudo sufragar), 10.9% elevado (no lo pudo pagar) y 9.9% no pudo establecer una opinión. En el escenario regional, las regiones Metropolitana y Centro se comportaron como el nivel estatal, mientras que en las regiones Norte y Sur lo consideraron adecuado el 84.6% y el 100.0% respectivamente. El costo monetario de la atención se presenta en la Figura 5.3 y Tabla 5.10.

Figura 5.3. Población según costos de atención hospitalaria recibida.

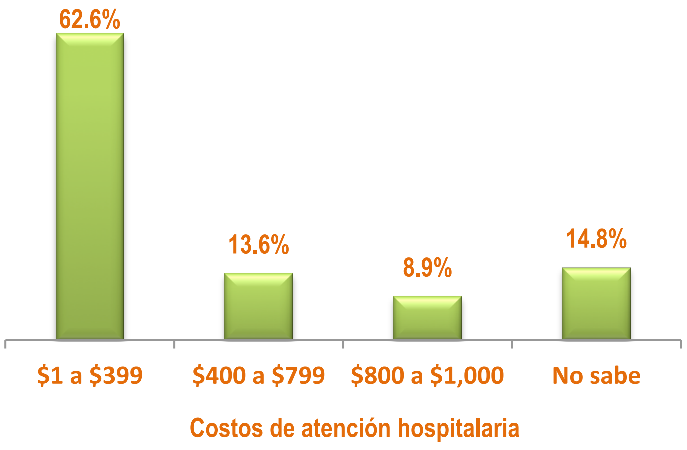

Fuente: EESN-NL 2011/2012 (Cuestionario Hogar).

Tabla 5.10 Población según costo de la atención hospitalaria recibida, dividida por región geográfica.

Costo de atención hospitalaria	Región: Metropolitana %	Región: Norte %	Región: Sur %	Región: Centro %	Estatal %
$1 a $399	61.8	64.3	100.0	70.3	62.6
$400 a $799	14.2	14.3	0.0	8.1	13.6
$800 a $1000	9.4	0.0	0.0	8.1	8.9
No sabe	14.7	21.4	0.0	13.5	14.8
Total	100.0	100.0	100.0	100.0	100.0

Fuente: EESN-NL 2011/2012 (Cuestionario Hogar).

En cuanto al número de consultas necesarias para finalizar el tratamiento, el 65.9% lo resolvió con la primera consulta, 22.7% en dos o tres, 8.6% en más de tres y el 2.9% no precisó la información. En el contexto regional, en la Región Sur el 100.0% de la población manifestó resolver su problema en solo una consulta, en el resto de las regiones el comportamiento fue similar al estatal. (Figura 5.4).

Figura 5.4 Población según número de consultas necesarias para finalizar tratamiento, dividido por región geográfica.

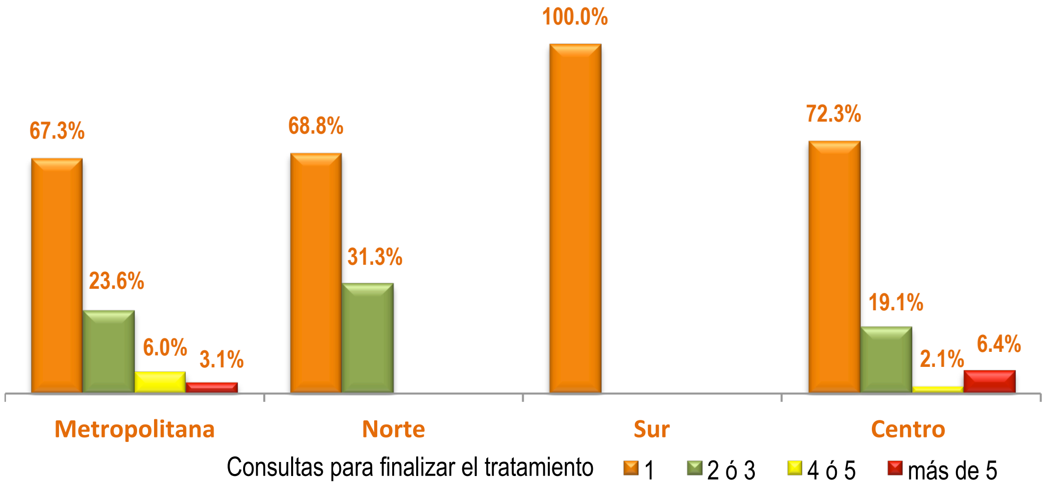

Fuente: EESN-NL 2011/2012 (Cuestionario Hogar).

En relación al Seguro Popular, 96.0% de los responsables del hogar manifestaron haber escuchado sobre él, mientras que a nivel regional se observó este mismo comportamiento. El 56.4% se enteró por los medios masivos de comunicación, destacando entre ellos la televisión, 24.2% por algún familiar o amistad, 16.7% por personal de salud y 2.7% por otros medios. En el contexto regional, las regiones Metropolitana y Centro se comportaron como el encontrado a nivel estatal, en la Región Norte el 32.4% se enteró por algún familiar, amistad o vecino, y en la Región Sur el 43.6% por personal de salud (Tabla 5.11).

Tabla 5.11 Población según medio por el que se enteró del programa del Seguro Popular, dividido por región geográfica.

Medio	Región				Estatal
	Metropolitana	Norte	Sur	Centro	
	%	%	%	%	%
Radio	3.3	4.9	13.8	8.1	4.2
Televisión	57.1	24.9	24.5	35.9	52.0
Periódico	0.3	0.0	0.0	0.0	0.2
Folletos, carteles	2.2	1.6	2.1	0.8	2.0
Personal de salud en una unidad médica	7.4	24.3	43.6	18.4	10.6
Familiar, amistad o vecino	23.0	32.4	7.4	32.3	24.2
Reuniones de información en su localidad	1.9	7.0	5.3	1.5	2.2
Vocearon	0.7	2.2	0.0	0.5	0.7
En su hogar a través de brigadas de Salud	3.4	2.7	3.2	2.0	3.2
Otro(1)	0.8	0.0	0.0	0.5	0.7
Total	100.0	100.0	100.0	100.0	100.0

Fuente: EESN-NL 2011/2012 (Cuestionario Hogar).
(1)No se especificó otro medio.

El 27.6% de las familias reportaron que al menos un miembro ha estado inscrito en el Seguro Popular, a nivel regional las áreas con mayor adscripción fueron la Región Sur y la Región Norte con 85.6% y 58.2% respectivamente.

Si los servicios médicos fueran gratuitos el 44.1% preferirían atenderse en los servicios médicos particulares, 18.4% en el IMSS, 12.9% en el Seguro Popular, 5.4% en la Secretaría de Salud y 19.2%, en otra institución (ISSSTELEON, ISSSTE, Marina/Defensa, PEMEX). En las regiones Metropolitana y Centro los entrevistados manifestaron su preferencia por los servicios médicos particulares, mientras en las regiones Norte y Sur por el Seguro Popular (Tabla 5.12).

Tabla 5.12 Población según preferencia de institución de servicios médicos en el supuesto de ser gratuitos, dividido por región geográfica.

Institución	Región				Estatal
	Metropolitana	Norte	Sur	Centro	
	%	%	%	%	%
IMSS	17.6	16.4	9.4	27.3	18.4
SSA	3.9	14.7	11.1	9.8	5.4
Seguro Popular	9.9	32.8	35.0	18.4	12.9
ISSSTELEON	1.8	0.4	1.7	0.2	1.6
ISSSTE	4.0	1.7	6.8	3.6	3.9
Marina / Defensa	1.5	0.4	0.9	0.7	1.3
PEMEX	1.2	0.0	0.9	4.0	1.5
Particular	47.5	27.2	32.5	31.3	44.1
Otra institución	3.4	1.7	0.9	1.3	3.0
No sabe	9.0	4.7	0.9	3.3	7.9
Total	100.0	100.0	100.0	100.0	100.0

Fuente: EESN-NL 2011/2012 (Cuestionario Hogar).
*No se especificó otro medio.

Programas de ayuda alimentaria

En relación a los programas de ayuda alimentaria 4.4% manifestaron recibir algún tipo de apoyo, lo que representa a 171,326 personas: de estos el 95.6% recibió ayuda en los dos meses previos a la aplicación de la encuesta, 63.4% recibe apoyo desde hace un año, 26.6% indicó recibirlo mensualmente y 45.8% bimestralmente. (Tabla 5.13).

Tabla 5.13 Población según programas de ayuda alimentaria que recibe, dividido por región geográfica.

Programas	Región				Estatal
	Metropolitana	Norte	Sur	Centro	
	%	%	%	%	%
Despensas de los programas alimentarios DIF	1.7	5.5	7.1	4.1	2.3
Comedores DIF	0.1	0.4	0.6	0.0	0.1
Desayunos escolares DIF	0.3	0.5	5.3	1.9	0.6
Programa de ayuda alimentaria a sujetos vulnerables	0.4	0.7	1.6	1.3	0.5
Programa Papilla Maíz	0.1	0.1	0.6	0.1	0.1
Servicios de Cáritas	0.5	0.0	2.6	0.1	0.5
Otras ONG´s(1)	0.1	0.4	0.1	0.4	0.1
Total	3.2	7.6	17.9	7.9	4.2

Fuente: EESN-NL 2011/2012 (Cuestionario Hogar).
ONG's: Organizaciones No Gubernamentales.
(1)No se especificó otra organización.

Resultados relevantes

- Alrededor del 90.0% de la población manifestó estar afiliada a un servicio médico, siendo el principal proveedor de la atención médica el IMSS.
- Con respecto a los problemas de salud manifestados quince días previos a la aplicación de la encuesta, 20 de cada 100 personas presentaron alguna enfermedad, siendo las respiratorias las más frecuentes, 66 de cada 100 resolvieron la problemática de salud con una sola consulta, el 11.0% no pudo pagar la atención médica por considerar elevado su costo.
- El 96.0% de los encuestados conoce el programa del Seguro Popular, siendo los medios masivos de comunicación el instrumento de mayor difusión para el conocimiento de este programa.
- En lo referente a la condición de adscripción al servicio médico, ENSANUT 2012 reportó que 25.4% no contaba con ella, cifra que disminuye a 13.2% en la EESN-NL 2011/2012.
- El porcentaje de población sin adscripción al servicio médico es menor en comparación al ámbito nacional, cabe mencionar que la encuesta se aplicó previo a la campaña de afiliación del Seguro Popular.
- En los 51 municipios del Estado de Nuevo León se encuentran distribuidos los programas de ayuda alimentaria, para atender las necesidades de la población con inseguridad alimentaria y vulnerable, donde participan instituciones gubernamentales y organizaciones de la sociedad civil; el dato mostrado en la encuesta muestra cifras inferiores a las reportadas por estas dependencias, lo que supone omisión de la información por los encuestados, por razones no conocidas.
- Las regiones Metropolitana y Centro presentan mayor población afiliada al IMSS, mientras que en las regiones Norte y Sur se encontró mayor afiliación al Programa del Seguro Popular.
- La televisión es el medio de comunicación por el cual se entera el mayor porcentaje de la población de las regiones Metropolitana y Centro sobre el programa del Seguro Popular, en la Región Norte es por algún familiar, mientras que en la Región Sur es por el personal de salud.
- La Región Sur del Estado presenta el mayor porcentaje de la población beneficiada por los programas de ayuda alimentaria existentes.

Conclusiones

EESN – NL 2011/2012

Conclusiones

La Encuesta Estatal de Salud y Nutrición – Nuevo León 2011/2012, nos muestra un panorama general respecto a las condiciones actuales del Estado según sus características sociodemográficas, salud de la población, nutrición, alimentación y servicios de salud. Esta es la primera encuesta que se realiza en Nuevo León y la primera también que realiza una entidad a nivel nacional. La EESN-NL 2011-2012 aporta información a partir de la cual es posible hacer inferencias no solo a nivel estatal, sino también en el ámbito regional, por lo que la consideramos una gran fortaleza para la correcta toma de decisiones en materia de salud y nutrición.

En esta primera encuesta queda claro que dentro de las principales características de Nuevo León, podemos decir que las viviendas cuentan con alta cobertura (más de 90.0%) de servicios básicos (luz, agua, gas, drenaje), las cuales representan factores protectores para la salud. La población en el Estado es joven, y el mayor porcentaje se concentra entre los 0 y 15 años de edad, de los cuales el 99.0% acude a la escuela. Esta característica de la población sugiere mayor atención en los servicios que se ofrecen a este grupo de edad, sin pasar por alto al grupo de 60 años y más. El promedio de habitantes por hogar en Nuevo León es de 3.7 individuos y la distribución de sus viviendas es considerado adecuado al tener una distribución de dos o tres habitaciones independientes al área de cocina. Los bienes más comunes presentes en el hogar son los electrodomésticos como el refrigerador, estufa, lavadora, licuadora y televisor, además de tener un amplio acceso a los medios de comunicación interactivos como el celular y la computadora, lo que constituye un tema importante para la agenda de salud pública.

Respecto al estado de salud de la población, la encuesta muestra un panorama general de la situación de salud de niños y adultos de Nuevo León, que permite identificar áreas de oportunidad en las diferentes acciones encaminadas a la atención de la salud, promoviendo la equidad e incrementando la calidad de vida. Entre los hallazgos más relevantes en esta encuesta destacan la alta prevalencia de sobrepeso y obesidad en adultos mayores de 20 años, así como también la inactividad física, factores asociados a la aparición de enfermedades no transmisibles como la diabetes, hipertensión y algunos tipos de cáncer, entre otras. Ante la problemática de salud y nutrición en todos los grupos de edad, los programas que también merecen el fortalecimiento de sus acciones son: Vacunación, con acciones que logren una amplia cobertura de esquemas completos

en todos los menores, así como las acciones preventivas de enfermedades diarreicas e infecciones respiratorias agudas, y los programas de embarazo y alimentación al seno materno.

Por la alta concentración de población adolescente, es importante reforzar los programas orientados a este grupo de edad, principalmente en el diseño de programas de intervención educativos y de difusión en cuanto a salud reproductiva y estilos de vida saludables, principalmente nutrición, actividad física y consumo de alcohol y tabaco. En el grupo de 60 años y más es importante fortalecer los programas de detección oportuna y control de enfermedades no transmisibles como la diabetes *mellitus* y la hipertensión arterial, además de la identificación de los indicadores de depresión, fragilidad y deterioro cognitivo, para garantizar una mejor calidad de vida a los adultos mayores.

En cuanto al estado nutricio, en niños menores de 5 años el problema de la desnutrición aguda se ha estabilizado y ha disminuido la desnutrición crónica. En el otro extremo, el sobrepeso y la obesidad se incrementaron en una década en escolares, adolescentes y adultos. Además, todas las cifras de obesidad son mayores al promedio nacional, excepto la obesidad abdominal en mujeres. Por primera vez, en una encuesta estatal en el país se presentan datos preliminares y específicos de la situación nutricional y de salud de los adultos mayores de 60 y más años. Este grupo de edad requiere un seguimiento distinto de sus necesidades de nutrición por los programas de salud del Estado.

Respecto a la alimentación de la población, cabe destacar que en Nuevo León, existen datos generados en el Diagnóstico Nutriológico de las Familias Menores de 5 años de Nuevo León 2000, que nos permiten visualizar su evolución a casi doce años de distancia, los datos muestran que el 50.0% o más de la población consume 19 alimentos más y que en total para la construcción de la Canasta Básica Alimentaria estarían disponibles 48 alimentos más que en el año 2000, pero se tienen que analizar otros elementos como necesidades energéticas, hábitos alimentarios, calidad, variedad y valor de los alimentos. Por otra parte, es importante considerar la Frecuencia Semicuantitativa de Consumo de Alimentos que nos permita estimar la ingesta de energía y nutrientes de la población, así como la evaluación de las guías alimentarias en México, como la NOM 043, que refiere que una dieta variada debe contener en cada tiempo de comida al menos un alimento de cada uno de los grupos de alimentos, lo que significaría la necesidad de un número mayor a 40 alimentos que debe consumir un alto porcentaje de la población.

Lo anterior puede llevar a la observación de que si bien en 11 años, la variedad de alimentación en la población puede haberse incrementado, aún queda por revisar el grado de cumplimiento de la citada Norma.

En cuanto a la utilización de servicios de salud en Nuevo León, la mayoría de la población es atendida por el Instituto Mexicano del Seguro Social (IMSS), pero cabe destacar que la información recabada es previa a la campaña de cobertura total por el Seguro Popular, la cual ha influido en la disminución del porcentaje de personas que refieren no contar con adscripción a un servicio médico según la ENSANUT 2012. En el Estado permanecen vigentes programas de ayuda alimentaria dirigidos por instituciones gubernamentales y organizaciones de la sociedad civil, que tienen presencia en todos los municipios del Estado, para atender la problemática de la inseguridad alimentaria.

Los resultados de la Encuesta Estatal de Salud y Nutrición – Nuevo León 2011/2012 (EESN – NL 2011/2012), han permitido diseñar programas y políticas de alimentación, nutrición y salud, con acciones específicas dirigidas a los grupos de población en riesgo, entre ellos, los programas estratégicos que se han desarrollado en el marco del Programa de Alimentación Saludable y Actividad Física para la Prevención de la Obesidad y el Sobrepeso (PASOS) como: Quítate un Peso de Encima, Salud Para Aprender y Establecimientos de Consumo Escolar (ECoEs), entre otros.

La información que aporta la EESN – NL 2011/2012 es confiable para la correcta toma de decisiones desde un ámbito estatal y local, además de proveer información a la comunidad científica para la generación y divulgación de conocimiento que contribuirá a mejorar la salud y nutrición de la población.

Bibliografía

A Rissanen, P. K. (1991). Weight and Mortality in Finnish Women. *J Clin Epidemiol, 44* (8), 787-795.

Alison Jacknowitz, D. N. (2007). Special Supplemental Nutrition program for Women, Infants, and Children and Infant Feeding Practices. *Pediatrics* (119), 281-289.

Astrid Lang, E. S. (2006). Management of Overweight and Obesity in Adults: Behavioral Intervention for Long-Term Weight Loss and Maintenance. *European Journal of Cardiovascular Nursing* (5), 102-114.

Bhan MK, B. R. (2003). Management of the Severely Malnourished Child: Perspective from Developing Countries. *BMJ* (326), 146-151.

Breeze E, C. R. (2006). Cause-specific mortality in old age in relation to body mass index in middle age and old age: follow-up of the Whitehall cohort of male civil servants. *Int J Epidemiol, 35*, 169-78.

Chumlea WC, G. S. (1998). Stature prediction equations for elderly non-Hispanic white, non-Hispanic black, and Mexican-American persons developed from NHANES III data. *J Am Diet Assoc, 98* (2), 137-142.

De Onis M, O. A. (2004). Measurement standarization protocols for anthropometry used in the construction of a new international growth reference. *Food Nutr Bull, 25*, S27-S36.

De Onis M, O. A. (2007). Development of a WHO growth reference for school-aged children and adolescents. *Bull WorldHealthOrgan, 85*, 660-7.

Dylan Harris, N. H. (2005). Malnutrition screening in the elderly population. *J R Soc Med, 98*, 411-414.

Facultad de Salud Publica y Nutrición, UANL/Secretaría de Salud. (2000). Diagnóstico Nutriológico de las Familias y Menores de 5 años del Estado de Nuevo León 2000.

Gobierno de Honduras. (2010). Secretaría del Trabajo y Seguridad Social. Recuperado de http://www.gob.hn.

Gobierno de Nuevo León. (2011). Secretaría de Economía. Recuperado de http:// www.nl.gob.mx/economia.

INEGI. (2005). Conteo de Población y Vivienda 2005. www.inegi.org.mx

INEGI. (2009). Encuesta Nacional de Dinámica Demográfica 2009. http://www.inegi.org.mx.

INEGI. (2010). Censo General de Población y Vivienda 2010. www.inegi.org.mx.

Instituto Nacional de Salud Pública-Secretaría de Salud. (2007). Resultados por entidad federativa, Nuevo León. *Encuesta Nacional de Salud y Nutrición 2006.*

Instituto Nacional de Salud Pública-Secretaría de Salud. (2012). *Encuesta Nacional de Salud y Nutrición 2012.* Resultados nacionales. 2012.

Jain, A. (2005). Treating obesity in individuals and populations. *BMJ* (331), 1387-1390.

Jan-Magnus Kvamme, J. H. (2012). Body mass index and mortality in elderly men and women: the Troms and Hunt studies. *J Epidemiol Community Health, 66*, 611-617.

Kennedy G, N. G. (2006). Assessment of the double burden of malnutrition in six case study countries. In: The double burden of malnutrition. Case studies from six developing countries. (F. a. Nations, Ed.) *FAO Food and Nutrition Paper* (84), 1-18.

López Ramírez Adriana (2001). *El perfil sociodemográfico de los hogares en México 1976-1997,* abril de 2001. México, DF: Consejo Nacional de Población.

Martínez, J. A., Astiasarán, I., & Madrigal, H. (2002). *"Alimentación y Salud Pública"*. Madrid: McGraw-Hill.

Martorell R, K. L. (1994). Reversibility of stunting: epidemiological findings in children from developing countries. *Eur J Clin Nutr* (48), 45-57.

Menchú E., M. T. (2002). *La canasta básica de alimentos en Centroamérica: Revisión metodológica.* Instituto de Nutrición de Centro América y Panamá. Guatemala: Organización Panamericana de la Salud.

Norma Oficial Mexicana Nom-043-SSA2-2005, Servicios Básicos de Salud. Promoción y educación para la salud en materia alimentaria. Criterios para brindar orientación.

Pfeiffer, E. (1975). A short portable mental status questionnaire for the assessment of organic brain deficit in elderly patients. *Journal of the American Geriatric Society* (23). 433-441.

Ramos Peña, E. G. (2009) *Consumo alimentario en relación a los determinantes socio - demográficos y una propuesta de política alimentaria en Nuevo León.* Monterrey, Nuevo León: Universidad Autónoma de Nuevo León.

Ramos, P. E., Castro, S. A., & Zambrano, M. A. (2009). Consumo de alimentos en la población, desde las perspectivas socionutricia y económica. En D. S. Moisén, & D. L. Anguiano, *"Formación de recursos humanos investigación y sistemas de Salud"* (págs. 181-197). Durango: Universidad Juárez del Estado de Durango.

Secretaría de Salud. (2010). Acuerdo Nacional para la Salud Alimentaria. Estrategia contra el sobrepeso y la obesidad.

Secretaría de Salud. (2006). Diario Oficial del 23 de enero del 2006. 32-49. Recuperado de http://www.salud.gob.mx.

Secretaría de Salud de Nuevo León / Universidad Autónoma de Nuevo León / Facultad de Salud Pública y Nutrición / Facultad de Medicina / Sistema para el Desarrollo Integral de la Familia del Estado de Nuevo León / Cáritas de Monterrey, ABP / Secretaría de Educación de Nuevo León. Síntesis Ejecutiva. Encuesta Estatal de Salud y Nutrición – Nuevo León 2011/2012. Monterrey, N. L. México, 2012.

The IDF consensus worldwide definition of the metabolic syndrome. (2011). International Diabetes Federati Recuperado de http://www.idf.org.

Victoria CG, A. L. (2008). Maternal and child undernutrition: consequences for adult health and human capital. *Lancet* (371), 340-357.

Visser, M. (2009). Towards a definition of sarcopenia - results from epidemiologic studies. . *J Nutr Health Aging, 13* (8), 713-6.

WHO. (1995). Physical status: the use and interpretation of anthropometry. Report of a WHO Expert Committee. *WHO Technical Report Series 854*.

WHO. (2000). Obesity: preventing and managing the global epidemic. Report of a WHO Consultation. *WHO Technical Report Series 854*.

WHO. (2006). WHO Child Growth Standards: Methods and development: Length/height-for-age, weight-for-age, weight-for-length, weight-for-height and body mass index-for-age. Geneva: World Health Organization.

WHO. (2006). Training Course on Child Growth Assessment. Version 1.

Anexo 1. Encuestadores

Abigail Isaac Santos, TSC.
Alan Alberto Cantero Jacobo, Lic.
Amado Mata Vázquez
Ana Karen Sánchez Martínez, Prom.
Antonio Coronado Vázquez, Lic.
Antonio Dávila García, LN.
Armando Javier de los Santos Rodríguez, T.P.
Jesús Armando Torres Liñán, Prom.
Bárbara Liliana Vilchis Mata
Bertha Alicia Garza Galaviz, Lic.
Blanca Diocelina Machado Fabela
Blanca Lilia Peña Rodríguez, Q.C.B.
Brenda Gabriela Lozano Castillo, L.T.S.
Brenda Isela Roque de la Rosa, Dra.
Brenda Yadira Pérez Méndez, LN.
Carlos Alberto Luna Rivas, Lic.
Cecilia Torres Morales, LN.
Cinthia Judith Gómez Cuevas
Cipatly Adriana Cortés Dueñas, LN.
Claudia Patricia Rivera Zavala, Prom.
Dalia Elisa Lazcano Tijerina, Q.C.B.
Daniel Leal Hernández, Dr.
Daniel Orlando Garza Cázares
Dayana Espinoza Mendoza, Enf.
Desiderio Martinez Torres, Enf.
Diana Guadalupe Enriquez Niño, Enf.
Dolores Rita de Jesús Aguilar Jiménez, Lic.
Edith Banda Martínez, Prom.
Elizabeth Romero Rocha
Emanuel de Hoyos Cervantes, T.L.C.
Erika Maricela Hernández Rodríguez
Ernestina Pérez Reyes, Lic.
Eudelia Jasso Garza
Fernando Cantú Salazar, Prom.
Francisco Javier de Luna Álvarez, T.P.
Francisco Mariño Hernández Flores, LN.
Gabriela Alejandra Álvarez Cadena, LN.
Gabriela Arriaga Carrión, Profa.
Gilberto Brambila Treviño, T.P.
Gonzalo Guerra Espinoza, Prom.
Grace Kelly de Jesús García Flores
Griselda Abigail Niño Villarreal, LN.
Gustavo Medellín Compeán, Prom.
Iliana Yaneth Casa Gaona, Lic.
Imelda Marisol Mata Castillo, Enf.
Isaac Flores Zúñiga
Isai Ledezma Aldaba
Isenia Guadalupe Obregón Rodríguez, Enf.
Iván Oswaldo Medina Beltrán, LC.
Ivonne Hinojosa Alcorta, Lic.
Jaime Ezequiel Pérez Orozco, Lic.
Javier Delgado Rodríguez Dr.
Javier Suro Amezaga, Lic.
Jesús Edson Guevara Álvarez
Jesús García Longoria, T.L.C.
Jesús Isidro Morales Godoy, L.S.
José Domingo Muñoz González
José Luis Cordero, Lic.
Juan de Dios Valdez López
Karla Carolina Navejar Ibarra, LN.
Laura Ordaz de la O, LN.
Leticia de los Santos Ábrego, Lic.
Leticia Gaona Escobedo, Enf.
Lilia Rosa Gutiérrez Terrazas, LN.
Lucy Denise Rosas Sandoval, LN.
Luis Alberto Pantoja Luna, Prom.
Luz Mabel Betancourt Campos, Prom.
Ma. Guadalupe Benavides Martínez
Marco Antonio Moctezuma Espiricueto
Marco Antonio Torres Guerrero, Lic.
María Apolinar Rodríguez Flores, Enf.
María Aurora Balderas Almanza, LN
María Concepción Sandoval Guajardo, Enf.
María de los Reyes Martínez Rodríguez, Enf.
María del Carmen Cantú Rodríguez
María del Carmen Ramírez Acevedo
María Guadalupe Macías de la Rosa, LN.
María Stephanie Cid Gallegos, LN.
María Teresa Sepúlveda González
Maricela García Almanza, LP.
Mario Guajardo Juárez, Lic.
Maritza Banda Duarte, Enf.
Martina Castellanos Martínez
Mayra Cecilia Guerrero Martínez, LN.
Mayra Janeth Arizpe Quintanilla, LN.
Milory Yedit Alanís Ríos, Lic.
Missael Fernando Morales Zavala, PESS.
Mucio Mauricio Longoria, Prom.
Myrna Angélica Rico Rodríguez
Nadia Melissa Hernández Medina, LN.
Nancy Judith Garza González, Lic.
Natalia Aguilar Ramos
Nora Alejandra Montes Núñez, LN.
Norma Alicia Leal Garza
Óscar Edgardo Ortega Arguelles
Patricia de Jesús Mireles Hernández, Enf.
Paulina Aparicio Cárdenas, LN.
Pedro López Hernández, Ing.
René Paul González Elizondo, LN.
Reyna Belén Ríos Garza, LN.
Roberto Carlos Aguirre Salazar
Rocío Espinoza Viramontes
Rolando Ibarra García, Dr.
Rubén Toledo Pérez, Lic.
Sandra Guadalupe Flores Macías, Tec.
Selene Yamilet Acosta Arreazola, Prom.
Sonia Alonso Cruz, Enf.
Sonia Francisca Mariscal Ibarra
Teodora Bermúdez Escalante, QBP.
Verónica Ramos Salas, LN.
Vianey Ponce Amaya, Lic.
Vicente Guerrero Martínez, Lic.
Virginia Elena Flores Guerra, Tec. T.S.
Yenisei Ramírez Toscano, LN.
Yubia Esmeralda Ramírez Olivares, Lic.
Zintia Manu Ramírez Soto, Enf.

Anexo 2. Participantes en validación de base de datos

Alejandra Janeth Ramones Zazueta
Amanda Ramos Trujillo
Anaid Solís Flores
Andrea Alejandra Muñoz Vázquez
Belén Hernández Silva
Brenda Marisol Mendoza Ramírez
Carolina de la Garza Rivera
Carolina María González Ramos
Cinthya Alejandra Garza González
Cinthya Madeline Villarreal Mireles
Daniela Alejandra Mata Cardona
Diana Alejandra Menchaca Romo
Diana Rocío Martínez Zambrano
Edith Guadalupe Coronado Sánchez
Elsa María Sánchez Fraire
Grace Juliana Ibarra Corral
Irma del Rosario Longoria Flores
Isis Liliana Mendoza Garza
Jessica Torres Anguiano
Judith Estefany Garza Márquez
Julia Catalina Cantú Salgado
Karen Erín Cabrera Guerra
Karen Guadalupe Contreras Ovando
Karina Lisette Martínez
Karla Nayeli Martínez Domínguez
Karla Patricia Ruiz Juárez
Keila Irasema Caballero Rodríguez
Laura Irene Canales Garza
Laura Patricia Garza Montemayor
Laura Stephanie Carranco Paz
Liseth Arylu Peña Bolaños
Lucía Alejandra Villarreal Jiménez
Luisa Méndez Zapata
Marcela W. Martínez Escalante
Marco Iván Cantú Juárez
María Alejandra Villarreal Pérez
María Elena Trujillo Manzanares
Mariana Caballero Arredondo
Marisol Solís Castillo
Martha Alejandra Rodríguez Ríos
Massara Michelle de la Rosa Rodríguez
Mayra Elisa Sánchez Murillo
Mayra Judith Rodríguez González
Mónica Carolina Carrión Treviño
Mónica Sanjuana Mora Olvera
Mónica Trejo Rodríguez
Myriam Leticia Elizondo Martínez
Nallely E. Reyes Monsiváis
Nancy Denisse Flores Castillo
Nataly Cavazos Tamez
Noelia Gabriela Guerra González
Nydia Nallely de la Fuente Martínez
Olga Marléen Garza Rojas
Pamela Lizeth González González
Patricia M. Morales Campos
Perla Cecilia Barrientos Guerrero
Ramiro Iván Campos Romero
Raziely Quintanilla González
Roxana Nataly Cavazos Tamez
Ruth Estefanía Vázquez Garza
Silvia María Galaviz Alarcón
Wendy Elizabeth Villarreal Salinas
